# Novel compound heterozygous mutations of *ALDH1A3* contribute to anophthalmia in a non-consanguineous Chinese family

**DOI:** 10.1590/1678-4685-GMB-2016-0120

**Published:** 2017-06-05

**Authors:** Yunqiang Liu, Yongjie Lu, Shasha Liu, Shunyao Liao

**Affiliations:** 1 Department of Medical Genetics, West China Hospital, West China Medical School, Sichuan University, Chengdu, China; 2 Diabetes Center & Institute of Transplantation, Sichuan Academy of Medical Science & Sichuan Provincial People's Hospital, School of Medicine, University of Electronic Science and Technology of China, Chengdu, China

**Keywords:** Anophthalmia, exome sequence, ALDH1A3, compound heterozygous mutations

## Abstract

Anophthalmia is a rare eye development anomaly resulting in absent ocular globes or tissue in the orbit since birth. Here, we investigated a newborn with bilateral anophthalmia in a Chinese family. Exome sequencing revealed that compound heterozygous mutations c.287G > A (p.(Arg96His)) and c.709G > A (p.(Gly237Arg)) of the *ALDH1A3* gene were present in the affected newborn. Both mutations were absent in all of the searched databases, including 10,000 in-house Chinese exome sequences, and these mutations were confirmed as having been transmitted from the parents. Comparative amino acid sequence analysis across distantly related species revealed that the residues at positions 96 and 234 were evolutionarily highly conserved. *In silico* analysis predicted these changes to be damaging, and *in vitro* expression analysis revealed that the mutated alleles were associated with decreased protein production and impaired tetrameric protein formation. This study firstly reported that compound heterozygous mutations of the *ALDH1A3* gene can result in anophthalmia in humans, thus highlighting those heterozygous mutations in *ALDH1A3* should be considered for molecular screening in anophthalmia, particularly in cases from families without consanguineous relationships.

## Introduction

Anophthalmia and microphthalmia (A/M, OMIM 206900) are rare inborn defects of eye development and show a phenotypic continuum from the complete absence of the eye globes (A) to the small eyes (M), as defined in terms of axial length and corneal diameter. A/M can be isolated or associated with other anomalies. Anophthalmia rarely occurs in isolation, with its birth prevalence ranging from 0.6 to 4.2 per 100,000 births (Skalicky *et al.*, 2013).

Genetic mutations are proposed as a predominant etiology for these ocular global anomalies (Bermejo and Martinez-Frias, 1998). The genetic foundations of A/M were demonstrated with a high degree of heterogeneity, including chromosomal abnormalities and monogenic mutations. Mutations in over 20 genes have been reported to contribute to A/M with dominant, recessive, or X-linked inheritance patterns (Chassaing, 2014). Among these, the *SOX2* (OMIM 184429) and *OTX2* (OMIM 600037) gene mutations are the major cause of A/M, each accounting for approximately 10–20% and 4–8% of dominant cases, respectively (Schneider *et al.*, 2009; Schilter *et al.*, 2011), and the *FOXE3* (OMIM 601094) gene mutation is a common source of recessive microphthalmia and explains approximately 15% of the dominant cases (Reis *et al.*, 2010). Recently, the *ALDH1A3* gene (OMIM 600463) mutations were revealed to underlie autosomal recessive A/M and was estimated to be responsible for approximately 10% of the cases in consanguineous families (Fares-Taie *et al.*, 2013; Abouzeid *et al.*, 2014). Despite this progresses in understanding the genetic basis of A/M, more than 50% of A/M patients still have unknown causes.

Next-generation sequencing with exome selection has been successfully employed to identify the causative genes mutations in genetically heterogeneous disorders. Whole-exome sequencing (WES) has been shown to be effective in both screening known genes and searching for new causative factors in families with A/M (Aldahmesh *et al.*, 2012; Fares-Taie *et al.*, 2013; Yahyavi *et al.*, 2013; Slavotinek *et al.*, 2015). Herein, we investigated a newborn boy with bilateral anophthalmia in a non-consanguineous Chinese family using WES and further confirmed the causative gene by bioinformatics and *in vitro* expression analyses.

## Subjects and Methods

### Subjects

**Figure 1:**
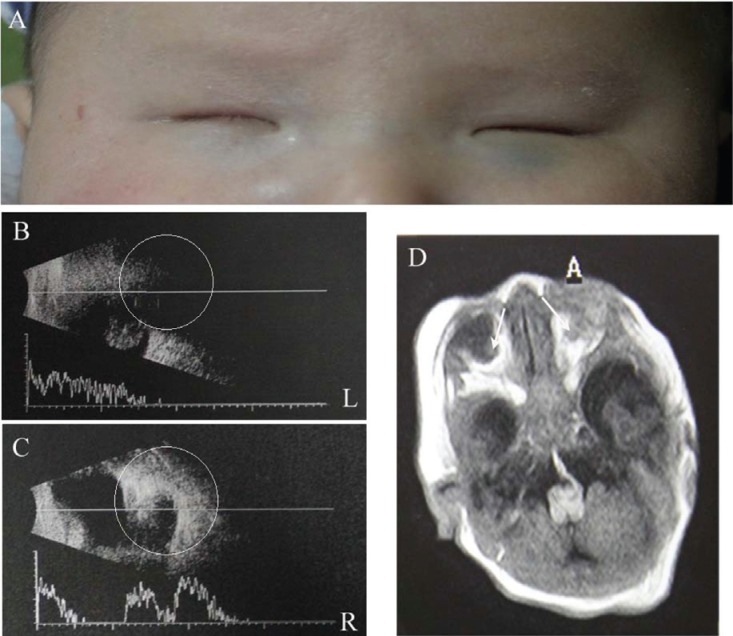
Clinical and imaging features of the affected boy. A. Eyes of the boy affected with anophthalmia. B and C. Ultrasonography shows an anophthalmic socket on the left orbit and some vitreous cysts present in the right orbit, indicated circles. D. MRI shows an anophthalmic socket and remnant fibrotic tissue in the intraorbital region and hypoplastic optic nerve bilaterally, indicated by arrows (Axial T2-weighted MR image with fat-suppression).

**Figure 2:**
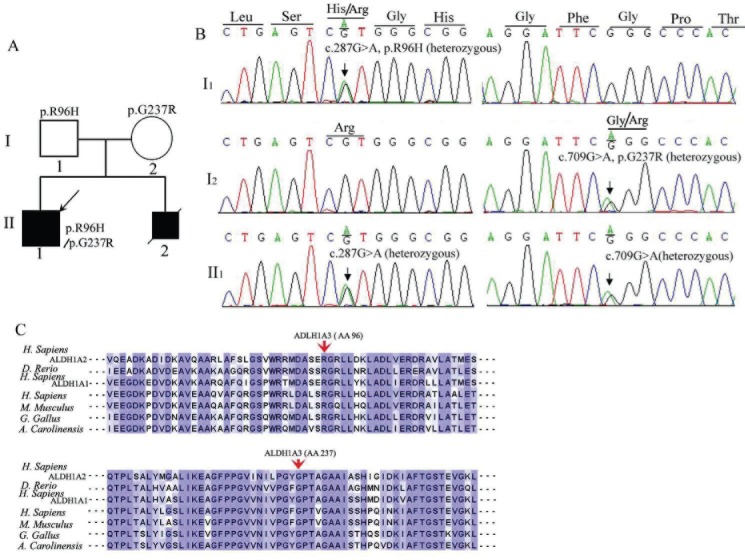
Family pedigree of the anophthalmia case and the mutation transmission of the *ALDH1A3* gene. A. Family pedigree of the case. B. Sequence analysis shows that the father (I1) was heterozygous at c.287G > A in *ALDH1A3* exon 3, the mother (I2) was heterozygous at c.709G > A in *ALDH1A3* exon 7, and the affected boy (II1) was heterozygous at both sites. C. Comparison of the amino-acid sequences near the R96 and G237 of ALDHs from different organisms (NCBI accession numbers: *H. sapiens* ADLH1A3: NP_000684, ADLH1A1: NP_000680, ADLH1A2: NP_733798, *D. rerio*: NP_001038210, *M. musculus*: NP_444310.3, *G. gallus*: NP_990000.1, and *A. carolinesis*: ENSACAT00000009770). The red arrows indicate the sites of mutated amino acids.

The family included in this study is of Han Chinese origin and resides in Chengdu City of Sichuan Province. The proband was a 25-day-old newborn boy with anophthalmia. His examination showed an absence of eyes, short eyelids and reduced palpebral fissures (Figure 1A). Ocular ultrasonography revealed that the posterior segments of both eyes were malformed and reduced in size, the ocular walls were irregular in shape and the left-sided optic disk region was abnormally depressed; some vitreous cysts were detected in the right orbit (Figure 1B, C). Cerebral magnetic resonance imaging (MRI) at 1 week of age displayed seriously deformed eye globes without well-defined borders, and the lenses were missing on both sides (Figure 1D). The boy was born by vertex vaginal delivery at full term. He passed the newborn hearing screen. Both young parents were phenotypically normal and reported no history of ocular abnormalities in their family members; however, the parents reported that their first fetus was terminated due to similar eye defects after ultrasonic inspection during pregnancy. The pedigree is shown in Figure 2A.

The current study was reviewed and approved by the Research Ethics Committee of the West China Hospital, West China Medical School, Sichuan University. Informed written consent was obtained from both parents, and consent was obtained on behalf of their son.

### Whole-exome sequencing (WES)

Blood was collected from all family members and genomic DNA was extracted according to standard procedures. The genomic DNA of the affected boy was randomly fragmented into an average size of 100~300 bp and ligated with a pair of linkers at both ends. The fragmented DNA was amplified by ligation-mediated polymerase chain reaction (LM-PCR) and hybrid-ized to a NimbleGen probe capture array (SeqCap EZ Exome Kit v3.0, covering more than 20,000 genes in the human genome, Roche NimbleGen, Madison, WI, USA). The captured LM-PCR products were subjected to quantitative PCR to estimate the magnitude of enrichment and were then loaded onto the Illumina Hiseq2500 platform (Illumina, San Diego, CA) for next-generation sequencing. Two parallel reactions were conducted.

### Genetic variations analysis

The sequencing read depth was 160.94 x on average, and the mean coverage was 99.76%. Low-quality variations were filtered out using a quality score ≥ 20 (Q20). Sequencing reads were aligned to the NCBI human reference genome (hg19) using Burrows-Wheeler Aligner. Single nucleotide polymorphisms (SNPs) and insertion/deletion (indel) of the sequence were analyzed using SAMtools and Pindel. All genetic variations were screened in the dbSNP147, Exome Variant Server, 1000 Genomes and in-house 10,000 Chinese exome database (Joy Orient, Beijing, China) to exclude common variants. Each rare missense mutation (MAF < 0.01) was tested for potential pathogenicity using SIFT (http://sift.jcvi.org/) and Polyphen-2 (http://genetics.bwh.harvard.edu/pph2/). The candidate gene variations were also searched in the Online Mendelian Inheritance in Man database (OMIM, http://www.omim.org/) and the human gene mutation database (HGMD, http://www.hgmd.cf.ac.uk/). Sanger sequencing was used to verify the variations of candidate genes in the affected baby and his parents.

### *In vitro* expression analysis of the *ALDH1A3* mutations

To examine the effects of *ALDH1A3* mutations, two sets of expression vectors were constructed based on the commercial plasmids pReceiver-M45 and -M46 (Genecopoeia, Rockville, MD), each set was tagged with HA and FLAG, respectively. All constructs were verified by sequencing.

The constructed plasmids were transfected into the 293T cells using a jetPRIME transfection kit (Polyplus, Illkirch, France). After 48 hours, the whole RNAs of each well were extracted, and the mRNA levels of wild type and mutant *ALDH1A3* transcripts were examined by quantitative reverse transcription PCR (qRT-PCR) analysis. In addition, the whole protein lysates from each well were extracted and analyzed using immunoblotting (IB) with the anti-HA (Abcam, Cambridge, MA) and anti-FLAG (Sigma-Aldrich, St. Louis, MO) antibodies, respectively. Briefly, an IB analysis involved the following steps: the protein lysates were separated on 10% SDS-polyacrylamide gels and transferred onto polyvinylidene difluoride (PVDF) membranes (Millipore, Temecula, CA). The transferred membranes were blocked with 10% dry milk and sequentially incubated with primary antibodies and horseradish peroxidase (HRP)-conjugated second antibodies. The immunoreactive bands were identified using a chemiluminescent HRP substrate kit (Millipore). Green fluorescent protein (GFP) was used as an internal control.

To further examine the interaction of the mutated monomers of the ALDH1A3 proteins, co-immunoprecipitation (Co-IP) analyses were performed according to the manufacturer's instructions. Briefly, the extracted proteins were incubated with 3 μg of HA antibody (Santa Cruz, Dallas, TX) per sample. Then, protein A+G agarose beads (Beyotime, Shanghai, China) were added to each incubation sample. The bound proteins were isolated by centrifugation and purified with PBS. Finally, the Co-IP proteins were further detected by IB analysis with anti-FLAG antibodies.

## Results

Exome sequencing detected 36,195 variants presented in the affected boy (Tables S1 and S2). Among these variants, 489 non-synonymous and frame-shifted variants were predicted to be damaging and potentially pathogenic (Table S3). In this study, the mutations located in the *MFRP* and *ALDH1A3* genes were examined primarily because both candidate genes had been reported to cause A/M in a monogenetic manner.

First, novel biallelic heterozygous mutations at the sites c.287G > A (Genome position: chr15:101427859) in exon 3 and c.709G > A (Genome position: chr15:101436180) in exon 7 of the *ALDH1A3* gene were detected in the affected boy (Figure 2B), resulting in two missense mutations of p.(Arg96His) and p.(Gly237Arg). Further sequencing verified that his father is heterozygous in c.287G > A and his mother is heterozygous in c.709G > A of the *ALDH1A3* gene (Figure 2B). These two mutations in the *ALDH1A3* genes have not previously been reported, and both mutations were absent in all of the searched databases, including the 10,000 Chinese exome database.

The following alignments of the related amino acid sequences in a variety of species using Clustal Omega (http://www.ebi.ac.uk/Tools/msa/clustalo/) revealed that the two amino acid residues Arg96 and Gly237 were highly conserved not only in ALDH1A3 orthologs but also in the two paralogs of ALDH1A1 and ALDH1A2 (Figure 2C). Both ALDH1A3 missense mutations were predicted to be damaging using SIFT (score: 0.00 and 0.00, respectively) and Polyphen-2 (score: 1.00 and 0.99, respectively). Three-dimensional structure modeling of the tetrameric human ALDH1A3 protein was performed using SWISS-MODEL (http://swissmodel.expasy.org/) software with sheep liver cytosolic aldehyde dehydrogenase (PDB entry 1BXS, with 71.26% sequence identity to human ALDH1A3) as a template. The structure illustrated that the arginine (R) at position 96 resided next to the alpha-alpha helix corner, near the subunit contact sites of the ALDH1A3 homo-tetramer (Figure S1A) The glycine (G) at position 237 coiled the beta sheet and alpha helix near the end of the N-terminal domain (Figure S1B). Compared with the protein template PDB 1BXS, the corresponding residue R96 was located inside the nicotinamide adenine dinucelotide (NAD) binding pocket, and G237 was located at the ligand NAD contacting sites (Figure S1B, C). Thus, we deduced that the replacement of the R96 by a histidine (H) may alter the complementary interface among the monomers and then change the conformation of the tetramer, and that the substitution of G237 for the sterically hindered arginine could disrupt NAD binding.

**Figure 3:**
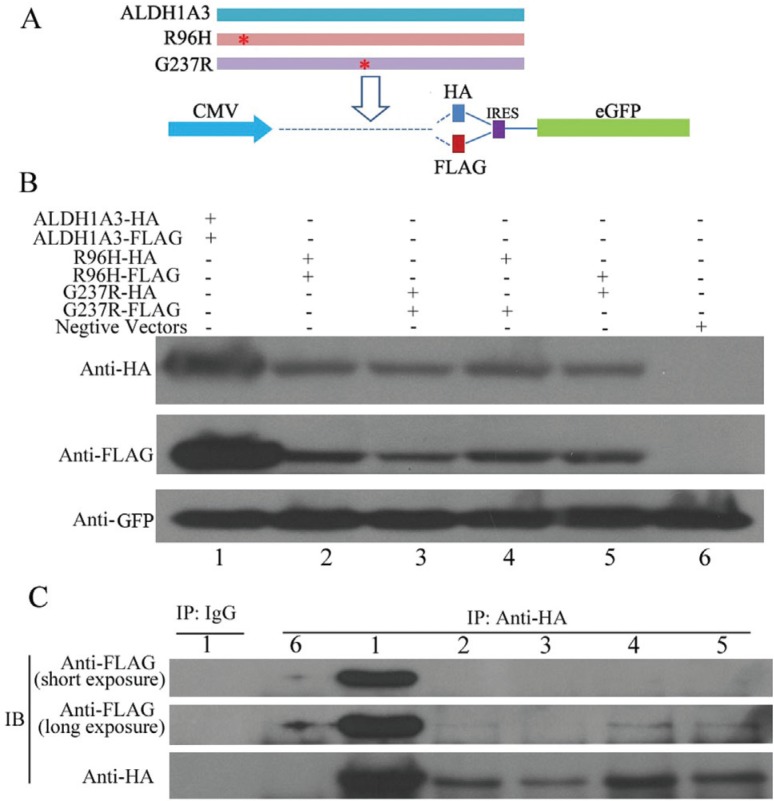
*In vitro* expression of the wild–type ALDH1A3 and two mutant R96H and G237R proteins. A. Scheme for constructing expression vectors for the wild–type ALDH1A3 and two mutant R96H and G237R proteins. B. Expression levels of the wild–type and two mutant ALDH1A3 proteins were examined by the immunoblot analysis with anti-HA and anti-FLAG antibodies. GFP was used as an internal control. The numbers from 1 to 6 indicate the six groups of protein products expressed in 293T cells transfected with different plasmid constructs. B. Interaction of the wild–type and two mutant ALDH1A3 proteins was analyzed by Co-IP analysis with the anti-HA antibody and IB analysis with an anti-FLAG antibody. The numbers from 1 to 6 indicate the different protein products obtained from A. A long exposure shows that the proteins R96H-FLAG and G237R-FlAG can be slightly immunoprecipitated by R96H-HA and G237R-HA.

To further examine the potential deleterious effects of the two mutant ALDH1A3 proteins of R96H and G237R, the recombinant wild-type (WT) and two mutant R96H and G237R proteins (Figure 3A) were transiently expressed in the 293T cells. The qRT-PCR analysis showed no significant difference in the mRNA expression levels among WT ALDH1A3, R96H and G237R (Figure S2), whereas the IB analysis of the extracted proteins demonstrated that both R96H and G237R proteins were much less expressed than the WT ALDH1A3 proteins (Figure 3B). Additionally, the subsequent Co-IP analysis showed that the two mutant proteins exhibited diminished binding to each other (Figure 3C). These results indicated that the missense mutations indeed damage the interaction of mutant ALDH1A3 monomers.

The boy also harbored a homozygous minor variation of g.119346557 C>T (rs79836575) located at the 5'untranslated region (UTR) of the first exon in the *MFRP* gene. Although the rs79836575 minor allele (T) frequency is rare in Western populations (< 0.01), this mutation is observed with a much higher frequency of 0.033 in Southern Han Chinese (http://www.ncbi.nlm.nih.gov/variation/tools/1000genomes/?q=rs79836575). We validated the sequences in both parents and found that the father also had a homozygote TT and the mother a heterozygote CT at this site. Therefore, we excluded the correlation between this UTR variation and anophthalmia in this family.

## Discussion

In this study, we showed compound heterozygous mutations of c.287G > A and c.709G > A in the *ALDH1A3* gene in a newborn boy with anophthalmia, and the expression of two mutant proteins was significantly decreased *in vitro.* ALDH1A3 is a critical dehydrogenase that contributes to the conversion of retinaldehyde to retinoic acid, which is vital in the normal morphogenesis of eye development. The ALDH1A3 deficiencies were identified as a direct link between retinoic acid synthesis dysfunction and early eye development malformations in humans (Duester, 2009; Fuhrmann, 2010). The functional analysis in this study indicated that the compound heterozygous *ALDH1A3* genetic variants may result in the deficiency of ALDH1A3 function during eye development. These results provided a different insight into the pathogenic roles of new variants.

In addition, we observed that the reduced productions of the two mutant proteins were not caused by a decline in *ALDH1A3* mRNAs because none of the transient transcripts showed any differences in their mRNA levels*.* We proposed that the two mutant R96H and G237R proteins might be unstable and might thus be subjected to proteasomal degradation after synthesis in the cells. This hypothesis was supported by results of the *in silico* analysis and Co-IP. However, human tissues and samples were unfortunately unavailable to investigate the mutant *ALDH1A3* gene expression *in vivo* because *ALDH1A3* expression is primarily present in the salivary gland and prostate, according to the GTEx database (http://www.gtexportal.org). In fact, to examine the effects of the loss-function of the orthologous Aldh1a3 in animals, Yahyavi *et al.* (2013) constructed a zebrafish model with an Aldh1a3 deficiency using antisense morpholinos targeting the intron 2 and exon 2 boundary of the Aldh1a3 gene. These authors observed that the mutant embryos showed a significant reduction in eye size, delayed closure of the optic fissure and coloboma-like lesions. Hence, to further investigate the function of both mutations, animal models of mouse or zebrafish expressing the mutant R96H and G237R proteins will be helpful in future studies.

The results of the present study revealed that the transmission of compound heterozygous mutations in *ALDH1A3* from non-consanguineous parents can lead to A/M, but all previous studies have suggested that homozygous mutations in *ALDH1A3* confer autosomal recessive A/M in consanguineous families (Fares-Taie *et al.*, 2013; Aldahmesh *et al.*, 2013; Yahyavi *et al.*, 2013; Abouzeid *et al.*, 2014; Mory *et al.*, 2014; Roos *et al.*, 2014; Semerci *et al.*, 2014; Plaisancié, *et al.*, 2016). Compound heterozygous mutations are common causes of autosomal recessive inherited eye diseases. For example, biallelic heterozygous mutations of *DRAM2* and *TTLL5* lead to retinal dystrophies (El-Asrag *et al.*, 2015; Sergouniotis *et al.*, 2014), compound heterozygous mutations of *ATF6* are the basis of the cone dysfunction disorder achromatopsia (Kohl *et al.*, 2015), and compound heterozygous mutations in *OTX2* and *MAB21L2* were reported to be a cause of A/M (Ragge *et al.*, 2005; Rainger *et al.*, 2014). Here, these data suggest that the heterozygous mutations of *ALDH1A3* contribute to A/M.

In conclusion, we revealed novel compound heterozygous mutations (c.287G>A and c.709G>A) in the *ALDH1A3* gene in a newborn with anophthalmia in a non-consanguineous Chinese family. The functional analysis confirmed that these mutations could result in impaired protein production. Thus, we propose that compound heterozygous variants of *ALDH1A3* should be considered for genetic screening in A/M cases, particularly in patients from common non-consanguineous families.

## Supplementary material

The following online material is available for this article:

Table S1 - Single nucleotide polymorphisms (SNPs) identified in the proband.

Table S2 - Insertion/Deletion (INDEL) mutations identified in the proband.

Table S3 - List of 489 non-synonymous and frame-shifting variants.

Figure S1 - Modeling of the three-dimensional structure of ALDH1A3 protein.

Figure S2 - Relative WT *ALDH1A3* and mutant *R96H* and *G237R* expression levels in 293T cell.
