# Supplementary material for: Novel compound heterozygous mutations of ALDH1A3 contribute to anophthalmia in a non-consanguineous Chinese family
Source: Genet Mol Biol. 2017 Jun 5;40(2):430–5. doi: 10.1590/1678-4685-GMB-2016-0120 (PMC5488456; doi:10.1590/1678-4685-GMB-2016-0120)
Supplement: Supplementary file 1 [file 1415-4757-gmb-1678-4685-GMB-2016-0120-Suppl01.pdf]

Table S1 - Single nucleotide polymorphisms (SNPs) identified in the proband.

Sample	Non-synonymous mutations	Synonymous mutations	Splice site mutations	UTR	Intron	Total
**Proband**	10835	11111	1780	9983	696	34405
