# Supplementary material for: Novel compound heterozygous mutations of ALDH1A3 contribute to anophthalmia in a non-consanguineous Chinese family
Source: Genet Mol Biol. 2017 Jun 5;40(2):430–5. doi: 10.1590/1678-4685-GMB-2016-0120 (PMC5488456; doi:10.1590/1678-4685-GMB-2016-0120)
Supplement: Supplementary file 3 [file 1415-4757-gmb-1678-4685-GMB-2016-0120-Suppl03.pdf]

Table S3 - List of 489 non-synonymous and frame-shifting variants which were predicted to be damaging (X: termination codon).

Gene	Uniprot function	Genome position (hg19)	AA position & change	SIFT	Polyphen-2
ALDH1A3	Recognizes as substrates free retinal and cellular retinol-binding protein-bound retinal. Seems to be the key enzyme in the formation of an RA gradient along the dorso-ventral axis during the early eye development and also in the development of the olfactory system.	chr15:101427859,c.287G>A(E3)	p.96,R>H	0	1
		chr15:101436180,c.709G>A(E7)	p.237,G>R	0	0.99
IRAK3	Interleukin-1 receptor-associated kinase 3, Inhibits dissociation of IRAK1 and IRAK4 from the Toll-like receptor signaling complex by either inhibiting the phosphorylation of IRAK1 and IRAK4 or stabilizing the receptor complex.	chr12:66605329,c.357C>A(E4),	p.119, Y>X (417)	_	_
PRKDC	Serine/threonine-protein kinase that acts as a molecular sensor for DNA damage. Involved in DNA non-homologous end joining (NHEJ) required for double-strand break (DSB) repair and V(D)J recombination. Must be bound to DNA to express its catalytic properties. Promotes processing of hairpin DNA structures in V(D)J recombination by activation of the hairpin endonuclease artemis (DCLRE1C). The assembly of the DNA-PK complex at DNA ends is also required for the NHEJ ligation step. Required to protect and align broken ends of DNA. May also act as a scaffold protein to aid the localization of DNA repair proteins to the site of damage. Found at the ends of chromosomes, suggesting a further role in the maintenance of telomeric stability and the prevention of chromosomal end fusion. Also involved in modulation of transcription. Recognizes the substrate consensus sequence [ST]-Q. Phosphorylates 'Ser-139' of histone variant H2AX/H2AFX, thereby regulating DNA damage response mechanism. Phosphorylates DCLRE1C, c-Abl/ABL1, histone H1, HSPCA, c-	chr8:48697705,c.11075C>T(E77),	p.3692, W>X(406)	_	_

Table S3 - List of 489 non-synonymous and frame-shifting variants which were predicted to be damaging (X: termination codon).

Gene	Uniprot function	Genome position (hg19)	AA position & change	SIFT	Polyphen-2
	jun/JUN, p53/TP53, PARP1, POU2F1, DHX9, SRF, XRCC1, XRCC1, XRCC4, XRCC5, XRCC6, WRN, MYC and RFA2. Can phosphorylate C1D not only in the presence of linear DNA but also in the presence of supercoiled DNA. Ability to phosphorylate p53/TP53 in the presence of supercoiled DNA is dependent on C1D. Contributes to the determination of the circadian period length by antagonizing phosphorylation of CRY1 'Ser-588' and increasing CRY1 protein stability, most likely through an indirect machanism. Interacts with CRY1 and CRY2; negatively regulates CRY1 phosphorylation.				
SCN2A	Sodium channel protein type 2 subunit alpha, Mediates the voltage-dependent sodium ion permeability of excitable membranes. Assuming opened or closed conformations in response to the voltage difference across the membrane, the protein forms a sodium-selective channel through which Na+ ions may pass in accordance with their electrochemical gradient.	chr2:166245784 c.5468A>C(E27)	p.1823,D>A	0.35	0.001
C4A	Complement C4-A,Non-enzymatic component of C3 and C5 convertases and thus essential for the propagation of the classical complement pathway. Covalently binds to immunoglobulins and immune complexes and enhances the solubilization of immune aggregates and the clearance of IC through CR1 on erythrocytes. C4A isotype is responsible for effective binding to form amide bonds with immune aggregates or protein antigens, while C4B isotype catalyzes the transacylation of the thioester carbonyl group to form ester bonds with carbohydrate antigens. Derived from proteolytic degradation of complement C4, C4a anaphylatoxin is a mediator	chr6:31964321 c.3620T>C(E28)	p.1207,V>A	0.14	0

Table S3 - List of 489 non-synonymous and frame-shifting variants which were predicted to be damaging (X: termination codon).

Gene	Uniprot function	Genome position (hg19)	AA position & change	SIFT	Polyphen-2
	of local inflammatory process. It induces the contraction of smooth muscle, increases vascular permeability and causes histamine release from mast cells and basophilic leukocytes.				
DNAH11	Dynein heavy chain 11, axonemal, Force generating protein of respiratory cilia. Produces force towards the minus ends of microtubules. Dynein has ATPase activity; the force-producing power stroke is thought to occur on release of ADP.	chr7:21658769 c.4321C>T(E24)	p.1441,R>W	_	0.655
EIF2AK4	eIF-2-alpha kinase GCN2, Metabolic-stress sensing protein kinase that phosphorylates the alpha subunit of eukaryotic translation initiation factor 2 (eIF-2-alpha/EIF2S1) on 'Ser-52' in response to low amino acid availability (PubMed:25329545). Plays a role as an activator of the integrated stress response (ISR) required for adapatation to amino acid starvation. Converts phosphorylated eIF-2-alpha/EIF2S1 either to a competitive inhibitor of the translation initiation factor eIF-2B, leading to a global protein synthesis repression, and thus to a reduced overall utilization of amino acids, or to a translational initiation activation of specific mRNAs, such as the transcriptional activator ATF4, and hence allowing ATF4-mediated reprogramming of amino acid biosynthetic gene expression to alleviate nutrient depletion. Binds uncharged tRNAs (By similarity). Involved in cell cycle arrest by promoting cyclin D1 mRNA translation repression after the unfolded protein response pathway (UPR) activation or cell cycle inhibitor CDKN1A/p21 mRNA translation activation in response to amino acid deprivation (PubMed:26102367). Plays a role in the consolidation of synaptic plasticity, learning as well as formation of long-term memory. Plays a role in neurite outgrowth inhibition. Plays a	chr15:40284406 c.2662G>C(E17)	p.888,D>H	0.28	0.33

Table S3 - List of 489 non-synonymous and frame-shifting variants which were predicted to be damaging (X: termination codon).

Gene	Uniprot function	Genome position (hg19)	AA position & change	SIFT	Polyphen-2
	proapoptotic role in response to glucose deprivation. Promotes global cellular protein synthesis repression in response to UV irradiation independently of the stress-activated protein kinase/c-Jun N-terminal kinase (SAPK/JNK) and p38 MAPK signaling pathways (By similarity). Plays a role in the antiviral response against alphavirus infection; impairs early viral mRNA translation of the incoming genomic virus RNA, thus preventing alphavirus replication (By similarity).				
CCDC164	Dynein regulatory complex protein 1, Key component of the nexin-dynein regulatory complex (N-DRC), essential for N-DRC integrity. Required for the assembly and regulation of specific classes of inner dynein arm motors. May also function to restrict dynein-driven microtubule sliding, thus aiding in the generation of ciliary bending.	chr2:26679362 c.2200G>A(E17)	p.734,V>M	0.1	0.412
NPHP4	Nephrocystin-4, Involved in the organization of apical junctions in kidney cells together with NPHP1 and RPGRIP1L/NPHP8. Does not seem to be strictly required for ciliogenesis.	chr1:5987742 c.1408G>A(E11)	p.470,R>W	0.09	0.742
KLKB1	Plasma kallikrein,The enzyme cleaves Lys-Arg and Arg-Ser bonds. It activates, in a reciprocal reaction, factor XII after its binding to a negatively charged surface. It also releases bradykinin from HMW kininogen and may also play a role in the renin-angiotensin system by converting prorenin into renin.	chr4:187171427 c.629C>A(E7)	p.210,A>E	0.57	0.1
SEPT-12	Septin-12, Filament-forming cytoskeletal GTPase (By similarity). May play a role in cytokinesis (Potential).	chr16:4837574 c.73C>T(E2)	p.25,E>K	0.1	0.019
MTMR2	Myotubularin-related protein 2, Phosphatase that acts on lipids with a phosphoinositol headgroup. Has phosphatase activity towards	chr11:95568581 c.1589G>C(E18)	p.530,A>G	0.19	0.171

Table S3 - List of 489 non-synonymous and frame-shifting variants which were predicted to be damaging (X: termination codon).

Gene	Uniprot function	Genome position (hg19)	AA position & change	SIFT	Polyphen-2
	phosphatidylinositol 3-phosphate and phosphatidylinositol 3,5-bisphosphate.				
SYNE1	Nesprin-1,Multi-isomeric modular protein which forms a linking network between organelles and the actin cytoskeleton to maintain the subcellular spatial organization. Component of SUN-protein-containing multivariate complexes also called LINC complexes which link the nucleoskeleton and cytoskeleton by providing versatile outer nuclear membrane attachment sites for cytoskeletal filaments. May be involved in the maintenance of nuclear organization and structural integrity. Connects nuclei to the cytoskeleton by interacting with the nuclear envelope and with F-actin in the cytoplasm. May be required for centrosome migration to the apical cell surface during early ciliogenesis.	chr6:152652051 c.13556T>C(E77)	p.4519,N>S	_	0.919
WDR35	WD repeat-containing protein 35, Component of the IFT complex A (IFT-A), a complex required for retrograde ciliary transport. Required for ciliogenesis. May promote CASP3 activation and TNF-stimulated apoptosis.	chr2:20166621 c.1058C>G(E10)	p.353,R>P	0.01	0.365
VKORC1	Vitamin K epoxide reductase complex subunit 1,nvolved in vitamin K metabolism. Catalytic subunit of the vitamin K epoxide reductase (VKOR) complex which reduces inactive vitamin K 2,3-epoxide to active vitamin K. Vitamin K is required for the gamma-carboxylation of various proteins, including clotting factors, and is required for normal blood coagulation, but also for normal bone development.	chr16:31104713 c.203T>C(E2)	p.68,H>R	0.37	0.03
DPYD	Dihydropyrimidine dehydrogenase [NADP(+)], Involved in pyrimidine base degradation. Catalyzes the reduction of uracil and thymine. Also involved the degradation of the chemotherapeutic drug 5-fluorouracil.	chr1:97700547 c.2303G>T(E19)	p.768,T>K	0.16	0.046

Table S3 - List of 489 non-synonymous and frame-shifting variants which were predicted to be damaging (X: termination codon).

Gene	Uniprot function	Genome position (hg19)	AA position & change	SIFT	Polyphen-2
USH2A	Usherin,Involved in hearing and vision.	chr1:216172258 c.6628G>C(E34)	p.2210,P>A	_	0.98
CAMTA	Calmodulin-binding transcription activator 1, Transcriptional activator. May act as a tumor suppressor.	chr1:7723593 c.986A>G(E9)	p.329,K>R	0.5	0.991
MRAP	Alpha-2-macroglobulin receptor-associated protein, Interacts with LRP1/alpha-2-macroglobulin receptor and glycoprotein 330.	chr21:33684212 c.424C>G(E5)	p.142,L>V	0.15	0.482
CARD14	Caspase recruitment domain-containing protein 14, Plays a role in signaling mediated by TRAF2, TRAF3 and TRAF6 and protects cells against apoptosis. Activates NF-kappa-B via BCL10 and IKK. Stimulates the phosphorylation of BCL10.	chr17:78157888 c.526G>C(E4)	p.176,D>H	0	0.867
RNASEL	2-5A-dependent ribonuclease, Endoribonuclease that functions in the interferon (IFN) antiviral response. In INF treated and virus infected cells, RNASEL probably mediates its antiviral effects through a combination of direct cleavage of single-stranded viral RNAs, inhibition of protein synthesis through the degradation of rRNA, induction of apoptosis, and induction of other antiviral genes. RNASEL mediated apoptosis is the result of a JNK-dependent stress-response pathway leading to cytochrome c release from mitochondria and caspase-dependent apoptosis. Therefore, activation of RNASEL could lead to elimination of virus infected cells under some circumstances. In the crosstalk between autophagy and apoptosis proposed to induce autophagy as an early stress response to small double-stranded RNA and at later stages of prolonged stress to activate caspase-dependent proteolytic cleavage of BECN1 to terminate autophagy and promote apoptosis (PubMed:26263979). Might play a central role in the regulation of mRNA turnover (PubMed:11585831).	chr1:182550490 c.1775C>T(E5)	p.592,R>H	0	0.993

Table S3 - List of 489 non-synonymous and frame-shifting variants which were predicted to be damaging (X: termination codon).

Gene	Uniprot function	Genome position (hg19)	AA position & change	SIFT	Polyphen-2
LRRK2	Leucine-rich repeat serine/threonine-protein kinase 2, Positively regulates autophagy through a calcium-dependent activation of the CaMKK/AMPK signaling pathway. The process involves activation of nicotinic acid adenine dinucleotide phosphate (NAADP) receptors, increase in lysosomal pH, and calcium release from lysosomes. Together with RAB29, plays a role in the retrograde trafficking pathway for recycling proteins, such as mannose 6 phosphate receptor (M6PR), between lysosomes and the Golgi apparatus in a retromer-dependent manner. Regulates neuronal process morphology in the intact central nervous system (CNS). Plays a role in synaptic vesicle trafficking. Phosphorylates PRDX3. Has GTPase activity. May play a role in the phosphorylation of proteins central to Parkinson disease.	chr12:40646786 c.1256C>T(E11)	p.419,A>V	0.02	0.817
COL6A3	Collagen alpha-3(VI) chain, Collagen VI acts as a cell-binding protein.	chr2:238283448 c.3286G>A(E8)	p.1096,R>C	_	0.99
ABCA1	ATP-binding cassette sub-family A member 1, cAMP-dependent and sulfonylurea-sensitive anion transporter. Key gatekeeper influencing intracellular cholesterol transport.	chr9:107576482 c.3818C>T(E27)	p.1273,R>Q	0.35	0.379
PCYT1A	Choline-phosphate cytidylyltransferase A, Controls phosphatidylcholine synthesis.	chr3:195997345 c.58C>G(E3)	p.20,G>R	0.1	0.015
FGD4	FYVE, RhoGEF and PH domain-containing protein 4, Activates CDC42, a member of the Ras-like family of Rho- and Rac proteins, by exchanging bound GDP for free GTP. Plays a role in regulating the actin cytoskeleton and cell shape. Activates MAPK8 (By similarity).	chr12:32735175 c.374C>T(E4)	p.125,T>M	0.11	0.049
IFT140	Intraflagellar transport protein 140 homolog, Component of the IFT complex A (IFT-A), a complex required for retrograde ciliary transport. Plays a pivotal role in proper development and	chr16:1569934 c.3988C>T(E29)	p.1330,A>T	0.28	0.08

Table S3 - List of 489 non-synonymous and frame-shifting variants which were predicted to be damaging (X: termination codon).

Gene	Uniprot function	Genome position (hg19)	AA position & change	SIFT	Polyphen-2
	function of ciliated cells. Involved in ciliogenesis and cilia maintenance.				
FSCN2	Fascin-2, Acts as an actin bundling protein. May play a pivotal role in photoreceptor cell-specific events, such as disk morphogenesis.	chr17:79495808 c.251C>T(E1)	p.84,P>L	0	0.948
TNFRSF	Tumor necrosis factor receptor superfamily member 13B, Receptor for TNFSF13/APRIL and TNFSF13B/TALL1/BAFF/BLYS that binds both ligands with similar high affinity. Mediates calcineurin-dependent activation of NF-AT, as well as activation of NF-kappa-B and AP-1. Involved in the stimulation of B- and T-cell function and the regulation of humoral immunity.	chr17:16843027 c.716G>A(E5)	p.239,A>V	0.12	0.026
LOXHD1	Lipoxygenase homology domain-containing protein 1, Involved in hearing. Required for normal function of hair cells in the inner ear (By similarity).	chr18:44157796 c.1844C>T(E14)	p.615,R>Q	0.01	0.776
PEX6	Peroxisomal ATPase PEX6, Component of the peroxisomal protein import machinery. Together with PEX1, mediates the ATP-dependent relocation and recycling of the peroxisomal targeting signal-1 (PTS1) import receptor PEX5 from the peroxisomal membrane to the cytosol, where it is then available for another round of protein import into the organelle.	chr6:42933047 c.2531A>G(E14)	p.844,V>A	0.02	0.651
HR	Lysine-specific demethylase hairless, Histone demethylase that specifically demethylates both mono- and dimethylated 'Lys-9' of histone H3. May act as a transcription regulator controlling hair biology (via targeting of collagens), neural activity, and cell cycle.	chr8:21980362 c.1946G>A(E7)	p.649,T>M	0.02	0.707
ACAD9	Acyl-CoA dehydrogenase family member 9, mitochondrial, Required for mitochondrial complex I assembly (PubMed:20816094, PubMed:24158852). Has a dehydrogenase activity	chr3:128622934 c.988A>C(E10)	p.330,K>Q	0.17	0.992

Table S3 - List of 489 non-synonymous and frame-shifting variants which were predicted to be damaging (X: termination codon).

Gene	Uniprot function	Genome position (hg19)	AA position & change	SIFT	Polyphen-2
	on palmitoyl-CoA (C16:0) and stearoyl-CoA (C18:0). It is three times more active on palmitoyl-CoA than on stearoyl-CoA. However, it does not play a primary role in long-chain fatty acid oxidation in vivo				
RTEL1	Regulator of telomere elongation helicase 1, ATP-dependent DNA helicase implicated in telomere-length regulation, DNA repair and the maintenance of genomic stability. Acts as an anti-recombinase to counteract toxic recombination and limit crossover during meiosis. Regulates meiotic recombination and crossover homeostasis by physically dissociating strand invasion events and thereby promotes noncrossover repair by meiotic synthesis dependent strand annealing (SDSA) as well as disassembly of D loop recombination intermediates. Also disassembles T loops and prevents telomere fragility by counteracting telomeric G4-DNA structures, which together ensure the dynamics and stability of the telomere.	chr20:62326159 c.3175G>A(E32)	p.1059,A>T	0.17	0.031
KIF1C	Kinesin-like protein KIF1C, Motor required for the retrograde transport of Golgi vesicles to the endoplasmic reticulum. Has a microtubule plus end-directed motility.	chr17:4927183 c.3049G>A(E23)	p.1017,A>T	0.36	0.005
FKBP5	Peptidyl-prolyl cis-trans isomerase FKBP5, Immunophilin protein with PPIase and co-chaperone activities. Component of unligated steroid receptors heterocomplexes through interaction with heat-shock protein 90 (HSP90). Plays a role in the intracellular trafficking of heterooligomeric forms of steroid hormone receptors maintaining the complex into the cytoplasm when unliganded.	chr6:35543677 c.1309C>A(E12)	p.437,V>F	0.28	0.001
CHD7	Chromodomain-helicase-DNA-binding protein 7, Probable transcription regulator. Maybe involved in the in 45S precursor rRNA production.	chr8:61655556 c.1565G>T(E2)	p.522,G>V	_	0.953

Table S3 - List of 489 non-synonymous and frame-shifting variants which were predicted to be damaging (X: termination codon).

Gene	Uniprot function	Genome position (hg19)	AA position & change	SIFT	Polyphen-2
GPSM2	G-protein-signaling modulator 2, Plays an important role in mitotic spindle pole organization via its interaction with NUMA1 (PubMed:15632202, PubMed:21816348). Plays an important role in asymmetric cell divisions (PubMed:21816348). Has guanine nucleotide dissociation inhibitor (GDI) activity towards G(i) alpha proteins, such as GNAI1 and GNAI3, and thereby regulates their activity (By similarity).	chr1:109465166-109465168,c.1568(E13)-c.1570(E13):deleted CTT	523-524 deletion	_	_
VPS35	Vacuolar protein sorting-associated protein 35, Acts as component of the retromer cargo-selective complex (CSC). The CSC is believed to be the core functional component of retromer or respective retromer complex variants acting to prevent missorting of selected transmembrane cargo proteins into the lysosomal degradation pathway. The recruitment of the CSC to the endosomal membrane involves RAB7A and SNX3. The CSC seems to associate with the cytoplasmic domain of cargo proteins predominantly via VPS35; however, these interactions seem to be of low affinity and retromer SNX proteins may also contribute to cargo selectivity thus questioning the classical function of the CSC. The SNX-BAR retromer mediates retrograde transport of cargo proteins from endosomes to the trans-Golgi network (TGN) and is involved in endosome-to-plasma membrane transport for cargo protein recycling. The SNX3-retromer mediates the retrograde endosome-to-TGN transport of WLS distinct from the SNX-BAR retromer pathway. The SNX27-retromer is believed to be involved in endosome-to-plasma membrane trafficking and recycling of a broad spectrum of cargo proteins. The CSC seems to act as recruitment hub for other proteins, such as the WASH complex and TBC1D5	chr16:46696246 c.1976T>C(E15)	p.659,K>R	0.05	0.422

Table S3 - List of 489 non-synonymous and frame-shifting variants which were predicted to be damaging (X: termination codon).

Gene	Uniprot function	Genome position (hg19)	AA position & change	SIFT	Polyphen-2
	(Probable). Required for retrograde transport of lysosomal enzyme receptor IGF2R and SLC11A2. Required to regulate transcytosis of the polymeric immunoglobulin receptor (pIgR-pIgA). Required for endosomal localization of FAM21C . Mediates the association of the CSC with the WASH complex via FAM21. Required for the endosomal localization of TBC1D5.				
SCNN1A	Amiloride-sensitive sodium channel subunit alpha, Sodium permeable non-voltage-sensitive ion channel inhibited by the diuretic amiloride. Mediates the electrodiffusion of the luminal sodium (and water, which follows osmotically) through the apical membrane of epithelial cells. Plays an essential role in electrolyte and blood pressure homeostasis, but also in airway surface liquid homeostasis, which is important for proper clearance of mucus. Controls the reabsorption of sodium in kidney, colon, lung and sweat glands. Also plays a role in taste perception.	chr12:6457101 c.1948G>A(E13)	p.650,R>C	0.2	0
JAK2	Tyrosine-protein kinase JAK2, Non-receptor tyrosine kinase involved in various processes such as cell growth, development, differentiation or histone modifications. Mediates essential signaling events in both innate and adaptive immunity. In the cytoplasm, plays a pivotal role in signal transduction via its association with type I receptors such as growth hormone (GHR), prolactin (PRLR), leptin (LEPR), erythropoietin (EPOR), thrombopoietin (THPO); or type II receptors including IFN-alpha, IFN-beta, IFN-gamma and multiple interleukins (PubMed:7615558). Following ligand-binding to cell surface receptors, phosphorylates specific tyrosine residues on the cytoplasmic tails of the receptor, creating docking sites for STATs proteins	chr9:5044432 c.380G>A(E5)	p.127,G>D	0.02	0.059

Table S3 - List of 489 non-synonymous and frame-shifting variants which were predicted to be damaging (X: termination codon).

Gene	Uniprot function	Genome position (hg19)	AA position & change	SIFT	Polyphen-2
	(PubMed:9618263). Subsequently, phosphorylates the STATs proteins once they are recruited to the receptor. Phosphorylated STATs then form homodimer or heterodimers and translocate to the nucleus to activate gene transcription. For example, cell stimulation with erythropoietin (EPO) during erythropoiesis leads to JAK2 autophosphorylation, activation, and its association with erythropoietin receptor (EPOR) that becomes phosphorylated in its cytoplasmic domain. Then, STAT5 (STAT5A or STAT5B) is recruited, phosphorylated and activated by JAK2. Once activated, dimerized STAT5 translocates into the nucleus and promotes the transcription of several essential genes involved in the modulation of erythropoiesis. In addition, JAK2 mediates angiotensin-2-induced ARHGEF1 phosphorylation (PubMed:20098430). Plays a role in cell cycle by phosphorylating CDKN1B. Cooperates with TEC through reciprocal phosphorylation to mediate cytokine-driven activation of FOS transcription. In the nucleus, plays a key role in chromatin by specifically mediating phosphorylation of 'Tyr-41' of histone H3 (H3Y41ph), a specific tag that promotes exclusion of CBX5 (HP1 alpha) from chromatin.				
SCN4A	Sodium channel protein type 4 subunit alpha, This protein mediates the voltage-dependent sodium ion permeability of excitable membranes. Assuming opened or closed conformations in response to the voltage difference across the membrane, the protein forms a sodium-selective channel through which Na+ ions may pass in accordance with their electrochemical gradient. This sodium channel may be present in both denervated and innervated skeletal muscle.	chr17:62018760 c.4882C>T(E24)	p.1628,D>N	0	0.865

Table S3 - List of 489 non-synonymous and frame-shifting variants which were predicted to be damaging (X: termination codon).

Gene	Uniprot function	Genome position (hg19)	AA position & change	SIFT	Polyphen-2
EIF2B4	Translation initiation factor eIF-2B subunit delta, Catalyzes the exchange of eukaryotic initiation factor 2-bound GDP for GTP.	chr2:27590038 c.916G>C(E10)	p.306,R>G	0.36	0.002
LAMC3	Laminin subunit gamma-3, Binding to cells via a high affinity receptor, laminin is thought to mediate the attachment, migration and organization of cells into tissues during embryonic development by interacting with other extracellular matrix components.	chr9:133951317 c.3594G>C(E21)	p.1198,R>S	0.26	0.006
MLLT10	Protein AF-10, Probably involved in transcriptional regulation. In vitro or as fusion protein with KMT2A/MLL1 has transactivation activity. Binds to cruciform DNA.	chr10:22015191 c.1897T>A(E14)	p.633,S>T	0.03	0.078
CC2D2A	Coiled-coil and C2 domain-containing protein 2A, Component of the tectonic-like complex, a complex localized at the transition zone of primary cilia and acting as a barrier that prevents diffusion of transmembrane proteins between the cilia and plasma membranes. Required for ciliogenesis and sonic hedgehog/SHH signaling (By similarity).	chr4:15529188 c.1268G>A(E13)	p.423,R>Q	0.21	0.897
GIGYF2	May act cooperatively with GRB10 to regulate tyrosine kinase receptor signaling, including IGF1 and insulin receptors.	chr2:233712232, c.3635(E27)-c.3636(E27): in	1210-1212 QQ insertion	_	_
LAMB2	Laminin subunit beta-2, Binding to cells via a high affinity receptor, laminin is thought to mediate the attachment, migration and organization of cells into tissues during embryonic development by interacting with other extracellular matrix components.	chr3:49161830 c.3325C>T(E22)	p.1109,E>K	0.15	0.487
EPHA2	Ephrin type-A receptor 2, Receptor tyrosine kinase which binds promiscuously membrane-bound ephrin-A family ligands residing on adjacent cells, leading to contact-dependent bidirectional signaling into neighboring cells. The signaling pathway downstream of the receptor is referred to as forward signaling while the signaling pathway	chr1:16464614 c.1046G>A(E5)	p.349,T>M	0.07	0.017

Table S3 - List of 489 non-synonymous and frame-shifting variants which were predicted to be damaging (X: termination codon).

Gene	Uniprot function	Genome position (hg19)	AA position & change	SIFT	Polyphen-2
	downstream of the ephrin ligand is referred to as reverse signaling. Activated by the ligand ephrin-A1/EFNA1 regulates migration, integrin-mediated adhesion, proliferation and differentiation of cells. Regulates cell adhesion and differentiation through DSG1/desmoglein-1 and inhibition of the ERK1/ERK2 (MAPK3/MAPK1, respectively) signaling pathway. May also participate in UV radiation-induced apoptosis and have a ligand-independent stimulatory effect on chemotactic cell migration. During development, may function in distinctive aspects of pattern formation and subsequently in development of several fetal tissues. Involved for instance in angiogenesis, in early hindbrain development and epithelial proliferation and branching morphogenesis during mammary gland development. Engaged by the ligand ephrin-A5/EFNA5 may regulate lens fiber cells shape and interactions and be important for lens transparency development and maintenance. With ephrin-A2/EFNA2 may play a role in bone remodeling through regulation of osteoclastogenesis and osteoblastogenesis				
LAMA2	Laminin subunit alpha-2, Binding to cells via a high affinity receptor, laminin is thought to mediate the attachment, migration and organization of cells into tissues during embryonic development by interacting with other extracellular matrix components.	chr6:129588259 c.2217G>T(E16)	p.739,W>C	0.06	0.926
LAMA1	Laminin subunit alpha-1, Binding to cells via a high affinity receptor, laminin is thought to mediate the attachment, migration and organization of cells into tissues during embryonic development by interacting with other extracellular matrix components.	chr18:6971872 c.6883G>A(E48)	p.2295,R>C	0.01	0.586

Table S3 - List of 489 non-synonymous and frame-shifting variants which were predicted to be damaging (X: termination codon).

Gene	Uniprot function	Genome position (hg19)	AA position & change	SIFT	Polyphen-2
ZFHX4	Zinc finger homeobox protein 4, May play a role in neural and muscle differentiation (By similarity). May be involved in transcriptional regulation.	chr8:77765999 c.6842C>T(E10)	p.2281,A>V	_	0.956
CHRNB1	Acetylcholine receptor subunit beta, After binding acetylcholine, the AChR responds by an extensive change in conformation that affects all subunits and leads to opening of an ion-conducting channel across the plasma membrane.	chr17:7359930 c.1394T>C(E11)	p.465,M>T	0.03	0.975
GYPB	Glycophorin-B, This protein is a minor sialoglycoprotein in erythrocyte membranes.	chr4:144920566 c.173G>C(E3)	p.58,P>R	0	0.341
MYBPC3	Myosin-binding protein C, cardiac-type, Thick filament-associated protein located in the crossbridge region of vertebrate striated muscle a bands. In vitro it binds MHC, F-actin and native thin filaments, and modifies the activity of actin-activated myosin ATPase. It may modulate muscle contraction or may play a more structural role.	chr11:47354781 c.3294C>G(E30)	p.1098,W>C	0.18	0.704
ADAM1	Disintegrin and metalloproteinase domain-containing protein 10,	chr15:58936150 c.763T>C(E7)	p.255,T>A	0.86	0.006
RIN2	E3 ubiquitin protein ligase RIN2, E3 ubiquitin protein ligase that acts as positive regulator of RPM1- and RPS2-dependent hypersensitive response (HR), in association with RIN3. Probably not required for RPM1 degradation during HR.	chr20:19981515 c.2770C>T(E12)	p.924,R>C	0	0.995
SGCD	Delta-sarcoglycan, Component of the sarcoglycan complex, a subcomplex of the dystrophin-glycoprotein complex which forms a link between the F-actin cytoskeleton and the extracellular matrix.	chr5:156184733 c.717C>G(E8)	p.239,D>E	0.27	0
TG	Thyroglobulin, Precursor of the iodinated thyroid hormones thyroxine (T4) and triiodothyronine (T3).	chr8:133909927 c.3035C>T(E12)	p.1012,P>L	0.21	0

Table S3 - List of 489 non-synonymous and frame-shifting variants which were predicted to be damaging (X: termination codon).

Gene	Uniprot function	Genome position (hg19)	AA position & change	SIFT	Polyphen-2
FAT4	Protocadherin Fat 4, Cadherins are calcium-dependent cell adhesion proteins. FAT4 plays a role in the maintenance of planar cell polarity as well as in inhibition of YAP1-mediated neuroprogenitor cell proliferation and differentiation (By similarity).	chr4:126373858 c.11687C>T(E9)	p.3896,A>V	_	0.958
DISC1	Disrupted in schizophrenia 1 protein, Involved in the regulation of multiple aspects of embryonic and adult neurogenesis. Required for neural progenitor proliferation in the ventrical/subventrical zone during embryonic brain development and in the adult dentate gyrus of the hippocampus. Participates in the Wnt-mediated neural progenitor proliferation as a positive regulator by modulating GSK3B activity and CTNNB1 abundance. Plays a role as a modulator of the AKT-mTOR signaling pathway controlling the tempo of the process of newborn neurons integration during adult neurogenesis, including neuron positioning, dendritic development and synapse formation. Inhibits the activation of AKT-mTOR signaling upon interaction with CCDC88A. Regulates the migration of early-born granule cell precursors toward the dentate gyrus during the hippocampal development. Plays a role, together with PCNT, in the microtubule network formation.	chr1:231906773 c.1591G>C(E6)	p.531,G>R	0.28	0.047
AP5Z1	AP-5 complex subunit zeta-1, As part of AP-5, a probable fifth adaptor protein complex it may be involved in endosomal transport. According to PubMed:20613862 it is a putative helicase required for efficient homologous recombination DNA double-strand break repair.	chr7:4827897 c.1567C>T(E12)	p.523,R>C	0	0.999
TTLL12		chr10:96084326 IVS30+2T>G	splicing site		

Table S3 - List of 489 non-synonymous and frame-shifting variants which were predicted to be damaging (X: termination codon).

Gene	Uniprot function	Genome position (hg19)	AA position & change	SIFT	Polyphen-2
CSF1R	Macrophage colony-stimulating factor 1 receptor, Tyrosine-protein kinase that acts as cell-surface receptor for CSF1 and IL34 and plays an essential role in the regulation of survival, proliferation and differentiation of hematopoietic precursor cells, especially mononuclear phagocytes, such as macrophages and monocytes. Promotes the release of proinflammatory chemokines in response to IL34 and CSF1, and thereby plays an important role in innate immunity and in inflammatory processes. Plays an important role in the regulation of osteoclast proliferation and differentiation, the regulation of bone resorption, and is required for normal bone and tooth development. Required for normal male and female fertility, and for normal development of milk ducts and acinar structures in the mammary gland during pregnancy. Promotes reorganization of the actin cytoskeleton, regulates formation of membrane ruffles, cell adhesion and cell migration, and promotes cancer cell invasion. Activates several signaling pathways in response to ligand binding. Phosphorylates PIK3R1, PLCG2, GRB2, SLA2 and CBL. Activation of PLCG2 leads to the production of the cellular signaling molecules diacylglycerol and inositol 1,4,5-trisphosphate, that then lead to the activation of protein kinase C family members, especially PRKCD. Phosphorylation of PIK3R1, the regulatory subunit of phosphatidylinositol 3-kinase, leads to activation of the AKT1 signaling pathway. Activated CSF1R also mediates activation of the MAP kinases MAPK1/ERK2 and/or MAPK3/ERK1, and of the SRC family kinases SRC, FYN and YES1. Activated CSF1R transmits signals both via proteins that directly interact with phosphorylated tyrosine residues in	chr5:149456893 c.835C>T(E6)	p.279,V>M	0.09	0.033

Table S3 - List of 489 non-synonymous and frame-shifting variants which were predicted to be damaging (X: termination codon).

Gene	Uniprot function	Genome position (hg19)	AA position & change	SIFT	Polyphen-2
	its intracellular domain, or via adapter proteins, such as GRB2. Promotes activation of STAT family members STAT3, STAT5A and/or STAT5B. Promotes tyrosine phosphorylation of SHC1 and INPP5D/SHIP-1. Receptor signaling is down-regulated by protein phosphatases, such as INPP5D/SHIP-1, that dephosphorylate the receptor and its downstream effectors, and by rapid internalization of the activated receptor				
EPHX1	Epoxide hydrolase 1, Biotransformation enzyme that catalyzes the hydrolysis of arene and aliphatic epoxides to less reactive and more water soluble dihydrodiols by the trans addition of water.	chr1:226027630 c.823A>G(E6)	p.275,T>A	0.15	0.232
HEATR2	Dynein assembly factor 5, axonemal, Cytoplasmic protein involved in the delivery of the dynein machinery to the motile cilium. It is required for the assembly of the axonemal dynein inner and outer arms, two structures attached to the peripheral outer doublet A microtubule of the axoneme, that play a crucial role in cilium motility.	chr7:796486 c.1325C>T(E6)	p.442,S>L	0.14	0.11
FLG	Fibroblast growth factor receptor 1, Tyrosine-protein kinase that acts as cell-surface receptor for fibroblast growth factors and plays an essential role in the regulation of embryonic development, cell proliferation, differentiation and migration. Required for normal mesoderm patterning and correct axial organization during embryonic development, normal skeletogenesis and normal development of the gonadotropin-releasing hormone (GnRH) neuronal system. Phosphorylates PLCG1, FRS2, GAB1 and SHB. Ligand binding leads to the activation of several signaling cascades. Activation of PLCG1 leads to the production of the cellular signaling molecules diacylglycerol and inositol 1,4,5-trisphosphate. Phosphorylation of FRS2 triggers recruitment of	chr1:152283079 c.4283C>T(E3)	p.1428,R>H	_	0.522
		chr1:152275298 c.12064T>A(E3)	p.4022,K>X(40)	_	_

Table S3 - List of 489 non-synonymous and frame-shifting variants which were predicted to be damaging (X: termination codon).

Gene	Uniprot function	Genome position (hg19)	AA position & change	SIFT	Polyphen-2
	GRB2, GAB1, PIK3R1 and SOS1, and mediates activation of RAS, MAPK1/ERK2, MAPK3/ERK1 and the MAP kinase signaling pathway, as well as of the AKT1 signaling pathway. Promotes phosphorylation of SHC1, STAT1 and PTPN11/SHP2. In the nucleus, enhances RPS6KA1 and CREB1 activity and contributes to the regulation of transcription. FGFR1 signaling is down-regulated by IL17RD/SEF, and by FGFR1 ubiquitination, internalization and degradation.				
SALL1	Sal-like protein 1, Transcriptional repressor involved in organogenesis.	chr16:51175324 c.518G>A(E2)	p.173,P>L	0.07	0.075
TBC1D20	TBC1 domain family member 20, GTPase-activating protein specific for Rab1 and Rab2 small GTPase families for which it can accelerate the intrinsic GTP hydrolysis rate by more than five orders of magnitude.	chr20:420894 c.766C>T(E6)	p.256,V>M	0.06	0.322
HPS4	Hermansky-Pudlak syndrome 4 protein, May function in the pathway of organelle biogenesis.	chr22:26853833 c.1947C>T(E13)	p.649,M>I	0.13	0.086
PLCB4	1-phosphatidylinositol 4,5-bisphosphate phosphodiesterase beta-4, The production of the second messenger molecules diacylglycerol (DAG) and inositol 1,4,5-trisphosphate (IP3) is mediated by activated phosphatidylinositol-specific phospholipase C enzymes. This form has a role in retina signal transduction.	chr20:9343621 c.448C>T(E5)	p.150,H>Y	0.13	0.274
MKKS	McKusick-Kaufman/Bardet-Biedl syndromes putative chaperonin, Probable molecular chaperone. Assists the folding of proteins upon ATP hydrolysis. As part of the BBS/CCT complex may play a role in the assembly of BBSome, a complex involved in ciliogenesis regulating transports vesicles to the cilia. May play a role in protein processing in limb, cardiac and reproductive system development. May play a role	chr20:10393747 c.416C>T(E3)	p.139,R>Q	0.02	0.861

Table S3 - List of 489 non-synonymous and frame-shifting variants which were predicted to be damaging (X: termination codon).

Gene	Uniprot function	Genome position (hg19)	AA position & change	SIFT	Polyphen-2
	in cytokinesis.				
GABRG2	Gamma-aminobutyric acid receptor subunit gamma-2, Component of the heteropentameric receptor for GABA, the major inhibitory neurotransmitter in the vertebrate brain. Functions also as histamine receptor and mediates cellular responses to histamine. Functions as receptor for diazepines and various anesthetics, such as pentobarbital; these are bound at a separate allosteric effector binding site. Functions as ligand-gated chloride channel.	chr5:161495057 c.52G>C(E1)	p.18,V>L	0.27	0.013
RTTN	Rotatin, Involved in the genetic cascade that governs left-right specification. Plays a role in the maintenance of a normal ciliary structure. Required for correct asymmetric expression of NODAL, LEFTY and PITX2.	chr18:67755238 c.4289A>G(E31)	p.1430,M>T	0	0.421
C5orf42	Uncharacterized protein C5orf42, cerebellum development, cilium assembly, coronary vasculature development, embryonic digit morphogenesis,planar polarity,kideney and palate development, protein localization to ciliary transition zone, vertricular septum development	chr5:37195983-37195994 c.3788(E21)-c.3777(E21): TCAAGCTTGTGG deleted	p.1259-1263 DHKLD/D	_	_
KRT3	Keratin, type II cytoskeletal 3, structural molecule activity, epithelial cell differentiation, intermediate filament cytoskeleton orgnaization	chr12:53189414-53189431 c.413(E1)-c.396(E1): CCAAA<8>CCCCT deleted	p.122-127 AGGFGG/_	_	_
PARK7	Protein deglycase DJ-1, Protein deglycase that repairs methylglyoxal- and glyoxal-glycated amino acids and proteins, and releases repaired proteins and lactate or glycolate, respectively. Deglycates cysteines, arginines and lysines residues in proteins, and thus reactivates these proteins by reversing glycation by glyoxals. Acts on early glycation intermediates (hemithioacetals and aminocarbinols), preventing the formation of advanced glycation endproducts (AGE) (PubMed:25416785). Plays an important role in	chr1:8037718 c.329C>G(E6)	p.110,T>S	0.02	0.384

Table S3 - List of 489 non-synonymous and frame-shifting variants which were predicted to be damaging (X: termination codon).

Gene	Uniprot function	Genome position (hg19)	AA position & change	SIFT	Polyphen-2
	cell protection against oxidative stress and cell death acting as oxidative stress sensor and redox-sensitive chaperone and protease; functions probably related to its primary function (PubMed:17015834, PubMed:20304780, PubMed:18711745, PubMed:12796482, PubMed:19229105, PubMed:25416785). It is involved in neuroprotective mechanisms like the stabilization of NFE2L2 and PINK1 proteins, male fertility as a positive regulator of androgen signaling pathway as well as cell growth and transformation through, for instance, the modulation of NF-kappa-B signaling pathway. Its involvement in protein repair could also explain other unrelated functions. Eliminates hydrogen peroxide and protects cells against hydrogen peroxide-induced cell death. Required for correct mitochondrial morphology and function as well as for autophagy of dysfunctional mitochondria. Plays a role in regulating expression or stability of the mitochondrial uncoupling proteins SLC25A14 and SLC25A27 in dopaminergic neurons of the substantia nigra pars compacta and attenuates the oxidative stress induced by calcium entry into the neurons via L-type channels during pacemaking. Regulates astrocyte inflammatory responses, may modulate lipid rafts-dependent endocytosis in astrocytes and neuronal cells. Binds to a number of mRNAs containing multiple copies of GG or CC motifs and partially inhibits their translation but dissociates following oxidative stress (PubMed:18626009). Metal-binding protein able to bind copper as well as toxic mercury ions, enhances the cell protection mechanism against induced metal toxicity				

Table S3 - List of 489 non-synonymous and frame-shifting variants which were predicted to be damaging (X: termination codon).

Gene	Uniprot function	Genome position (hg19)	AA position & change	SIFT	Polyphen-2
NAGA	Alpha-N-acetylgalactosaminidase, Removes terminal alpha-N-acetylgalactosamine residues from glycolipids and glycopeptides. Required for the breakdown of glycolipids.	chr22:42459035 IVS6-7G>T splicing cite	splicing region	_	_
DOCK6	Dedicator of cytokinesis protein 6, Acts as guanine nucleotide exchange factor (GEF) for CDC42 and RAC1 small GTPases. Through its activation of CDC42 and RAC1, may regulate neurite outgrowth (By similarity).	chr19:11352716 IVS14+5C>T splicing cite	splicing region	_	_
CYP4F22	Cytochrome P450 4F22, heme and iron ion binding, monooxygenase and oxidoreductase activity	chr19:15636317 c.170G>A(E3)	p.57,R>H	0.02	0.002
SRBD1	S1 RNA-binding domain-containing protein 1, RNA binding, nucleobase-containing compound metabolic process	chr2:45826656 c.580G>A(E4)	p.194,P>S	0.02	0.611
		chr2:45826667 c.569G>C(E4)	p.190,P>R	0.02	0.581
TEP1	Telomerase protein component 1, Component of the telomerase ribonucleoprotein complex that is essential for the replication of chromosome termini. Also component of the ribonucleoprotein vaults particle, a multi-subunit structure involved in nucleo-cytoplasmic transport. Responsible for the localizing and stabilizing vault RNA (vRNA) association in the vault ribonucleoprotein particle. Binds to TERC (By similarity).	chr14:20851708 c.3806T>C(E26)	p.1269,D>G	0	0.999
KRT76	Keratin, type II cytoskeletal 2 oral, Probably contributes to terminal cornification.	chr12:53164891 c.1376T>G(E7)	p.459,D>A	0.23	0.926
RUFY2	RUN and FYVE domain-containing protein 2, zinc finger, metal-binding	chr10:70105813 c.1733G>A(E17)	p.578,A>V	0.29	0.025
TUBGCP	Gamma-tubulin complex component 2, Gamma-tubulin complex is necessary for microtubule nucleation at the centrosome.	chr10:135113013 c.374G>A(E4)	p.125,A>V	0.27	0.004

Table S3 - List of 489 non-synonymous and frame-shifting variants which were predicted to be damaging (X: termination codon).

Gene	Uniprot function	Genome position (hg19)	AA position & change	SIFT	Polyphen-2
KRTAP9	Keratin-associated protein 9-8, In the hair cortex, hair keratin intermediate filaments are embedded in an interfilamentous matrix, consisting of hair keratin-associated proteins (KRTAP), which are essential for the formation of a rigid and resistant hair shaft through their extensive disulfide bond cross-linking with abundant cysteine residues of hair keratins. The matrix proteins include the high-sulfur and high-glycine-tyrosine keratins.	chr17:39394634 c.331G>A(E1)	p.111,V>I	0.03	0.936
CBY1	Protein chibby homolog 1, Inhibits the Wnt/Wingless pathway by binding to CTNNB1/beta-catenin and inhibiting beta-catenin-mediated transcriptional activation through competition with TCF/LEF transcription factors. Has also been shown to play a role in regulating the intracellular trafficking of polycystin-2/PKD2 and possibly of other intracellular proteins. Promotes adipocyte and cardiomyocyte differentiation.	chr22:39064088 c.158C>T(E3)	p.53,P>L	0.15	0.851
PLAC1	Placenta-specific protein 1, May play a role in placental development	chrX:133700486 c.227C>T(E3)	p.76,R>H	0.05	0.999
TMBIM6	Bax inhibitor 1, Suppressor of apoptosis (PubMed:21075086). Modulates unfolded protein response signaling (PubMed:21075086). Modulates ER calcium homeostasis by acting as a calcium-leak channel (PubMed:22128171). Negatively regulates autophagy and autophagosome formation, especially during periods of nutrient deprivation, and reduces cell survival during starvation (By similarity).	chr12:50146311 c.209C>G(E2)	p.70,A>G	0.01	0.859
ZNF705A	Zinc finger protein 705A, May be involved in transcriptional regulation.	chr12:8327883 c.199C>T(E3)	p.67,R>W	0	0.041
MUC21	Mucin-21, negative regulation of cell-cell and cell-substrate adhesion, O-glycan processing	chr6:30955211 c.1259C>T(E2)	p.420,A>V	0.32	0

Table S3 - List of 489 non-synonymous and frame-shifting variants which were predicted to be damaging (X: termination codon).

Gene	Uniprot function	Genome position (hg19)	AA position & change	SIFT	Polyphen-2
L3MBTL	Lethal(3)malignant brain tumor-like protein 2, Putative Polycomb group (PcG) protein. PcG proteins maintain the transcriptionally repressive state of genes, probably via a modification of chromatin, rendering it heritably changed in its expressibility. Its association with a chromatin-remodeling complex suggests that it may contribute to prevent expression of genes that trigger the cell into mitosis. Binds to monomethylated and dimethylated 'Lys-20' on histone H4. Binds histone H3 peptides that are monomethylated or dimethylated on 'Lys-4', 'Lys-9' or 'Lys-27'.	chr22:41605776 c.101G>C(E2)	p.34,R>P	0.09	0.615
TCP1	T-complex protein 1 subunit alpha, Molecular chaperone; assists the folding of proteins upon ATP hydrolysis. As part of the BBS/CCT complex may play a role in the assembly of BBSome, a complex involved in ciliogenesis regulating transports vesicles to the cilia. Known to play a role, in vitro, in the folding of actin and tubulin.	chr6:160202072 c.403C>T(E7)	p.135,G>S	0.06	0.581
SYNPO	Synaptopodin, Actin-associated protein that may play a role in modulating actin-based shape and motility of dendritic spines and renal podocyte foot processes. Seems to be essential for the formation of spine apparatuses in spines of telencephalic neurons, which is involved in synaptic plasticity (By similarity).	chr5:150029575 c.1738C>A(E2)	p.580,P>T	0	0.999
SLC25A4	Solute carrier family 25 member 47, Uncoupling protein which may catalyze the physiological 'proton leak' in liver. Overexpression induces the dissipation of mitochondrial membrane potential.	chr14:100792507 c.86C>T(E3)	p.29,T>M	0	0.699
NFAT5	Nuclear factor of activated T-cells 5, Transcription factor involved in the transcriptional regulation of osmoprotective and inflammatory genes. Regulates hypertonicity-induced cellular accumulation of osmolytes.	chr16:69680960 c.1A>G(E5)	p.1,M>V	0.13	0.448

Table S3 - List of 489 non-synonymous and frame-shifting variants which were predicted to be damaging (X: termination codon).

Gene	Uniprot function	Genome position (hg19)	AA position & change	SIFT	Polyphen-2
CTAG2	Cancer/testis antigen 2	chrX:153881773 c.17T>C(E1)	p.6,Q>R	0.28	0
ZNF600	Zinc finger protein 600, May be involved in transcriptional regulation.	chr19:53270017 c.992T>C(E3)	p.331,K>R	0.06	0.004
RGS10	Regulator of G-protein signaling 10, Regulates G protein-coupled receptor signaling cascades, including signaling downstream of the muscarinic acetylcholine receptor CHRM2. Inhibits signal transduction by increasing the GTPase activity of G protein alpha subunits, thereby driving them into their inactive GDP-bound form. Modulates the activity of potassium channels that are activated in response to CHRM2 signaling. Activity on GNAZ is inhibited by palmitoylation of the G-protein	chr10:121286832 c.154C>T(E2)	p.52,V>M	0.07	0.973
TPSG1	Tryptase gamma, serine-type endopeptidase activity	chr16:1273444 c.224G>T(E3)	p.75,T>K	0	0.959
DNAH7	Dynein heavy chain 7, axonemal, Force generating protein of respiratory cilia. Produces force towards the minus ends of microtubules. Dynein has ATPase activity; the force-producing power stroke is thought to occur on release of ADP (By similarity).	chr2:196726471 c.7706G>A(E42)	p.2569,T>I	0.01	0.982
		chr2:196729443 c.6936G>C(E41)	p.2312,N>K		
SLC37A3	Sugar phosphate exchanger 3, transmembrane transporter activity, anion and carbohydrate transport	chr7:140043294 c.1244A>G(E13)	p.415,L>P	0.32	0.008
CTSB	Thiol protease which is believed to participate in intracellular degradation and turnover of proteins. Has also been implicated in tumor invasion and metastasis.	chr8:11702705 c.949C>T(E10)	p.317,D>N	0.35	0.139
JOSD2	Josephin-2, Cleaves 'Lys-63'-linked poly-ubiquitin chains, and with lesser efficiency 'Lys-48'-linked poly-ubiquitin chains (in vitro). May act as a deubiquitinating enzyme.	chr19:51009440 c.557C>T(E5)	p.186,R>Q	0.25	0
MRPS9	28S ribosomal protein S9, mitochondrial, poly(A) binding, structural constituent of ribosome	chr2:105665741 c.248T>C(E2)	p.83,I>T	0	0.675

Table S3 - List of 489 non-synonymous and frame-shifting variants which were predicted to be damaging (X: termination codon).

Gene	Uniprot function	Genome position (hg19)	AA position & change	SIFT	Polyphen-2
EVPL	Envoplakin, Component of the cornified envelope of keratinocytes. May link the cornified envelope to desmosomes and intermediate filaments.	chr17:74003981 c.5305C>T(E22)	p.1769,G>S	0.01	1
EYA3	Eyes absent homolog 3, Tyrosine phosphatase that specifically dephosphorylates 'Tyr-142' of histone H2AX (H2AXY142ph). 'Tyr-142' phosphorylation of histone H2AX plays a central role in DNA repair and acts as a mark that distinguishes between apoptotic and repair responses to genotoxic stress. Promotes efficient DNA repair by dephosphorylating H2AX, promoting the recruitment of DNA repair complexes containing MDC1 (PubMed:19234442, PubMed:19351884). Its function as histone phosphatase probably explains its role in transcription regulation during organogenesis. Coactivates SIX1, and seems to coactivate SIX2, SIX4 and SIX5. The repression of precursor cell proliferation in myoblasts by SIX1 is switched to activation through recruitment of EYA3 to the SIX1-DACH1 complex and seems to be dependent on EYA3 phosphatase activity (By similarity). May be involved in development of the eye.	chr1:28362097 c.319C>T(E6)	p.107,V>I	0.19	0.014
NCR1	Natural cytotoxicity triggering receptor 1, Cytotoxicity-activating receptor that may contribute to the increased efficiency of activated natural killer (NK) cells to mediate tumor cell lysis.	chr19:55424204-55424206 c.877(E7)-c.879(E7): GAA deleted	E/_	_	_
EML6	Echinoderm microtubule-associated protein-like 6, May modify the assembly dynamics of microtubules, such that microtubules are slightly longer, but more dynamic.	chr2:55119614 c.2563G>A(E18)	p.855,G>R	0	0.988
OR10J3	Olfactory receptor 10J3, G-protein coupled receptor activity, Olfactory receptor activity	chr1:159283784 c.666G>C(E1)	p.222,I>M	0	0.988

Table S3 - List of 489 non-synonymous and frame-shifting variants which were predicted to be damaging (X: termination codon).

Gene	Uniprot function	Genome position (hg19)	AA position & change	SIFT	Polyphen-2
ARHGA	Rho GTPase-activating protein 24, Rho GTPase-activating protein involved in cell polarity, cell morphology and cytoskeletal organization. Acts as a GTPase activator for the Rac-type GTPase by converting it to an inactive GDP-bound state. Controls actin remodeling by inactivating Rac downstream of Rho leading to suppress leading edge protrusion and promotes cell retraction to achieve cellular polarity. Able to suppress RAC1 and CDC42 activity in vitro. Overexpression induces cell rounding with partial or complete disruption of actin stress fibers and formation of membrane ruffles, lamellipodia, and filopodia. Isoform 2 is a vascular cell-specific GAP involved in modulation of angiogenesis.	chr4:86916056 c.1249C>G(E9)	p.417,P>A	0.16	0.546
TMEM18	Transmembrane protein 189, ubiquitin protein ligase activity	chr20:48760074 c.206C>T(E2)	p.69,R>H	0.31	0.923
KIF16B	Kinesin-like protein KIF16B, Plus end-directed microtubule-dependent motor protein involved in endosome transport and receptor recycling and degradation. Regulates the plus end motility of early endosomes and the balance between recycling and degradation of receptors such as EGF receptor (EGFR) and FGF receptor (FGFR). Regulates the Golgi to endosome transport of FGFR-containing vesicles during early development, a key process for developing basement membrane and epiblast and primitive endoderm lineages during early postimplantation development.	chr20:16360515 c.2132C>T(E19)	p.711,R>Q	0.16	0.011
C10orf82	Uncharacterized protein C10orf82,	chr10:118424362 c.371G>A(E4)	p.124,T>M	0.06	0.529

Table S3 - List of 489 non-synonymous and frame-shifting variants which were predicted to be damaging (X: termination codon).

Gene	Uniprot function	Genome position (hg19)	AA position & change	SIFT	Polyphen-2
ELP5	Elongator complex protein 5, Acts as subunit of the RNA polymerase II elongator complex, which is a histone acetyltransferase component of the RNA polymerase II (Pol II) holoenzyme and is involved in transcriptional elongation. Elongator may play a role in chromatin remodeling and is involved in acetylation of histones H3 and probably H4. Involved in cell migration (By similarity). May be involved in TP53-mediated transcriptional regulation.	chr17:7156322 IVS4+5G>A splicing site	splicing region	_	_
DMRT3	Doublesex- and mab-3-related transcription factor 3, Probable transcription factor that plays a role in configuring the spinal circuits controlling stride in vertebrates. Involved in neuronal specification within specific subdivision of spinal cord neurons and in the development of a coordinated locomotor network controlling limb movements. May regulate transcription during sexual development (By similarity).	chr9:990401 c.815A>C(E2)	p.272,K>T	0	0.982
LEPREL2	Prolyl 3-hydroxylase 3, Has prolyl 3-hydroxylase activity catalyzing the post-translational formation of 3-hydroxyproline in -Xaa-Pro-Gly-sequences in collagens, especially types IV and V.	chr12:6938023-6938024 c.418(E1)-c.419(E1): G inserted	p.140,R>Rfs57	_	_
HSP90A	Heat shock protein HSP 90-alpha, Molecular chaperone that promotes the maturation, structural maintenance and proper regulation of specific target proteins involved for instance in cell cycle control and signal transduction. Undergoes a functional cycle that is linked to its ATPase activity. This cycle probably induces conformational changes in the client proteins, thereby causing their activation. Interacts dynamically with various co-chaperones that modulate its substrate recognition, ATPase cycle and chaperone function. Binds bacterial lipopolysaccharide (LPS) et mediates LPS-	chr14:102551262 c.1103T>C(E6)	p.368,E>G	0.07	0

Table S3 - List of 489 non-synonymous and frame-shifting variants which were predicted to be damaging (X: termination codon).

Gene	Uniprot function	Genome position (hg19)	AA position & change	SIFT	Polyphen-2
	induced inflammatory response, including TNF secretion by monocytes.				
LCE4A	Late cornified envelope protein 4A, Precursors of the cornified envelope of the stratum corneum.	chr1:152681680-152681681 c.129(E1)-c.130(E1): AGCTCTGGGGGCTGCTGT inserted	43-44 _/SSGGCX	_	_
SQRDL	Sulfide:quinone oxidoreductase, mitochondrial, Catalyzes the oxidation of hydrogen sulfide with the help of a quinone, such as ubiquinone, giving rise to thiosulfate and ultimately to sulfane (molecular sulfur) atoms. Requires an additional electron acceptor; can use sulfite, sulfide or cyanide (in vitro).	chr15:45968331 c.687C>G(E7)	p.229,F>L	0	0.868
RANBP3	Ran-binding protein 3, Acts as a cofactor for XPO1/CRM1-mediated nuclear export, perhaps as export complex scaffolding protein. Bound to XPO1/CRM1, stabilizes the XPO1/CRM1-cargo interaction. In the absence of Ran-bound GTP prevents binding of XPO1/CRM1 to the nuclear pore complex. Binds to CHC1/RCC1 and increases the guanine nucleotide exchange activity of CHC1/RCC1. Recruits XPO1/CRM1 to CHC1/RCC1 in a Ran-dependent manner. Negative regulator of TGF-beta signaling through interaction with the R-SMAD proteins, SMAD2 and SMAD3, and mediating their nuclear export.	chr19:5917924 c.1526C>T(E16)	p.509,R>H	0.36	0.042
URB1	Nucleolar pre-ribosomal-associated protein 1, poly(A) RNA binding	chr21:33721635 c.2990G>A(E21)	p.997,S>L	0.17	0.004
ARHGA	Rho GTPase-activating protein 4, Inhibitory effect on stress fiber organization. May down-regulate Rho-like GTPase in hematopoietic cells.	chrX:153175826 c.2075T>C(E18)	p.692,D>G	0.02	0.019
TAAR8	Trace amine-associated receptor 8, Orphan receptor. Could be a receptor for trace amines. Trace amines are biogenic amines present in very low levels in mammalian tissues. Although some trace amines have clearly defined roles as neurotransmitters in invertebrates, the extent to	chr6:132874814 c.983A>C(E1)	p.328,D>A	0.1	0.011

Table S3 - List of 489 non-synonymous and frame-shifting variants which were predicted to be damaging (X: termination codon).

Gene	Uniprot function	Genome position (hg19)	AA position & change	SIFT	Polyphen-2
	which they function as true neurotransmitters in vertebrates has remained speculative. Trace amines are likely to be involved in a variety of physiological functions that have yet to be fully understood.				
TTLL12	Tubulin--tyrosine ligase-like protein 12, ATP binding	chr22:43564923 IVS12-5G>T splicing cite	splicing region	_	_
LSR	Lipolysis-stimulated lipoprotein receptor, Probable role in the clearance of triglyceride-rich lipoprotein from blood. Binds chylomicrons, LDL and VLDL in presence of free fatty acids and allows their subsequent uptake in the cells (By similarity).	chr19:35758198 c.1415G>A(E8)	p.472,R>H	0.33	0.032
ZZEF1	Zinc finger ZZ-type and EF-hand domain-containing protein 1, calcium and zinc ion binding	chr17:3921203 c.7568G>C(E47)	p.2523,P>R	0	0.988
C10orf12	Acyl-coenzyme A synthetase ACSM6, mitochondrial, ATP and GTP binding and butyrate-CoA ligase activity, metal ion binding	chr10:96961794-96961794 c.245(E3): A deleted	p.82,E>Efs10	_	_
UQCRC1	Cytochrome b-c1 complex subunit 1, mitochondrial, This is a component of the ubiquinol-cytochrome c reductase complex (complex III or cytochrome b-c1 complex), which is part of the mitochondrial respiratory chain. This protein may mediate formation of the complex between cytochromes c and c1.	chr3:48638410 c.964C>T(E8)	p.322,V>M	0.14	0.01
RFX1	MHC class II regulatory factor RFX1, Regulatory factor essential for MHC class II genes expression. Binds to the X boxes of MHC class II genes. Also binds to an inverted repeat (ENH1) required for hepatitis B virus genes expression and to the most upstream element (alpha) of the RPL30 promoter.	chr19:14083728 c.1141C>T(E9)	p.381,G>R	0.04	0.214
GLS	Glutaminase kidney isoform, mitochondrial, Catalyzes the first reaction in the primary pathway for the renal catabolism of glutamine. Plays a role in maintaining acid-base homeostasis. Regulates	chr2:191746076 c.266A>C(E1)	p.89,H>P	0.1	0.002

Table S3 - List of 489 non-synonymous and frame-shifting variants which were predicted to be damaging (X: termination codon).

Gene	Uniprot function	Genome position (hg19)	AA position & change	SIFT	Polyphen-2
	the levels of the neurotransmitter glutamate in the brain. Isoform 2 lacks catalytic activity.				
LCE1E	Late cornified envelope protein 1E, Precursors of the cornified envelope of the stratum corneum.	chr1:152759836 c.61C>A(E2)	p.21,P>T	0.18	0
OR51S1	Olfactory receptor 51S1, Odorant receptor.	chr11:4870245 c.194C>T(E1)	p.65,R>H	0.03	0.967
UACA	Uveal autoantigen with coiled-coil domains and ankyrin repeats, Regulates APAF1 expression and plays an important role in the regulation of stress-induced apoptosis. Promotes apoptosis by regulating three pathways, apoptosome up-regulation, LGALS3/galectin-3 down-regulation and NF-kappa-B inactivation. Regulates the redistribution of APAF1 into the nucleus after proapoptotic stress. Down-regulates the expression of LGALS3 by inhibiting NFKB1 (By similarity). Modulates isoactin dynamics to regulate the morphological alterations required for cell growth and motility. Interaction with ARF6 may modulate cell shape and motility after injury. May be involved in multiple neurite formation (By similarity).	chr15:70952513 c.4117T>G(E18)	p.1373,T>P	0	0.996
C16orf58	RUS1 family protein C16orf58,	chr16:31504371 IVS9-2T>C splicing cite		_	_
FOXP4	Forkhead box protein P4, Transcriptional repressor that represses lung-specific expression.	chr6:41554795 c.559C>G(E6)	p.187,Q>E	0.07	0.156
C8orf47	Glutamate-rich protein 5,	chr8:99101813 c.568A>G(E2)	p.190,T>A	0.15	0
TBC1D10	TBC1 domain family member 10A, Acts as GTPase-activating protein for RAB27A, but not for RAB2A, RAB3A, nor RAB4A.	chr22:30688611 c.1301G>C(E9)	p.434,P>R	0.34	0.032
MRPS23	28S ribosomal protein S23, mitochondrial, poly(A) RNA binding, structural constituent of ribosome	chr17:55918596 c.241C>T(E3)	p.81,G>S	0.02	0.897
OBSCN	Obscurin, Involved in myofibrillogenesis. Seems to be involved in assembly of myosin into sarcomeric A bands in striated muscle. Isoform 3	chr1:228404888 c.2552C>T(E8)	p.851,T>M	_	0.998

Table S3 - List of 489 non-synonymous and frame-shifting variants which were predicted to be damaging (X: termination codon).

Gene	Uniprot function	Genome position (hg19)	AA position & change	SIFT	Polyphen-2
	together with ANK1 isoform Mu17/Ank1.5 may provide a molecular link between the sarcoplasmic reticulum and myofibrils.	chr1:228553131 IVS81-6C>T splicing cite	splicing region	_	_
HAVCR2	Hepatitis A virus cellular receptor 2, Cell surface receptor implicated in modulating innate and adaptive immune responses. Generally accepted to have an inhibiting function. Reports on stimulating functions suggest that the activity may be influenced by the cellular context and/or the respective ligand (PubMed:24825777). Regulates macrophage activation (PubMed:11823861). Inhibits T-helper type 1 lymphocyte (Th1)-mediated auto- and alloimmune responses and promotes immunological tolerance (PubMed:14556005). In CD8+ cells attenuates TCR-induced signaling, specifically by blocking NF-kappaB and NFAT promoter activities resulting in the loss of IL-2 secretion. The function may implicate its association with LCK proposed to impair phosphorylation of TCR subunits, and/or LGALS9-dependent recruitment of PTPRC to the immunological synapse (PubMed:24337741, PubMed:26492563). In contrast, shown to activate TCR-induced signaling in T-cells probably implicating ZAP70, LCP2, LCK and FYN (By similarity). Expressed on Treg cells can inhibit Th17 cell responses (PubMed:24838857). Receptor for LGALS9 (PubMed:16286920, PubMed:24337741). Binding to LGALS9 is believed to result in suppression of T-cell responses; the resulting apoptosis of antigen-specific cells may implicate HAVCR2 phosphorylation and disruption of its association with BAG6. Binding to LGALS9 is proposed to be involved in innate immune response to intracellular pathogens. Expressed on Th1 cells	chr5:156533787 c.245T>C(E2)	p.82,Y>C	0	1

Table S3 - List of 489 non-synonymous and frame-shifting variants which were predicted to be damaging (X: termination codon).

Gene	Uniprot function	Genome position (hg19)	AA position & change	SIFT	Polyphen-2
	interacts with LGALS9 expressed on Mycobacterium tuberculosis-infected macrophages to stimulate antibactericidal activity including IL-1 beta secretion and to restrict intracellular bacterial growth (By similarity). However, the function as receptor for LGALS9 has been challenged (PubMed:23555261). Also reported to enhance CD8+ T-cell responses to an acute infection such as by Listeria monocytogenes (By similarity). Receptor for phosphatidylserine (PtSer); PtSer-binding is calcium-dependent. May recognize PtSer on apoptotic cells leading to their phagocytosis. Mediates the engulfment of apoptotic cells by dendritic cells. Expressed on T-cells, promotes conjugation but not engulfment of apoptotic cells. Expressed on dendritic cells (DCs) positively regulates innate immune response and in synergy with Toll-like receptors promotes secretion of TNF-alpha. In tumor-imfiltrating DCs suppresses nucleic acid-mediated innate immune repsonse by interaction with HMGB1 and interfering with nucleic acid-sensing and trafficking of nucleid acids to endosomes (By similarity). Expressed on natural killer (NK) cells acts as a coreceptor to enhance IFN-gamma production in response to LGALS9 (PubMed:22323453). In contrast, shown to suppress NK cell-mediated cytotoxicity (PubMed:22383801). Negatively regulates NK cell function in LPS-induced endotoxic shock (By similarity).				
RASAL3	RAS protein activator like-3, Functions as a Ras GTPase-activating protein. Plays an important role in the expansion and functions of natural killer T (NKT) cells in the liver by negatively regulating RAS activity and the down-stream ERK signaling	chr19:15565621 c.1805C>T(E12)	p.602,R>Q	0.02	0.735

Table S3 - List of 489 non-synonymous and frame-shifting variants which were predicted to be damaging (X: termination codon).

Gene	Uniprot function	Genome position (hg19)	AA position & change	SIFT	Polyphen-2
	pathway.				
ENTPD7	Ectonucleoside triphosphate diphosphohydrolase 7, Preferentially hydrolyzes nucleoside 5'-triphosphates. The order of activity with respect to possible substrates is UTP > GTP > CTP.	chr10:101464280 c.1655A>C(E13)	p.552,Y>S	0	0.924
PPL	Periplakin, Component of the cornified envelope of keratinocytes. May link the cornified envelope to desmosomes and intermediate filaments. May act as a localization signal in PKB/AKT-mediated signaling.	chr16:4940825 c.2066C>T(E17)	p.689,R>H	0.07	0.008
		chr16:4949345 c.647T>A(E7)	p.216,Q>L	0.11	0.19
ITGAX	Integrin alpha-X, Integrin alpha-X/beta-2 is a receptor for fibrinogen. It recognizes the sequence G-P-R in fibrinogen. It mediates cell-cell interaction during inflammatory responses. It is especially important in monocyte adhesion and chemotaxis.	chr16:31374256 c.1360A>C(E13)	p.454,I>L	0.01	0.03
TRIM66	Tripartite motif-containing protein 66, May function as transcription repressor; The repressive effects are mediated, at least in part, by recruitment of deacetylase activity. May play a role as negative regulator of postmeiotic genes acting through CBX3 complex formation and centromere association (By similarity).	chr11:8646450 c.2201T>G(E11)	p.734,Q>P	0.3	0.759
PAQR3	Progestin and adipoQ receptor family member 3, Functions as a spatial regulator of RAF1 kinase by sequestrating it to the Golgi.	chr4:79847790 c.587G>A(E4)	p.196,T>M	0.03	0.764
ZFAND1	AN1-type zinc finger protein 1	chr8:82627111 c.286G>A(E5)	p.96,H>Y	0	0.999
AMZ2	Archaemetzincin-2, Zinc metalloprotease. Exhibits activity against angiotensin-3 in vitro. Does not hydrolyze either neurogranin or angiotensin-2.	chr17:66250649 c.691A>G(E6)	p.231,I>V	1	0.006
PEAK1	Pseudopodium-enriched atypical kinase 1, Tyrosine kinase that may play a role in cell spreading and migration on fibronectin. May directly or indirectly affect phosphorylation levels	chr15:77407280 c.4459C>T(E8)	p.1487,D>N	0.02	0.981

Table S3 - List of 489 non-synonymous and frame-shifting variants which were predicted to be damaging (X: termination codon).

Gene	Uniprot function	Genome position (hg19)	AA position & change	SIFT	Polyphen-2
	of cytoskeleton-associated proteins MAPK1/ERK and PXN.				
SLC38A6	Probable sodium-coupled neutral amino acid transporter 6, Probable sodium-dependent amino acid/proton antiporter, could be a neuronal transporter for glutamate.	chr14:61512802 c.842T>C(E12)	p.281,M>T	0.01	0.882
		chr14:61517256 c.952G>A(E13)	p.318,G>S	0.32	0.079
EFCAB6	EF-hand calcium-binding domain-containing protein 6, Negatively regulates the androgen receptor by recruiting histone deacetylase complex, and protein DJ-1 antagonizes this inhibition by abrogation of this complex.	chr22:44107407 c.979C>T(E10)	p.327,D>N	0.33	1
FGFR4	Fibroblast growth factor receptor 4, Tyrosine-protein kinase that acts as cell-surface receptor for fibroblast growth factors and plays a role in the regulation of cell proliferation, differentiation and migration, and in regulation of lipid metabolism, bile acid biosynthesis, glucose uptake, vitamin D metabolism and phosphate homeostasis. Required for normal down-regulation of the expression of CYP7A1, the rate-limiting enzyme in bile acid synthesis, in response to FGF19. Phosphorylates PLCG1 and FRS2. Ligand binding leads to the activation of several signaling cascades. Activation of PLCG1 leads to the production of the cellular signaling molecules diacylglycerol and inositol 1,4,5-trisphosphate. Phosphorylation of FRS2 triggers recruitment of GRB2, GAB1, PIK3R1 and SOS1, and mediates activation of RAS, MAPK1/ERK2, MAPK3/ERK1 and the MAP kinase signaling pathway, as well as of the AKT1 signaling pathway. Promotes SRC-dependent phosphorylation of the matrix protease MMP14 and its lysosomal degradation. FGFR4 signaling is down-regulated by receptor internalization and degradation; MMP14 promotes internalization and degradation of FGFR4.	chr5:176518037 c.535A>G(E5)	p.179,T>A	0.27	0.045

Table S3 - List of 489 non-synonymous and frame-shifting variants which were predicted to be damaging (X: termination codon).

Gene	Uniprot function	Genome position (hg19)	AA position & change	SIFT	Polyphen-2
	Mutations that lead to constitutive kinase activation or impair normal FGFR4 inactivation lead to aberrant signaling.				
CLIP2	CAP-Gly domain-containing linker protein 2, Seems to link microtubules to dendritic lamellar body (DLB), a membranous organelle predominantly present in bulbous dendritic appendages of neurons linked by dendrodendritic gap junctions. May operate in the control of brain-specific organelle translocations (By similarity).	chr7:73790805 c.2074G>A(E10)	p.692,A>T	0.05	0.031
LRP10	Low-density lipoprotein receptor-related protein 10,Probable receptor, which is involved in the internalization of lipophilic molecules and/or signal transduction. May be involved in the uptake of lipoprotein APOE in liver (By similarity).	chr14:23346025 c.1552G>A(E6)	p.518,D>N	0.02	0.448
SERHL2	Serine hydrolase-like protein 2, Probable serine hydrolase. May be related to cell muscle hypertrophy.	chr22:42950033 c.7G>A(E1)	p.3,E>K	0.13	0.067
RPAIN	RPA-interacting protein, Mediates the import of RPA complex into the nucleus, possibly via some interaction with importin beta. Isoform 2 is sumoylated and mediates the localization of RPA complex into the PML body of the nucleus, thereby participating in RPA function in DNA metabolism.	chr17:5329392 c.415G>T(E4)	p.139,V>L	0.04	0.998
ZNF195	Zinc finger protein 195, May be involved in transcriptional regulation.	chr11:3380998 c.1171C>T(E5)	p.391,E>K	0.04	0.924
PRAMEF	Putative PRAME family member 13, negative regulation of apoptotic process, cell differentiation, tetinoic acid receptor signalling pathway, transcription and cell proliferation	chr1:13448199 c.1276C>T(E4)	p.426,D>N	0.38	0.001

Table S3 - List of 489 non-synonymous and frame-shifting variants which were predicted to be damaging (X: termination codon).

Gene	Uniprot function	Genome position (hg19)	AA position & change	SIFT	Polyphen-2
AHR	Aryl hydrocarbon receptor, Ligand-activated transcriptional activator. Binds to the XRE promoter region of genes it activates. Activates the expression of multiple phase I and II xenobiotic chemical metabolizing enzyme genes (such as the CYP1A1 gene). Mediates biochemical and toxic effects of halogenated aromatic hydrocarbons. Involved in cell-cycle regulation. Likely to play an important role in the development and maturation of many tissues. Regulates the circadian clock by inhibiting the basal and circadian expression of the core circadian component PER1. Inhibits PER1 by repressing the CLOCK-ARNTL/BMAL1 heterodimer mediated transcriptional activation of PER1.	chr7:17378796 c.1347C>G(E10)	p.449,D>E	0.38	0.002
TCN1	Transcobalamin-1, Binds vitamin B12 with femtomolar affinity and protects it from the acidic environment of the stomach.	chr11:59631425 c.214T>A(E2)	p.72,M>L	0.1	0
BASP1	Brain acid soluble protein 1, protein domain specific binding, trascription corepressor activity	chr5:17275552 c.227C>T(E2)	p.76,A>V	0.02	0
CCDC74	Coiled-coil domain-containing protein 74B,	chr2:130897638 c.710C>T(E6)	p.237,S>N	0.46	0.682
PLXNB3	Plexin-B3, Receptor for SEMA5A that plays a role in axon guidance, invasive growth and cell migration. Stimulates neurite outgrowth and mediates Ca2+/Mg2+-dependent cell aggregation. In glioma cells, SEMA5A stimulation of PLXNB3 results in the disassembly of F-actin stress fibers, disruption of focal adhesions and cellular collapse as well as inhibition of cell migration and invasion through ARHGDIA-mediated inactivation of RAC1.	chrX:153036810 c.2368G>A(E14)	p.790,V>I	0.2	0.477
PLXNB1	Plexin-B1, Receptor for SEMA4D. Plays a role in RHOA activation and subsequent changes of the actin cytoskeleton. Plays a role in axon guidance, invasive growth and cell migration.	chr3:48459680 c.3142G>A(E15)	p.1048,R>C	0.01	0.208
AXDND1	Axonemal dynein light chain domain-containing	chr1:179452274 c.2009C>T(E18)	p.670,A>V	0.28	0.022

Table S3 - List of 489 non-synonymous and frame-shifting variants which were predicted to be damaging (X: termination codon).

Gene	Uniprot function	Genome position (hg19)	AA position & change	SIFT	Polyphen-2
	protein 1,				
SMCR8	Smith-Magenis syndrome chromosomal region candidate gene 8 protein,	chr17:18221250 c.2147A>G(E1)	p.716,Q>R	0.01	0.993
NUP133	Nuclear pore complex protein Nup133, nucleocytoplasmic transporter activity, structural constituent of nuclear pore	chr1:229636603 c.413A>G(E4)	p.138,V>A	0.02	0.063
POC5	Centrosomal protein POC5, Essential for the assembly of the distal half of centrioles, required for centriole elongation.	chr5:74998606 c.337G>A(E5)	p.113,P>S	0.25	0.023
TMPRSS	Transmembrane protease serine 13, scavenger receptor activity, serine-type endopeptidase activity	chr11:117789313-117789327, c.262(E2)-c.248	p.83-88 QASPAR/R	_	_
MIOX	Inositol oxygenase,	chr22:50926366 c.229G>A(E4)	p.77,V>M	0.15	0.262
PAGE5	P antigen family member 5, G antigen family E member 1 Prostate-associated gene 5 protein	chrX:55249049 IVS3-5T>C splicing site	splicing region	_	_
MLL5	Histone-lysine N-methyltransferase 2E, Histone methyltransferase that specifically mono- and dimethylates 'Lys-4' of histone H3 (H3K4me1 and H3K4me2). H3 'Lys-4' methylation represents a specific tag for epigenetic transcriptional activation. Key regulator of hematopoiesis involved in terminal myeloid differentiation and in the regulation of hematopoietic stem cell (HSCs) self-renewal by a mechanism that involves DNA methylation. Plays an essential role in retinoic-acid-induced granulopoiesis by acting as a coactivator of RAR-alpha (RARA) in target gene promoters. Also acts as an important cell cycle regulator, participating in cell cycle regulatory network machinery at multiple cell cycle stages. Required to suppress inappropriate expression of S-phase-promoting genes and maintain expression of determination genes in quiescent cells. Overexpression inhibits cell cycle progression, while knockdown induces cell cycle arrest at both	chr7:104750932 c.3853T>G(E24)	p.1285,S>A	0.07	0.097

Table S3 - List of 489 non-synonymous and frame-shifting variants which were predicted to be damaging (X: termination codon).

Gene	Uniprot function	Genome position (hg19)	AA position & change	SIFT	Polyphen-2
	the G1 and G2/M phases. Isoform NKp44L: Cellular ligand for NCR2/NKp44, may play a role as a danger signal in cytotoxicity and NK-cell-mediated innate immunity.				
SLC22A1	Solute carrier family 22 member 10， inorganic anion exchanger activity， sodium-independent organic anion transmembrane transporter activity	chr11:63067061 c.1030C>T(E6)	p.344,P>S	0.02	0.913
GPRIN2	G protein-regulated inducer of neurite outgrowth 2, May be involved in neurite outgrowth.	chr10:46999591-c.711(E3)46999592-c.712(E3	P. 237-238 -/MKE	_	_
FAM189	Protein FAM189B, WW domain binding	chr1:155217643 c.1883C>T(E11)	p.628,R>H	0.03	0.968
PDGFA	Platelet-derived growth factor subunit A,Growth factor that plays an essential role in the regulation of embryonic development, cell proliferation, cell migration, survival and chemotaxis. Potent mitogen for cells of mesenchymal origin. Required for normal lung alveolar septum formation during embryogenesis, normal development of the gastrointestinal tract, normal development of Leydig cells and spermatogenesis. Required for normal oligodendrocyte development and normal myelination in the spinal cord and cerebellum. Plays an important role in wound healing. Signaling is modulated by the formation of heterodimers with PDGFB (By similarity).	chr7:552044 c.209G>A(E3)	p.70,A>V	0.13	0.014
CHMP4C	Charged multivesicular body protein 4c, Probable core component of the endosomal sorting required for transport complex III (ESCRT-III) which is involved in multivesicular bodies (MVBs) formation and sorting of endosomal cargo proteins into MVBs. MVBs contain intraluminal vesicles (ILVs) that are generated by invagination and scission from the limiting membrane of the endosome and mostly are delivered to lysosomes enabling degradation of membrane proteins, such	chr8:82670524 c.631C>T(E4)	p.211,R>X(23)	_	_

Table S3 - List of 489 non-synonymous and frame-shifting variants which were predicted to be damaging (X: termination codon).

Gene	Uniprot function	Genome position (hg19)	AA position & change	SIFT	Polyphen-2
	as stimulated growth factor receptors, lysosomal enzymes and lipids. The MVB pathway appears to require the sequential function of ESCRT-O, -I,-II and -III complexes. ESCRT-III proteins mostly dissociate from the invaginating membrane before the ILV is released. The ESCRT machinery also functions in topologically equivalent membrane fission events, such as the terminal stages of cytokinesis and the budding of enveloped viruses (HIV-1 and other lentiviruses). Key component of the cytokinesis checkpoint, a process required to delay abscission to prevent both premature resolution of intercellular chromosome bridges and accumulation of DNA damage: upon phosphorylation by AURKB, together with ZFYVE19/ANCHR, retains abscission-competent VPS4 (VPS4A and/or VPS4B) at the midbody ring until abscission checkpoint signaling is terminated at late cytokinesis. Deactivation of AURKB results in dephosphorylation of CHMP4C followed by its dissociation from ANCHR and VPS4 and subsequent abscission. ESCRT-III proteins are believed to mediate the necessary vesicle extrusion and/or membrane fission activities, possibly in conjunction with the AAA ATPase VPS4. Involved in HIV-1 p6- and p9-dependent virus release. CHMP4A/B/C are required for the exosomal release of SDCBP, CD63 and syndecan				
SLC22A1	Solute carrier family 22 member 16, High affinity carnitine transporter; the uptake is partially sodium-ion dependent. Thought to mediate the L-carnitine secretion mechanism from testis epididymal epithelium into the lumen which is involved in the maturation of spermatozoa. Also transports organic cations such as tetraethylammonium (TEA) and doxorubicin. The	chr6:110778051 c.223C>T(E2)	p.75,G>R	0.07	0.187

Table S3 - List of 489 non-synonymous and frame-shifting variants which were predicted to be damaging (X: termination codon).

Gene	Uniprot function	Genome position (hg19)	AA position & change	SIFT	Polyphen-2
	uptake of TEA is inhibited by various organic cations. The uptake of doxorubicin is sodium-independent.				
GPRIN1	G protein-regulated inducer of neurite outgrowth 1,May be involved in neurite outgrowth.	chr5:176025287 c.1549C>T(E2)	p.517,G>R	0.2	0.976
SLC15A3	transporter activity	chr11:60708671 c.1199C>T(E5)	p.400,R>Q	0.05	0.949
MEX3D	RNA-binding protein MEX3D, RNA binding protein, may be involved in post-transcriptional regulatory mechanisms.	chr19:1556482 c.1036T>G(E2)	p.346,T>P	0.26	0.002
MBOAT	Lysophospholipid acyltransferase 2, Acyltransferase which mediates the conversion of lysophosphatidylethanolamine (1-acyl-sn-glycero-3-phosphoethanolamine or LPE) into phosphatidylethanolamine (1,2-diacyl-sn-glycero-3-phosphoethanolamine or PE) (LPEAT activity). Catalyzes also the acylation of lysophosphatidic acid (LPA) into phosphatidic acid (PA) (LPAAT activity). Has also a very weak lysophosphatidylcholine acyltransferase (LPCAT activity). Prefers oleoyl-CoA as the acyl donor. Lysophospholipid acyltransferases (LPLATs) catalyze the reacylation step of the phospholipid remodeling pathway also known as the Lands cycle.	chr2:8998871 c.1501T>C(E13)	p.501,T>A	0.25	0.021
OR13D1	Olfactory receptor 13D1	chr9:107457083 c.381T>G(E1)	p.127,I>M	0.07	0.064
RAB32	Ras-related protein Rab-32, Acts as an A-kinase anchoring protein by binding to the type II regulatory subunit of protein kinase A and anchoring it to the mitochondrion. Also involved in synchronization of mitochondrial fission. Plays a role in the maturation of phagosomes that engulf pathogens, such as S.aureus and M.tuberculosis.	chr6:146875616 c.553G>T(E3)	p.185,A>S	0.12	0.057
AFF3	AF4/FMR2 family member 3, Putative transcription activator that may function in lymphoid development and oncogenesis. Binds, in	chr2:100210336-100210342 c.1862(E14)-c.18	619-620 TS/X 620-621 SA/_	_	_

Table S3 - List of 489 non-synonymous and frame-shifting variants which were predicted to be damaging (X: termination codon).

Gene	Uniprot function	Genome position (hg19)	AA position & change	SIFT	Polyphen-2
	vitro, to double-stranded DNA.				
CD1B	T-cell surface glycoprotein CD1b, Antigen-presenting protein that binds self and non-self lipid and glycolipid antigens and presents them to T-cell receptors on natural killer T-cells.	chr1:158299426 c.620G>A(E4)	p.207,A>V	0	0.998
CSNK1A	Casein kinase I isoform alpha-like, Casein kinases are operationally defined by their preferential utilization of acidic proteins such as caseins as substrates. It can phosphorylate a large number of proteins. Participates in Wnt signaling (By similarity).	chr13:37679333 c.61G>A(E1)	p.21,R>W	0	0.171
NASP	Nuclear autoantigenic sperm protein, Required for DNA replication, normal cell cycle progression and cell proliferation. Forms a cytoplasmic complex with HSP90 and H1 linker histones and stimulates HSP90 ATPase activity. NASP and H1 histone are subsequently released from the complex and translocate to the nucleus where the histone is released for binding to DNA (By similarity).	chr1:46073517 c.742G>A(E4)	p.248,D>N	0.13	0.009
NOS1	Nitric oxide synthase, brain, Produces nitric oxide (NO) which is a messenger molecule with diverse functions throughout the body. In the brain and peripheral nervous system, NO displays many properties of a neurotransmitter. Probably has nitrosylase activity and mediates cysteine S-nitrosylation of cytoplasmic target proteins such SRR.	chr12:117691485 c.2606A>G(E17)	p.869,L>P	0.32	0.003
FYB	FYN-binding protein, Acts as an adapter protein of the FYN and LCP2 signaling cascades in T-cells. Modulates the expression of interleukin-2 (IL-2). Involved in platelet activation. Prevents the degradation of SKAP1 and SKAP2. May play a role in linking T-cell signaling to remodeling of the actin cytoskeleton.	chr5:39126205 c.1970G>A(E12)	p.657,T>M	0.12	0.052

Table S3 - List of 489 non-synonymous and frame-shifting variants which were predicted to be damaging (X: termination codon).

Gene	Uniprot function	Genome position (hg19)	AA position & change	SIFT	Polyphen-2
DDIT4L	DNA damage-inducible transcript 4-like protein, Inhibits cell growth by regulating the TOR signaling pathway upstream of the TSC1-TSC2 complex and downstream of AKT1	chr4:101108989 c.427C>A(E3)	p.143,E>X(51)		
ANKRD3	Ankyrin repeat domain-containing protein 36A,	chr2:97852956 c.2063C>T(E31)	p.688,A>V	0.05	0.989
		chr2:97860471 c.2458C>T(E39)	p.820,R>W	0	0.919
NFS1	Cysteine desulfurase, mitochondrial,Catalyzes the removal of elemental sulfur from cysteine to produce alanine. It supplies the inorganic sulfur for iron-sulfur (Fe-S) clusters. May be involved in the biosynthesis of molybdenum cofactor.	chr20:34257566 c.1199C>T(E12)	p.400,S>N	0.07	0.018
PPFIA1	Liprin-alpha-1, May regulate the disassembly of focal adhesions. May localize receptor-like tyrosine phosphatases type 2A at specific sites on the plasma membrane, possibly regulating their interaction with the extracellular environment and their association with substrates.	chr11:70200527 c.2284G>A(E17)	p.762,V>I	0.42	0.035
MFSD6L	Major facilitator superfamily domain-containing protein 6-like,	chr17:8702369 c.70C>T(E1)	p.24,G>R	0	0.989
REV3L	DNA polymerase zeta catalytic subunit, Interacts with MAD2L2 to form the error prone DNA polymerase zeta involved in translesion DNA synthesis.	chr6:111628730 c.9086C>T(E31)	p.3029,R>Q	0.09	0.98
RIN3	E3 ubiquitin protein ligase RIN3, E3 ubiquitin protein ligase that acts as positive regulator of RPM1- and RPS2-dependent hypersensitive response (HR), in association with RIN2. Probably not required for RPM1 degradation during HR.	chr14:93154538-93154540 c.2899(E10)-c.290	p.967 G/—	—	—
DENND3	DENN domain-containing protein 3，Guanine nucleotide exchange factor (GEF) activating RAB12. Promotes the exchange of GDP to GTP, converting inactive GDP-bound RAB12 into its active GTP-bound form. Thereby, may play a role in protein transport from recycling endosomes to lysosomes regulating, for instance, the degradation	chr8:142199229 c.2989G>A(E19)	p.997,G>R	0.4	0.008

Table S3 - List of 489 non-synonymous and frame-shifting variants which were predicted to be damaging (X: termination codon).

Gene	Uniprot function	Genome position (hg19)	AA position & change	SIFT	Polyphen-2
	of the transferrin receptor.				
MAP3K1	Mitogen-activated protein kinase kinase kinase 19,	chr2:135744750 c.1692C>T(E7)	p.564,M>I	0.49	0.002
MB21D2	Protein MB21D2, protein complex binding	chr3:192517421 c.230T>C(E2)	p.77,D>G	0	0.996
LAX1	Lymphocyte transmembrane adapter 1, Negatively regulates TCR (T-cell antigen receptor)-mediated signaling in T-cells and BCR (B-cell antigen receptor)-mediated signaling in B-cells.	chr1:203743520 c.860G>C(E5)	p.287,S>T	0.01	0.173
DACT2	Dapper homolog 2, Involved in regulation of intracellular signaling pathways during development. Negatively regulates the Nodal signaling pathway, possibly by promoting the lysosomal degradation of Nodal receptors, such as TGFBR1. May be involved in control of the morphogenetic behavior of kidney ureteric bud cells by keeping cells epithelial and restraining their mesenchymal character. May play an inhibitory role in the re-epithelialization of skin wounds by attenuating TGF-beta signaling (By similarity).	chr6:168710917 c.589T>C(E3)	p.197,R>G	0.04	0.009
CHRDL2	Chordin-like protein 2, May inhibit BMPs activity by blocking their interaction with their receptors. Has a negative regulator effect on the cartilage formation/regeneration from immature mesenchymal cells, by preventing or reducing the rate of matrix accumulation (By similarity). Implicated in tumor angiogenesis. May play a role during myoblast and osteoblast differentiation, and maturation.	chr11:74408274 c.1244G>A(E11)	p.415,P>L	0	0.998
DNAJC2	DnaJ homolog subfamily C member 28, May have a role in protein folding or as a chaperone.	chr21:34860750-34860754 c.951(E2)-c.947(E2	p.316,L>Cfs12		
FOXD4L	Forkhead box protein D4-like 3, RNA polymerase II transcription factor activity, sequence-specific DNA binding, sequence-specific DNA binding	chr9:70918542 c.675C>G(E1)	p.225,H>Q	0.05	0.052
ZDHHC1	Probable palmitoyltransferase ZDHHC14, zinc ion binding	chr6:158074592 c.1001C>T(E8)	p.334,T>M	0.14	0.018

Table S3 - List of 489 non-synonymous and frame-shifting variants which were predicted to be damaging (X: termination codon).

Gene	Uniprot function	Genome position (hg19)	AA position & change	SIFT	Polyphen-2
HAO1	Hydroxyacid oxidase 1,Has 2-hydroxyacid oxidase activity. Most active on the 2-carbon substrate glycolate, but is also active on 2-hydroxy fatty acids, with high activity towards 2-hydroxy palmitate and 2-hydroxy octanoate.	chr20:7915210 c.210C>A(E2)	p.70,R>S	0.08	0.008
RBBP6	E3 ubiquitin-protein ligase RBBP6, E3 ubiquitin-protein ligase which promotes ubiquitination of YBX1, leading to its degradation by the proteasome (PubMed:18851979). May play a role as a scaffold protein to promote the assembly of the p53/TP53-MDM2 complex, resulting in increase of MDM2-mediated ubiquitination and degradation of p53/TP53; may function as negative regulator of p53/TP53, leading to both apoptosis and cell growth (By similarity). Regulates DNA-replication and the stability of chromosomal common fragile sites (CFSs) in a ZBTB38- and MCM10-dependent manner. Controls ZBTB38 protein stability and abundance via ubiquitination and proteasomal degradation, and ZBTB38 in turn negatively regulates the expression of MCM10 which plays an important role in DNA-replication	chr16:24582703 c.4316A>G(E18)	p.1439,N>S	0.42	0
LENG8	Leukocyte receptor cluster member 8,	chr19:54965736 c.554C>T(E6)	p.185,P>L	0.22	0.156
PSG1	Pregnancy-specific beta-1-glycoprotein 1,	chr19:43382236 c.259C>G(E2)	p.87,E>Q	1	0.001
ASTL	Astacin-like metalloendopeptidase, Oocyte-specific oolemmal receptor involved in sperm and egg adhesion and fertilization. Plays a role in the polyspermy inhibition. Probably acts as a protease for the post-fertilization cleavage of ZP2. Cleaves the sperm-binding ZP2 at the surface of the zona pellucida after fertilization and cortical granule exocytosis, rendering the zona pellucida unable to support further sperm binding (By similarity).	chr2:96798305 c.611C>T(E6	) p.204,R>H	0.05	0.052
SEMA3B	Semaphorin-3B,Inhibits axonal extension by providing local signals to specify territories	chr3:50306752-5306753 c.80(E2)-c.81(E2):C i	p.27,S>Sfs36	_	_

Table S3 - List of 489 non-synonymous and frame-shifting variants which were predicted to be damaging (X: termination codon).

Gene	Uniprot function	Genome position (hg19)	AA position & change	SIFT	Polyphen-2
	inaccessible for growing axons.				
ETAA1	Ewing's tumor-associated antigen 1,	chr2:67631958 c.2144C>T(E5)	p.715,P>L	0.13	0.549
OR13A1	Olfactory receptor 13A1	chr10:45799509 c.362A>G(E4)	p.121,F>S	0.03	0.318
ACTR5	Actin-related protein 5, Proposed core component of the chromatin remodeling INO80 complex which is involved in transcriptional regulation, DNA replication and probably DNA repair. Involved in DNA double-strand break repair and UV-damage excision repair.	chr20:37400374 c.1739C>T(E9)	p.580,P>L	0.01	0.242
BAG1	BAG family molecular chaperone regulator 1, Inhibits the chaperone activity of HSP70/HSC70 by promoting substrate release. Inhibits the pro-apoptotic function of PPP1R15A, and has anti-apoptotic activity. Markedly increases the anti-cell death function of BCL2 induced by various stimuli.	chr9:33264393-33264410 c.280(E1)-c.263(E1)	p.88-94 RSEELTL/L	_	_
ATAD3	ATPase family AAA domain-containing protein 3A, Essential for mitochondrial network organization, mitochondrial metabolism and cell growth at organism and cellular level. May play an important in mitochondrial protein synthesis. May also participate in mitochondrial DNA replication. May bind to mitochondrial DNA D-loops and contribute to nucleoid stability. Required for enhanced channeling of cholesterol for hormone-dependent steroidogenesis.	chr1:1452638 c.374C>A(E3)	p.125,S>X(510)		
ZBTB38	Zinc finger and BTB domain-containing protein 38, Transcriptional regulator with bimodal DNA-binding specificity. Binds with a higher affinity to methylated CpG dinucleotides in the consensus sequence 5'-CGCG-3' but can also bind to E-box elements (5'-CACGTG-3'). Can also bind specifically to a single methyl-CpG pair. Represses transcription in a methyl-CpG-dependent manner). Plays an important role in regulating DNA replication and common fragile sites (CFS)	chr3:141163655 c.2425G>A(E8)	p.809,A>T	0.39	0.001

Table S3 - List of 489 non-synonymous and frame-shifting variants which were predicted to be damaging (X: termination codon).

Gene	Uniprot function	Genome position (hg19)	AA position & change	SIFT	Polyphen-2
	stability in a RBBP6- and MCM10-dependent manner; represses expression of MCM10 which plays an important role in DNA-replication. Acts as a transcriptional activator. May be involved in the differentiation and/or survival of late postmitotic neurons (By similarity).				
CANX	Calnexin, Calcium-binding protein that interacts with newly synthesized glycoproteins in the endoplasmic reticulum. It may act in assisting protein assembly and/or in the retention within the ER of unassembled protein subunits. It seems to play a major role in the quality control apparatus of the ER by the retention of incorrectly folded proteins. Associated with partial T-cell antigen receptor complexes that escape the ER of immature thymocytes, it may function as a signaling complex regulating thymocyte maturation. Additionally it may play a role in receptor-mediated endocytosis at the synapse.	chr5:179135353 c.418C>A(E5) p.140,L>M r	p.140,L>M	0.17	0.222
UVRAG	UV radiation resistance-associated gene protein, Versatile protein that is involved in regulation of differenent cellular pathways implicated in membrane trafficking. Involved in regulation of the COPI-dependent retrograde transport from Golgi and the endoplasmic reticulum by associating with the NRZ complex; the function is dependent on its binding to phosphatidylinositol 3-phosphate (PtdIns3P) . During autophagy acts as regulatory subunit of the alternative PI3K complex II (PI3KC3-C2) that mediates formation of phosphatidylinositol 3-phosphate and is believed to be involved in maturation of autophagosomes and endocytosis. Activates lipid kinase activity of PIK3C3. Involved in the regulation of degradative endocytic trafficking and cytokinesis, and in regulation of ATG9A transport from the Golgi to	chr11:75728009 c.1211C>T(E12)	p.404,T>M	0.03	0.92

Table S3 - List of 489 non-synonymous and frame-shifting variants which were predicted to be damaging (X: termination codon).

Gene	Uniprot function	Genome position (hg19)	AA position & change	SIFT	Polyphen-2
	the autophagosome; the functions seems to implicate its association with PI3KC3-C2. Involved in maturation of autophagosomes and degradative endocytic trafficking independently of BECN1 but depending on its association with a class C Vps complex (possibly the HOPS complex); the association is also proposed to promote autophagosome recruitment and activation of Rab7 and endosome-endosome fusion events. Enhances class C Vps complex (possibly HOPS complex) association with a SNARE complex and promotes fusogenic SNARE complex formation during late endocytic membrane fusion. In case of negative-strand RNA virus infection is required for efficient virus entry, promotes endocytic transport of virions and is implicated in a VAMP8-specific fusogenic SNARE complex assembly .				
ZMAT5	Zinc finger matrin-type protein 5	chr22:30144409 c.125C>T(E2)	p.42,R>Q	0.01	0.967
ZMAT1	Zinc finger matrin-type protein 1,	chrX:101152902 c.444T>A(E5)	p.148,K>N	0.3	0.009
BCO2	Beta,beta-carotene 9',10'-oxygenase, Asymmetrically cleaves beta-carotene at the 9',10' double bond resulting in the formation of beta-apo-10'-carotenal and beta-ionone. Besides beta-carotene, lycopene is also oxidatively cleaved. The apocarotenals formed by this enzyme may be the precursors for the biosynthesis of retinoic acid or exert unknown physiological effects	chr11:112064369 c.364A>C(E3)	p.122,N>H	0	0.89
GRK4	G protein-coupled receptor kinase 4, Specifically phosphorylates the activated forms of G protein-coupled receptors. GRK4-alpha can phosphorylate rhodopsin and its activity is inhibited by calmodulin; the other three isoforms do not phosphorylate rhodopsin and do not interact with calmodulin. GRK4-alpha and GRK4-gamma phosphorylate DRD3. Phosphorylates ADRB2	chr4:2993963 c.187G>C(E3)	p.63,D>H	0	0.968

Table S3 - List of 489 non-synonymous and frame-shifting variants which were predicted to be damaging (X: termination codon).

Gene	Uniprot function	Genome position (hg19)	AA position & change	SIFT	Polyphen-2
CHRNB3	Neuronal acetylcholine receptor subunit beta-3,After binding acetylcholine, the AChR responds by an extensive change in conformation that affects all subunits and leads to opening of an ion-conducting channel across the plasma membrane.	chr8:42586923 c.473C>G(E5)	p.158,T>R	0.06	0.976
KIAA092	Transmembrane protein 131-like, Isoform 1: Membrane-associated form that antagonizes canonical Wnt signaling by triggering lysosome-dependent degradation of Wnt-activated LRP6. Regulates thymocyte proliferation.	chr4:154556599 c.4433C>T(E34)	p.1478,A>V	0.3	0.062
PCNXL4	Pecanex-like protein 4,	chr14:60582768 c.866G>A(E5)	p.289,R>K	0.33	0.005
KRT36	Keratin, type I cuticular Ha6, structural constituent of epidermis	chr17:39645881 c.236C>A(E1)	p.79,G>V	0.03	0.999
KRT37	Keratin, type I cuticular Ha7, structural molecule activity	chr17:39580364 c.412C>G(E1)	p.138,E>Q	0.03	0.921
CKMT1	Creatine kinase U-type, mitochondrial, Reversibly catalyzes the transfer of phosphate between ATP and various phosphogens (e.g. creatine phosphate). Creatine kinase isoenzymes play a central role in energy transduction in tissues with large, fluctuating energy demands, such as skeletal muscle, heart, brain and spermatozoa.	chr15:43991225 c.1192C>T(E10)	p.398,R>W	0	0.898
NOP14	Nucleolar complex protein 14, Involved in nucleolar processing of pre-18S ribosomal RNA. Has a role in the nuclear export of 40S pre-ribosomal subunit to the cytoplasm.	chr4:2941266 c.2306C>T(E16)	p.769,R>Q	0.21	0.105
LGALS9	Galectin-9, Binds galactosides . Has high affinity for the Forssman pentasaccharide. Ligand for HAVCR2/TIM3 . Binding to HAVCR2 induces T-helper type 1 lymphocyte (Th1) death. Also stimulates bactericidal activity in infected macrophages by causing macrophage activation and IL1B secretion which restricts intracellular bacterial growth (By similarity). Ligand for P4HB; the interaction retains P4HB at the cell surface of	chr17:25974442 c.809G>A(E9)	p.270,R>H	0.05	0.056

Table S3 - List of 489 non-synonymous and frame-shifting variants which were predicted to be damaging (X: termination codon).

Gene	Uniprot function	Genome position (hg19)	AA position & change	SIFT	Polyphen-2
	Th2 T-helper cells, increasing disulfide reductase activity at the plasma membrane, altering the plasma membrane redox state and enhancing cell migration . Ligand for CD44; the interaction enhances binding of SMAD3 to the FOXP3 promoter, leading to up-regulation of FOXP3 expression and increased induced regulatory T (iTreg) cell stability and suppressive function (By similarity). Promotes ability of mesenchymal stromal cells to suppress T-cell proliferation. Expands regulatory T-cells and induces cytotoxic T-cell apoptosis following virus infection . Activates ERK1/2 phosphorylation inducing cytokine (IL-6, IL-8, IL-12) and chemokine (CCL2) production in mast and dendritic cells . Inhibits degranulation and induces apoptosis of mast cells. Induces maturation and migration of dendritic cells. Inhibits natural killer (NK) cell function. Can transform NK cell phenotype from peripheral to decidual during pregnancy. Astrocyte derived galectin-9 enhances microglial TNF production (By similarity). May play a role in thymocyte-epithelial interactions relevant to the biology of the thymus. May provide the molecular basis for urate flux across cell membranes, allowing urate that is formed during purine metabolism to efflux from cells and serving as an electrogenic transporter that plays an important role in renal and gastrointestinal urate excretion (By similarity). Highly selective to the anion urate (By similarity).				
STEAP1	STEAP family member 1B	chr7:22478220 c.917A>G(E5)	p.306,I>T	0	0
PTPN13	Tyrosine-protein phosphatase non-receptor type 13, Tyrosine phosphatase which regulates negatively FAS-induced apoptosis and NGFR-mediated pro-apoptotic signaling	chr4:87622759 c.1000T>A(E7) p.334,S>T		0.17	0.001

Table S3 - List of 489 non-synonymous and frame-shifting variants which were predicted to be damaging (X: termination codon).

Gene	Uniprot function	Genome position (hg19)	AA position & change	SIFT	Polyphen-2
	(PubMed:15611135). May regulate phosphoinositide 3-kinase (PI3K) signaling through dephosphorylation of PIK3R2				
RHBDF1	Inactive rhomboid protein 1, Rhomboid protease-like protein which has no protease activity but regulates the secretion of several ligands of the epidermal growth factor receptor. Indirectly activates the epidermal growth factor receptor signaling pathway and may thereby regulate sleep, cell survival, proliferation and migration.	chr16:112775 c.793G>A(E6)	p.265,R>W	0	0.832
TM2D2	TM2 domain-containing protein 2,	chr8:38853759 c.200T>C(E1)	p.67,H>R	0.36	0
AKR1E2	1,5-anhydro-D-fructose reductase, Catalyzes the NADPH-dependent reduction of 1,5-anhydro-D-fructose (AF) to 1,5-anhydro-D-glucitol. Can also catalyze the reduction of various aldehydes and quinones (By similarity). Has low NADPH-dependent reductase activity towards 9,10-phenanthrenequinone (in vitro).	chr10:4875664 IVS3+6G>A splicing site	splicing region	_	_
AGBL2	Cytosolic carboxypeptidase 2, Metallocarboxypeptidase that mediates deglutamylation of target proteins. Catalyzes the deglutamylation of polyglutamate side chains generated by post-translational polyglutamylation in proteins such as tubulins. Also removes gene-encoded polyglutamates from the carboxy-terminus of target proteins such as MYLK. Does not show detyrosinase or deglycylase activities from the carboxy-terminus of tubulin.	chr11:47711820 c.1439A>G(E10)	p.480,L>P	0.01	0.999
C2orf40	Augurin, Probable hormone that may induce senescence of oligodendrocyte and neural precursor cells, characterized by G1 arrest, RB1 dephosphorylation and accelerated CCND1 and CCND3 proteasomal degradation.	chr2:106690414 c.200G>A(E3)	p.67,R>H	0	0.942

Table S3 - List of 489 non-synonymous and frame-shifting variants which were predicted to be damaging (X: termination codon).

Gene	Uniprot function	Genome position (hg19)	AA position & change	SIFT	Polyphen-2
POLQ	DNA polymerase theta, DNA polymerase that promotes microhomology-mediated end-joining (MMEJ), an alternative non-homologous end-joining (NHEJ) machinery triggered in response to double-strand breaks in DNA. MMEJ is an error-prone repair pathway that produces deletions of sequences from the strand being repaired and promotes genomic rearrangements, such as telomere fusions, some of them leading to cellular transformation. POLQ acts as an inhibitor of homology-recombination repair (HR) pathway by limiting RAD51 accumulation at resected ends). POLQ-mediated MMEJ may be required to promote the survival of cells with a compromised HR repair pathway, thereby preventing genomic havoc by resolving unrepaired lesions (By similarity). The polymerase acts by binding directly the 2 ends of resected double-strand breaks, allowing microhomologous sequences in the overhangs to form base pairs. It then extends each strand from the base-paired region using the opposing overhang as a template. Requires partially resected DNA containing 2 to 6 base pairs of microhomology to perform MMEJ . The polymerase activity is highly promiscuous: unlike most polymerases, promotes extension of ssDNA and partial ssDNA (pssDNA) substrates. Also exhibits low-fidelity DNA synthesis, translesion synthesis and lyase activity, and it is implicated in interstrand-cross-link repair, base excision repair and DNA end-joining. Involved in somatic hypermutation of immunoglobulin genes, a process that requires the activity of DNA polymerases to ultimately introduce mutations at both A/T and C/G base pairs (By similarity)	chr3:121208731 c.3047A>G(E16)	p.1016,V>A	0.17	0.006

Table S3 - List of 489 non-synonymous and frame-shifting variants which were predicted to be damaging (X: termination codon).

Gene	Uniprot function	Genome position (hg19)	AA position & change	SIFT	Polyphen-2
MRPL55	39S ribosomal protein L55, mitochondrial,	chr1:228294567 c.281C>T(E4)	p.94,R>Q	0.05	0.907
PBRM1	Protein polybromo-1, Involved in transcriptional activation and repression of select genes by chromatin remodeling (alteration of DNA-nucleosome topology). Acts as a negative regulator of cell proliferation.	chr3:52584526 c.4487C>T(E29)	p.1496,R>Q	0	0.942
CRAMP1	Protein cramped-like, DNA and chromatin binding	chr16:1717404 IVS16+3A>G splicing cite			
EPHB3	Ephrin type-B receptor 3, Receptor tyrosine kinase which binds promiscuously transmembrane ephrin-B family ligands residing on adjacent cells, leading to contact-dependent bidirectional signaling into neighboring cells. The signaling pathway downstream of the receptor is referred to as forward signaling while the signaling pathway downstream of the ephrin ligand is referred to as reverse signaling. Generally has an overlapping and redundant function with EPHB2. Like EPHB2, functions in axon guidance during development regulating for instance the neurons forming the corpus callosum and the anterior commissure, 2 major interhemispheric connections between the temporal lobes of the cerebral cortex. In addition to its role in axon guidance plays also an important redundant role with other ephrin-B receptors in development and maturation of dendritic spines and the formation of excitatory synapses. Controls other aspects of development through regulation of cell migration and positioning. This includes angiogenesis, palate development and thymic epithelium development for instance. Forward and reverse signaling through the EFNB2/EPHB3 complex also regulate migration and adhesion of cells that tubularize the urethra and septate the cloaca. Finally, plays an important role in intestinal epithelium	chr3:184294704 c.1087C>T(E5)	p.363,R>W	0.04	0.932

Table S3 - List of 489 non-synonymous and frame-shifting variants which were predicted to be damaging (X: termination codon).

Gene	Uniprot function	Genome position (hg19)	AA position & change	SIFT	Polyphen-2
	differentiation segregating progenitor from differentiated cells in the crypt				
ACR	Acrosin, Acrosin is the major protease of mammalian spermatozoa. It is a serine protease of trypsin-like cleavage specificity, it is synthesized in a zymogen form, proacrosin and stored in the acrosome.	chr22:51176734 c.71C>T(E1)	p.24,T>M	0.04	0.982
LRCH4	Leucine-rich repeat and calponin homology domain-containing protein 4	chr7:100175477 c.1007C>T(E8)	p.336,R>Q	0	0.99
PDE4DIP	Myomegalin, May function as an anchor sequestering components of the cAMP-dependent pathway to Golgi and/or centrosomes.	chr1:144852390 c.7053C>T(E44)	p.2351,W>X(12)	_	_
		chr1:144886092 IVS26+5C>T splicing site	splicing region	_	_
NEK9	Serine/threonine-protein kinase Nek9, Pleiotropic regulator of mitotic progression, participating in the control of spindle dynamics and chromosome separation. Phosphorylates different histones, myelin basic protein, beta-casein, and BICD2. Phosphorylates histone H3 on serine and threonine residues and beta-casein on serine residues. Important for G1/S transition and S phase progression. Phosphorylates NEK6 and NEK7 and stimulates their activity by releasing the autoinhibitory functions of Tyr-108 and Tyr-97 respectively.	chr14:75555305 c.2482G>T(E20)	p.828,P>T	0.06	0.053
TMEM18	Transmembrane protein 184A, Acts as a heparin receptor in vascular cells (By similarity). May be involved in vesicle transport in exocrine cells and Sertoli cells (By similarity).	chr7:1586653-1586654 c.1177(E9)-c.1176(E9)	p.392-393 -/GG	_	_
TANC2	Protein TANC2, in utero embryonic development	chr17:61176593 c.197T>C(E3)	p.66,L>P	0.1	0.18
UBR7	Putative E3 ubiquitin-protein ligase UBR7, E3 ubiquitin-protein ligase which is a component of the N-end rule pathway. Recognizes and binds to proteins bearing specific N-terminal residues that are destabilizing according to the N-end rule,	chr14:93685612 c.865C>G(E8)	p.289,Q>E	0.16	0.001

Table S3 - List of 489 non-synonymous and frame-shifting variants which were predicted to be damaging (X: termination codon).

Gene	Uniprot function	Genome position (hg19)	AA position & change	SIFT	Polyphen-2
	leading to their ubiquitination and subsequent degradation (By similarity).				
NKTR	NK-tumor recognition protein, Component of a putative tumor-recognition complex. Involved in the function of NK cells.	chr3:42676140 c.965A>G(E11)	p.322,Q>R	0.01	0
DCTN4	Dynactin subunit 4, Could have a dual role in dynein targeting and in ACTR1A/Arp1 subunit of dynactin pointed-end capping. Could be involved in ACTR1A pointed-end binding and in additional roles in linking dynein and dynactin to the cortical cytoskeleton.	chr5:150102470 c.928C>T(E10)	p.310,V>I	0.26	0.007
FAM86B	Putative protein N-methyltransferase FAM86B2,	chr8:12291593 c.127C>A(E2)	p.43,D>Y	0.01	0.288
TMEM26	Transmembrane protein 26	chr10:63188718 c.571A>C(E4)	p.191,F>V	0.02	0.999
NUDT11	Diphosphoinositol polyphosphate phosphohydrolase 3-beta, Cleaves a beta-phosphate from the diphosphate groups in PP-InsP5 (diphosphoinositol pentakisphosphate), suggesting that it may play a role in signal transduction. Also able to catalyze the hydrolysis of dinucleoside oligophosphates, with Ap6A and Ap5A being the preferred substrates. The major reaction products are ADP and p4a from Ap6A and ADP and ATP from Ap5A. Also able to hydrolyze 5-phosphoribose 1-diphosphate.	chrX:51239296-51239309 c.1(E1)-m.151(E1):	_	_	_
VTI1B	Vesicle transport through interaction with t-SNAREs homolog 1B, V-SNARE that mediates vesicle transport pathways through interactions with t-SNAREs on the target membrane. These interactions are proposed to mediate aspects of the specificity of vesicle trafficking and to promote fusion of the lipid bilayers. May be concerned with increased secretion of cytokines associated with cellular senescence.	chr14:68123303 c.370G>A(E4)	p.124,R>W	0.14	0.002

Table S3 - List of 489 non-synonymous and frame-shifting variants which were predicted to be damaging (X: termination codon).

Gene	Uniprot function	Genome position (hg19)	AA position & change	SIFT	Polyphen-2
UNC5CL	UNC5C-like protein, Inhibits NF-kappa-B-dependent transcription by impairing NF-kappa-B binding to its targets.	chr6:41000676 c.896T>A(E4)	p.299,D>V	0.08	0.314
NUDT18	8-oxo-dGDP phosphatase NUDT18, Mediates the hydrolyzis of oxidized nucleoside diphosphate derivatives. Hydrolyzes 8-oxo-7,8-dihydroguanine (8-oxo-Gua)-containing deoxyribo- and ribonucleoside diphosphates to the monophosphates. Hydrolyzes 8-oxo-dGDP and 8-oxo-GDP with the same efficiencies. Hydrolyzes also 8-OH-dADP and 2-OH-dADP. Exhibited no or minimal hydrolyzis activity against 8-oxo-dGTP, 8-oxo-GTP, dGTP, GTP, dGDP and GDP. Probably removes oxidized guanine nucleotides from both the DNA and RNA precursor pools.	chr8:21966703-21966708, c.111(E1)-c.106(E1	_	_	_
HCAR3	Hydroxycarboxylic acid receptor 3, Receptor for 3-OH-octanoid acid mediates a negative feedback regulation of adipocyte lipolysis to counteract prolipolytic influences under conditions of physiological or pathological increases in beta-oxidation rates. Acts as a low affinity receptor for nicotinic acid. This pharmacological effect requires nicotinic acid doses that are much higher than those provided by a normal diet.	chr12:123201233 c.52A>G(E1)	p.18,C>R	0.01	0.998
COL12A	Collagen alpha-1(XII) chain, Type XII collagen interacts with type I collagen-containing fibrils, the COL1 domain could be associated with the surface of the fibrils, and the COL2 and NC3 domains may be localized in the perifibrillar matrix.	chr6:75862112 c.3652C>T(E18)	p.1218,V>M	0	0.668
VPS13D	Vacuolar protein sorting-associated protein 13D, protein retention in Golgi apparatus, protein targeting to vacuole	chr1:12398297 c.8561T>G(E40)	p.2854,L>R	_	0.587

Table S3 - List of 489 non-synonymous and frame-shifting variants which were predicted to be damaging (X: termination codon).

Gene	Uniprot function	Genome position (hg19)	AA position & change	SIFT	Polyphen-2
BAK1	BRASSINOSTEROID INSENSITIVE 1-associated receptor kinase 1, Dual specificity kinase acting on both serine/threonine- and tyrosine-containing substrates. Controls the expression of genes associated with innate immunity in the absence of pathogens or elicitors. Involved in brassinosteroid (BR) signal transduction. Phosphorylates BRI1. May be involved in changing the equilibrium between plasma membrane-located BRI1 homodimers and endocytosed BRI1-BAK1 heterodimers. Interaction with MSBP1 stimulates the endocytosis of BAK1 and suppresses brassinosteroid signaling. Acts in pathogen-associated molecular pattern (PAMP)-triggered immunity (PTI) via its interactions with FLS2 and EFR, and the phosphorylation of BIK1. Involved in programmed cell death (PCD) control. Positively regulates the BR-dependent plant growth pathway and negatively regulates the BR-independent cell-death pathway. Phosphorylates BIR2 and thus promotes interaction with BIR2. This interaction prevents interaction with FLS2 in the absence of pathogen-associated molecular patterns (PAMP)	chr6:33543607 c.169C>T(E3) p.57,D>N		0.06	0.017
CD48	CD48 antigen, Ligand for CD2. Might facilitate interaction between activated lymphocytes. Probably involved in regulating T-cell activation.	chr1:160654857 c.205A>G(E2)	p.69,W>R	0.05	0.941
DNAJB1	DnaJ homolog subfamily B member 12, Chaperone	chr10:74096378 c.1012C>T(E7)	p.338,E>K	0	0.993
PLEKHN	Pleckstrin homology domain-containing family N member 1	chr1:906512 c.668G>A(E7)	p.223,R>Q	0.35	0.031

Table S3 - List of 489 non-synonymous and frame-shifting variants which were predicted to be damaging (X: termination codon).

Gene	Uniprot function	Genome position (hg19)	AA position & change	SIFT	Polyphen-2
DSC1	Desmocollin-1, Component of intercellular desmosome junctions. Involved in the interaction of plaque proteins and intermediate filaments mediating cell-cell adhesion. May contribute to epidermal cell positioning (stratification) by mediating differential adhesiveness between cells that express different isoforms. Linked to the keratinization of epithelial tissues.	chr18:28713893 c.2077C>T(E13)	p.693,A>T	0.02	0.992
SNX32	Sorting nexin-32, May be involved in several stages of intracellular trafficking.	chr11:65618258 c.535G>A(E6)	p.179,G>R	0.11	0.921
SDR42E1	Short-chain dehydrogenase/reductase family 42E member 1	chr16:82032960 c.938C>T(E3)	p.313,R>H	0.03	0.202
SCRN1	Secernin-1, Regulates exocytosis in mast cells. Increases both the extent of secretion and the sensitivity of mast cells to stimulation with calcium (By similarity).	chr7:30008637 c.47C>T(E2)	p.16,R>H	0.25	0.01
NES	Nestin, Required for brain and eye development. Promotes the disassembly of phosphorylated vimentin intermediate filaments (IF) during mitosis and may play a role in the trafficking and distribution of IF proteins and other cellular factors to daughter cells during progenitor cell division. Required for survival, renewal and mitogen-stimulated proliferation of neural progenitor cells (By similarity).	chr1:156641609 c.2371G>T(E4)	p.791,L>I	0.03	0.151
BGLAP	Osteocalcin,Constitutes 1-2% of the total bone protein. It binds strongly to apatite and calcium.	chr1:156212567 c.118C>A(E3)	p.40,Q>K	0.08	0.005
CHD5	Chromodomain-helicase-DNA-binding protein 5, hromatin-remodeling protein that binds DNA through histones and regulates gene transcription. May specifically recognize and bind trimethylated 'Lys-27' (H3K27me3) and non-methylated 'Lys-4' of histone H3. Plays a role in the development of the nervous system by activating the expression of genes promoting neuron terminal differentiation. In parallel, it may also positively regulate the	chr1:6184131 c.4576T>A(E31)	p.1526,T>S	0.08	0.016

Table S3 - List of 489 non-synonymous and frame-shifting variants which were predicted to be damaging (X: termination codon).

Gene	Uniprot function	Genome position (hg19)	AA position & change	SIFT	Polyphen-2
	trimethylation of histone H3 at 'Lys-27' thereby specifically repressing genes that promote the differentiation into non-neuronal cell lineages. Tumor suppressor, it regulates the expression of genes involved in cell proliferation and differentiation. Downstream activated genes may include CDKN2A that positively regulates the p53/TP53 pathway, which in turn, prevents cell proliferation. In spermatogenesis, it probably regulates histone hyperacetylation and the replacement of histones by transition proteins in chromatin, a crucial step in the condensation of spermatid chromatin and the production of functional spermatozoa.				
HSPA4	Heat shock 70 kDa protein 4, ATP binding	chr5:132440100 c.2495A>G(E19)	p.832,K>R	0.05	0.079
GPSM1	G-protein-signaling modulator 1, Guanine nucleotide dissociation inhibitor (GDI) which functions as a receptor-independent activator of heterotrimeric G-protein signaling. Keeps G(i/o) alpha subunit in its GDP-bound form thus uncoupling heterotrimeric G-proteins signaling from G protein-coupled receptors. Controls spindle orientation and asymmetric cell fate of cerebral cortical progenitors. May also be involved in macroautophagy in intestinal cells. May play a role in drug addiction.	chr9:139235482-139235482 c.1239(E9):C dele	p.414,P>Pfs56	_	_
ALDH1A	Retinal dehydrogenase 1, Binds free retinal and cellular retinol-binding protein-bound retinal. Can convert/oxidize retinaldehyde to retinoic acid (By similarity).	chr9:75540504 c.529T>A(E6)	p.177,I>F	0.09	0.008
TMED6	Transmembrane emp24 domain-containing protein 6	chr16:69381824 c.356C>G(E3)	p.119,C>S	0	0.976
UPP2	Uridine phosphorylase 2, Catalyzes the reversible phosphorylytic cleavage of uridine and deoxyuridine to uracil and ribose- or deoxyribose-1-phosphate. The produced molecules are then	chr2:158958551-158958552 c.147(E3)-c.148(	p.-/X	_	_

Table S3 - List of 489 non-synonymous and frame-shifting variants which were predicted to be damaging (X: termination codon).

Gene	Uniprot function	Genome position (hg19)	AA position & change	SIFT	Polyphen-2
	utilized as carbon and energy sources or in the rescue of pyrimidine bases for nucleotide synthesis. Shows substrate specificity and accept uridine, deoxyuridine, and thymidine as well as the two pyrimidine nucleoside analogs 5-fluorouridine and 5-fluoro-2(')-deoxyuridine as substrates.				
BTN2A1	Butyrophilin subfamily 2 member A1, lipid metabolic process	chr6:26459996 c.187C>T(E2)	p.63,R>C	0.22	0.014
GALNT4	Polypeptide N-acetylgalactosaminyltransferase 4, Catalyzes the initial reaction in O-linked oligosaccharide biosynthesis, the transfer of an N-acetyl-D-galactosamine residue to a serine or threonine residue on the protein receptor. Has a highest activity toward Muc7, EA2 and Muc2, with a lowest activity than GALNT2. Glycosylates 'Thr-57' of SELPLG.	chr12:89916672 c.1655C>T(E1)	p.552,R>Q	0.14	0.832
ANKRD3	Ankyrin repeat domain-containing protein 34C	chr15:79586159 c.533A>G(E2)	p.178,D>G	0.18	0.008
		chr15:79586669 c.1043C>A(E2)	p.348,T>N	0.16	0.766
IRX2	Iroquois-class homeodomain protein IRX-2, sequence-specific DNA binding	chr5:2749874 c.277C>G(E2)	p.93,G>R	0	1
GALNT5	Polypeptide N-acetylgalactosaminyltransferase 5, Catalyzes the initial reaction in O-linked oligosaccharide biosynthesis, the transfer of an N-acetyl-D-galactosamine residue to a serine or threonine residue on the protein receptor. Has activity toward EA2 peptide substrate, but has a weak activity toward Muc2 or Muc1b substrates (By similarity)	chr2:158140808 c.1469T>C(E2)	p.490,L>P	0.02	0.866
ADAMT	A disintegrin and metalloproteinase with thrombospondin motifs 14, Has a aminoprocollagen type I activity processing activity in the absence of ADAMTS2. Seems to be synthesized as a latent enzyme that requires activation to display aminoprocollagen peptidase activity.	chr10:72462119 c.574C>T(E3)	p.192,R>W	0	0.715

Table S3 - List of 489 non-synonymous and frame-shifting variants which were predicted to be damaging (X: termination codon).

Gene	Uniprot function	Genome position (hg19)	AA position & change	SIFT	Polyphen-2
C2orf73	Uncharacterized protein C2orf73	chr2:54561964 c.37A>C(E2)	p.13,I>L	0.05	0.009
ZFPM1	Zinc finger protein ZFPM1, Transcription regulator that plays an essential role in erythroid and megakaryocytic cell differentiation. Essential cofactor that acts via the formation of a heterodimer with transcription factors of the GATA family GATA1, GATA2 and GATA3. Such heterodimer can both activate or repress transcriptional activity, depending on the cell and promoter context. The heterodimer formed with GATA proteins is essential to activate expression of genes such as NFE2, ITGA2B, alpha- and beta-globin, while it represses expression of KLF1. May be involved in regulation of some genes in gonads. May also be involved in cardiac development, in a non-redundant way with ZFPM2/FOG2 (By similarity).	chr16:88599697-88599705 c.1331(E10)-c.1339(E10): AGCCTCTGG deleted		–	–
FAM120	Constitutive coactivator of PPAR-gamma-like protein 2, poly(A) RNA binding	chrX:54117740 IVS11+5C>A splicing site	splicing region	–	–
SH2D3A	SH2 domain-containing protein 3A, May play a role in JNK activation.	chr19:6755198 c.625C>T(E5)	p.209,A>T	0.04	0.101
NCOA1	Nuclear receptor coactivator 1, Nuclear receptor coactivator that directly binds nuclear receptors and stimulates the transcriptional activities in a hormone-dependent fashion. Involved in the coactivation of different nuclear receptors, such as for steroids (PGR, GR and ER), retinoids (RXRs), thyroid hormone (TRs) and prostanoids (PPARs). Also involved in coactivation mediated by STAT3, STAT5A, STAT5B and STAT6 transcription factors. Displays histone acetyltransferase activity toward H3 and H4; the relevance of such activity remains however unclear. Plays a central role in creating multisubunit coactivator complexes that act via remodeling of chromatin, and possibly acts by participating in both chromatin remodeling and	chr2:24974958 c.3814C>T(E18)	p.1272,P>S	0.04	0.237

Table S3 - List of 489 non-synonymous and frame-shifting variants which were predicted to be damaging (X: termination codon).

Gene	Uniprot function	Genome position (hg19)	AA position & change	SIFT	Polyphen-2
	recruitment of general transcription factors. Required with NCOA2 to control energy balance between white and brown adipose tissues. Required for mediating steroid hormone response. Isoform 2 has a higher thyroid hormone-dependent transactivation activity than isoform 1 and isoform 3.				
NRDE2	Protein NRDE2 homolog,	chr14:90756862 c.1932C>G(E10)	p.644,L>F	0	0.974
LYPD6B	Ly6/PLAUR domain-containing protein 6B,	chr2:150069497 IVS5-9C>G splicing site	_	_	_
HLTF	Helicase-like transcription factor, Has both helicase and E3 ubiquitin ligase activities. Possesses intrinsic ATP-dependent nucleosome-remodeling activity; This activity may be required for transcriptional activation or repression of specific target promoters (By similarity). These may include the SERPINE1 and HIV-1 promoters and the SV40 enhancer, to which this protein can bind directly. Plays a role in error-free postreplication repair (PRR) of damaged DNA and maintains genomic stability through acting as a ubiquitin ligase for 'Lys-63'-linked polyubiquitination of chromatin-bound PCNA.	chr3:148786085 c.932T>C(E8)	p.311,N>S	0.03	0.474
CNTN5	Contactin-5, Contactins mediate cell surface interactions during nervous system development. Has some neurite outgrowth-promoting activity in the cerebral cortical neurons but not in hippocampal neurons. Probably involved in neuronal activity in the auditory system (By similarity).	chr11:100221524 c.3122A>G(E23)	p.1041,Y>C	0.01	1

Table S3 - List of 489 non-synonymous and frame-shifting variants which were predicted to be damaging (X: termination codon).

Gene	Uniprot function	Genome position (hg19)	AA position & change	SIFT	Polyphen-2
TRPM8	Transient receptor potential cation channel subfamily M member 8, Receptor-activated non-selective cation channel involved in detection of sensations such as coolness, by being activated by cold temperature below 25 degrees Celsius. Activated by icilin, eucalyptol, menthol, cold and modulation of intracellular pH. Involved in menthol sensation. Permeable for monovalent cations sodium, potassium, and cesium and divalent cation calcium. Temperature sensing is tightly linked to voltage-dependent gating. Activated upon depolarization, changes in temperature resulting in graded shifts of its voltage-dependent activation curves. The chemical agonist menthol functions as a gating modifier, shifting activation curves towards physiological membrane potentials. Temperature sensitivity arises from a tenfold difference in the activation energies associated with voltage-dependent opening and closing. In prostate cancer cells, shows strong inward rectification and high calcium selectivity in contrast to its behavior in normal cells which is characterized by outward rectification and poor cationic selectivity. Plays a role in prostate cancer cell migration. Isoform 2 and isoform 3 negatively regulate menthol- and cold-induced channel activity by stabilizing the closed state of the channel.	chr2:234871987 c.1715A>C(E13)	p.572,N>T	0	0.905
AHNAK	Neuroblast differentiation-associated protein AHNAK, May be required for neuronal cell differentiation.	chr11:62299985 c.1904G>A(E5)	p.635,T>M	_	0.781
GPC1	Glypican-1, Cell surface proteoglycan that bears heparan sulfate. Binds, via the heparan sulfate side chains, alpha-4 (V) collagen and participates in Schwann cell myelination (By similarity). May act as a catalyst in increasing the rate of conversion of	chr2:241404342 c.1084G>A(E6)	p.362,G>S	0.17	0.124

Table S3 - List of 489 non-synonymous and frame-shifting variants which were predicted to be damaging (X: termination codon).

Gene	Uniprot function	Genome position (hg19)	AA position & change	SIFT	Polyphen-2
	prion protein PRPN(C) to PRNP(Sc) via associating (via the heparan sulfate side chains) with both forms of PRPN, targeting them to lipid rafts and facilitating their interaction. Required for proper skeletal muscle differentiation by sequestering FGF2 in lipid rafts preventing its binding to receptors (FGFRs) and inhibiting the FGF-mediated signaling				
CTBS	Di-N-acetylchitobiase, Involved in the degradation of asparagine-linked glycoproteins. Hydrolyze of N-acetyl-beta-D-glucosamine (1-4)N-acetylglucosamine chitobiose core from the reducing end of the bond, it requires prior cleavage by glycosylasparaginase.	chr1:85028969 c.928C>A(E6)	p.310,D>Y	0.03	0.063
SPINT4	Kunitz-type protease inhibitor 4, serine-type endopeptidase inhibitor activity	chr20:44352519 c.116A>T(E2)	p.39,D>V	0.1	0.355
NBPF1	Neuroblastoma breakpoint family member 1	chr1:16903912 c.1804T>A(E18)	p.602,N>Y	0.17	0.001
		chr1:16902884 c.1997T>C(E19)	p.666,N>S	0.14	0.79
FRMD8	FERM domain-containing protein 8	chr11:65156835 c.89C>G(E3)	p.30,A>G	0.21	0.698
APLF	Aprataxin and PNK-like factor, Nuclease involved in single-strand and double-strand DNA break repair. Recruited to sites of DNA damage through interaction with poly(ADP-ribose), a polymeric post-translational modification synthesized transiently at sites of chromosomal damage to accelerate DNA strand break repair reactions. Displays apurinic-apyrimidinic (AP) endonuclease and 3'-5' exonuclease activities in vitro. Also able to introduce nicks at hydroxyuracil and other types of pyrimidine base damage.	chr2:68729906 c.212A>T(E3)	p.71,Q>L	0.01	0.629
MS4A14	Membrane-spanning 4-domains subfamily A member 14, May be involved in signal transduction as a component of a multimeric receptor complex.	chr11:60183546 c.1054G>C(E4)	p.352,D>H	0.03	0.056
PDZD4	PDZ domain-containing protein 4,	chrX:153069980 c.1138C>T(E8)	p.380,A>T	0.45	0.101

Table S3 - List of 489 non-synonymous and frame-shifting variants which were predicted to be damaging (X: termination codon).

Gene	Uniprot function	Genome position (hg19)	AA position & change	SIFT	Polyphen-2
OR52E8	Olfactory receptor 52E8, Odorant receptor.	chr11:5878887 c.46A>G(E1)	p.16,S>P	0.17	0.786
OR2T27	Olfactory receptor 2T27, Odorant receptor.	chr1:248813297 c.889C>T(E1)	p.297,V>I	0.02	0.799
SARM1	Sterile alpha and TIR motif-containing protein 1,Negative regulator of MYD88- and TRIF-dependent toll-like receptor signaling pathway which plays a pivotal role in activating axonal degeneration following injury. Promotes Wallerian degeneration an injury-induced axonal death pathway which involves degeneration of an axon distal to the injury site. Can activate neuronal death in response to stress. Regulates dendritic arborization through the MAPK4-JNK pathway. Involved in innate immune response. Inhibits both TICAM1/TRIF- and MYD88-dependent activation of JUN/AP-1, TRIF-dependent activation of NF-kappa-B and IRF3, and the phosphorylation of MAPK14/p38	chr17:26699196-26699206 c.143(E1)-c.153(E1):CGGGCCCGCGA deleted G		–	–
LMCD1	LIM and cysteine-rich domains protein 1, Transcriptional cofactor that restricts GATA6 function by inhibiting DNA-binding, resulting in repression of GATA6 transcriptional activation of downstream target genes. Represses GATA6-mediated trans activation of lung- and cardiac tissue-specific promoters. Inhibits DNA-binding by GATA4 and GATA1 to the cTNC promoter (By similarity). Plays a critical role in the development of cardiac hypertrophy via activation of calcineurin/nuclear factor of activated T-cells signaling pathway.	chr3:8590329 c.463C>T(E4)	p.155,R>C	0	0.908
ACMSD	2-amino-3-carboxymuconate-6-semialdehyde decarboxylase, Converts alpha-amino-beta-carboxymuconate-epsilon-semialdehyde (ACMS) to alpha-aminomuconate semialdehyde (AMS). ACMS can be converted non-enzymatically to quinolate (QA), a key precursor of NAD, and a potent endogenous excitotoxin of neuronal cells	chr2:135616891 c.163G>C(E3)	p.55,D>H	0.05	0.741

Table S3 - List of 489 non-synonymous and frame-shifting variants which were predicted to be damaging (X: termination codon).

Gene	Uniprot function	Genome position (hg19)	AA position & change	SIFT	Polyphen-2
	which is implicated in the pathogenesis of various neurodegenerative disorders. In the presence of ACMSD, ACMS is converted to AMS, a benign catabolite. ACMSD ultimately controls the metabolic fate of tryptophan catabolism along the kynurenine pathway.				
PRB4	Basic salivary proline-rich protein 4,	chr12:11461414 c.503T>C(E3)	p.168,Q>R	0.04	0
MYO1C	Unconventional myosin-Ic,Myosins are actin-based motor molecules with ATPase activity. Unconventional myosins serve in intracellular movements. Their highly divergent tails are presumed to bind to membranous compartments, which would be moved relative to actin filaments. Involved in glucose transporter recycling in response to insulin by regulating movement of intracellular GLUT4-containing vesicles to the plasma membrane. Component of the hair cell's (the sensory cells of the inner ear) adaptation-motor complex. Acts as a mediator of adaptation of mechanoelectrical transduction in stereocilia of vestibular hair cells. Binds phosphoinositides and links the actin cytoskeleton to cellular membranes.Isoform 3 is involved in regulation of transcription. Associated with transcriptional active ribosomal genes. Appears to cooperate with the WICH chromatin-remodeling complex to facilitate transcription. Necessary for the formation of the first phosphodiester bond during transcription initiation (By similarity)	chr17:1375328 IVS18-10G>C splicing site			
ACNA2	Voltage-dependent calcium channel subunit alpha-2/delta-1,The alpha-2/delta subunit of voltage-dependent calcium channels regulates calcium current density and activation/inactivation kinetics of the calcium channel. Plays an important role in excitation-contraction coupling (By similarity).	chr7:81611914 c.1924C>G(E24)	p.642,E>Q	0.1	0.557
ANGPTL	Angiopoietin-related protein 5	chr11:101762206 c.971C>T(E9)	p.324,C>Y	0	0.974

Table S3 - List of 489 non-synonymous and frame-shifting variants which were predicted to be damaging (X: termination codon).

Gene	Uniprot function	Genome position (hg19)	AA position & change	SIFT	Polyphen-2
FGD5	FYVE, RhoGEF and PH domain-containing protein 5,Activates CDC42, a member of the Ras-like family of Rho- and Rac proteins, by exchanging bound GDP for free GTP. Mediates VEGF-induced CDC42 activation. May regulate proangiogenic action of VEGF in vascular endothelial cells, including network formation, directional movement and proliferation. May play a role in regulating the actin cytoskeleton and cell shape.	chr3:14862274 c.1696C>G(E1)	p.566,P>A	0.1	0.111
OR2T34	Olfactory receptor 2T34, Odorant receptor.	chr1:248737754 c.305C>T(E1)	p.102,C>Y	0	0.997
OR2T35	Olfactory receptor 2T35, Odorant receptor.	chr1:248801610 c.950G>A(E1)	p.317,A>V	0.19	0
		chr1:248801611 c.949C>T(E1)	p.317,A>T	0.11	0.006
OR2T33	Olfactory receptor 2T33, Odorant receptor.	chr1:248436165 c.952T>C(E1)	p.318,R>G	0.29	0
BTN1A1	Butyrophilin subfamily 1 member A1,May function in the secretion of milk-fat droplets. May act as a specific membrane-associated receptor for the association of cytoplasmic droplets with the apical plasma membrane (By similarity). Inhibits the proliferation of CD4 and CD8 T-cells activated by anti-CD3 antibodies, T-cell metabolism and IL2 and IFNG secretion (By similarity).	chr6:26508729 c.908T>C(E7)	p.303,V>A	1	0.997
PMF1-B	Protein PMF1-BGLAP,Uncharacterized	chr1:156212567 c.557C>A(E6)	p.186,A>E	0	0.932
QSER1	Glutamine and serine-rich protein 1	chr11:32953516 c.325G>A(E4)	p.109,V>I	0.13	0.003
TTC3	E3 ubiquitin-protein ligase TTC3,E3 ubiquitin-protein ligase that mediates the ubiquitination and subsequent degradation of phosphorylated Akt (AKT1, AKT2 and AKT3) in the nucleus. Acts as a terminal regulator of Akt signaling after activation; its phosphorylation by Akt, which is a prerequisite for ubiquitin ligase activity, suggests the existence of a regulation mechanism required to control Akt levels after activation. Catalyzes the formation of 'Lys-48'-polyubiquitin chains. May play a role in neuronal differentiation inhibition	chr21:38538154 c.3638(E33):A deleted	p.1213,E>Dfs6		

Table S3 - List of 489 non-synonymous and frame-shifting variants which were predicted to be damaging (X: termination codon).

Gene	Uniprot function	Genome position (hg19)	AA position & change	SIFT	Polyphen-2
	via its interaction with CIT.				
OR2L3	Olfactory receptor 2L3, Odorant receptor.	chr1:248224690 c.707C>T(E1)	p.236,A>V	0.01	0.911
SESN2	Sestrin-2, Functions as an intracellular leucine sensor that negatively regulates the TORC1 signaling pathway through the GATOR complex. In absence of leucine, binds the GATOR subcomplex GATOR2 and prevents TORC1 signaling (PubMed:18692468, PubMed:25263562, PubMed:25457612, PubMed:26449471, PubMed:26612684, PubMed:26586190). Binding of leucine to SESN2 disrupts its interaction with GATOR2 thereby activating the TORC1 signaling pathway (PubMed:26449471, PubMed:26586190). This stress-inducible metabolic regulator also plays a role in protection against oxidative and genotoxic stresses. May negatively regulate protein translation in response to endoplasmic reticulum stress, via TORC1. May positively regulate the transcription by NFE2L2 of genes involved in the response to oxidative stress by facilitating the SQSTM1-mediated autophagic degradation of KEAP1. May also mediate TP53 inhibition of TORC1 signaling upon genotoxic stres. Has an alkylhydroperoxide reductase activity born by the N-terminal domain of the protein. Was originally reported to contribute to oxidative stress resistance by reducing PRDX1 . However, this could not be confirmed	chr1:28598287 c.259C>T(E3)	p.87,P>S	0.05	1
TMEM21	Transmembrane protein 218,May be involved in ciliary biogenesis or function.	chr11:124972113 c.25C>T(E3)	p.9,G>S	0	1
KBTBD1	Kelch repeat and BTB domain-containing protein 12	chr3:127703051 c.1802T>C(E5)	p.601,V>A	0.22	0.996
ADAMT	ADAMTS-like protein 3,metallopeptidase activity, zinc ion binding	chr15:84651796 c.3416G>A(E21)	p.1139,R>Q	0.1	0.008

Table S3 - List of 489 non-synonymous and frame-shifting variants which were predicted to be damaging (X: termination codon).

Gene	Uniprot function	Genome position (hg19)	AA position & change	SIFT	Polyphen-2
TMEM21	Transmembrane protein 214, Critical mediator, in cooperation with CASP4, of endoplasmic reticulum-stress induced apoptosis. Required or the activation of CASP4 following endoplasmic reticulum stress	chr2:27257002-27257002 c.219(E2):G deleted	p.74,E>Sfs42		
OR2L8	Olfactory receptor 2L8, Odorant receptor.	chr1:248112869 c.710A>G(E1)	p.237,Y>C	0.02	0.962
LRRC61	Leucine-rich repeat-containing protein 61	chr7:150034456 c.506G>A(E3)	p.169,G>D	0	0.999
NFE2L2	Nuclear factor erythroid 2-related factor 2, Transcription activator that binds to antioxidant response (ARE) elements in the promoter regions of target genes. Important for the coordinated up-regulation of genes in response to oxidative stress. May be involved in the transcriptional activation of genes of the beta-globin cluster by mediating enhancer activity of hypersensitive site 2 of the beta-globin locus control region.	chr2:178096634 c.649G>A(E5)	p.217,P>S	0.37	0.019
MGA2	Protein MGA2, positive regulation of transcription from RNA polymerase II promoter	chr15:42058948 c.8041G>A(E23)	p.2681,V>I	0.29	0.002
GPR50	Melatonin-related receptor,Does not bind melatonin. G-protein coupled receptor activity	chrX:150349558-150349569 c.1503(E2)-c.151	p.501-505 PTTGH>P		
TELO2	Telomere length regulation protein TEL2 homolog, Regulator of the DNA damage response (DDR). Part of the TTT complex that is required to stabilize protein levels of the phosphatidylinositol 3-kinase-related protein kinase (PIKK) family proteins. The TTT complex is involved in the cellular resistance to DNA damage stresses, like ionizing radiation (IR), ultraviolet (UV) and mitomycin C (MMC). Together with the TTT complex and HSP90 may participate in the proper folding of newly synthesized PIKKs. Promotes assembly, stabilizes and maintains the activity of mTORC1 and mTORC2 complexes, which regulate cell growth and survival in response to nutrient and hormonal signals. May be involved in telomere length regulation.	chr16:1544464 c.182C>A(E2)	p.61,P>H	0.16	0.01

Table S3 - List of 489 non-synonymous and frame-shifting variants which were predicted to be damaging (X: termination codon).

Gene	Uniprot function	Genome position (hg19)	AA position & change	SIFT	Polyphen-2
SOGA1	Regulates autophagy by playing a role in the reduction of glucose production in an adiponectin- and insulin-dependent manner.	SOGA1 chr20:35438474 c.2494C>T(E7)	p.832,D>N	0.03	0.652
BPIFA2	BPI fold-containing family A member 2, Has strong antibacterial activity against P. aeruginosa.	chr20:31757102 c.151C>G(E2)	p.51,L>V	0.17	0.07
POU2AF	POU domain class 2-associating factor 1, Transcriptional coactivator that specifically associates with either OCT1 or OCT2. It boosts the OCT1 mediated promoter activity and to a lesser extent, that of OCT2. It has no intrinsic DNA-binding activity. It recognizes the POU domains of OCT1 and OCT2. It is essential for the response of B-cells to antigens and required for the formation of germinal centers.	chr11:111229646 IVS1-3G>A splicing site			
IRS4	Insulin receptor substrate 4,Acts as an interface between multiple growth factor receptors possessing tyrosine kinase activity, such as insulin receptor, IGF1R and FGFR1, and a complex network of intracellular signaling molecules containing SH2 domains. Involved in the IGF1R mitogenic signaling pathway. Promotes the AKT1 signaling pathway and BAD phosphorylation during insulin stimulation without activation of RPS6KB1 or the inhibition of apoptosis. Interaction with GRB2 enhances insulin-stimulated mitogen-activated protein kinase activity. May be involved in nonreceptor tyrosine kinase signaling in myoblasts. Plays a pivotal role in the proliferation/differentiation of hepatoblastoma cell through EPHB2 activation upon IGF1 stimulation. May play a role in the signal transduction in response to insulin and to a lesser extent in response to IL4 and GH on mitogenesis. Plays a role in growth, reproduction and glucose homeostasis. May act as negative regulators of the IGF1 signaling pathway by	chrX:107975898 c.3677G>C(E1)	p.1226,P>R	0.03	0

Table S3 - List of 489 non-synonymous and frame-shifting variants which were predicted to be damaging (X: termination codon).

Gene	Uniprot function	Genome position (hg19)	AA position & change	SIFT	Polyphen-2
	suppressing the function of IRS1 and IRS2.				
C1orf111	Uncharacterized protein C1orf111	chr1:162343909 c.715T>G(E3)	p.239,S>R	0.04	0.048
RGN	Regucalcin, Gluconolactonase with low activity towards other sugar lactones, including gulonolactone and galactonolactone. Can also hydrolyze diisopropyl phosphorofluoridate and phenylacetate (in vitro). Calcium-binding protein. Modulates Ca2+ signaling, and Ca2+-dependent cellular processes and enzyme activities (By similarity).	chrX:46943820 c.167C>T(E3)	p.56,A>V	0.19	0.244
UNC5A	Netrin receptor UNC5A, Receptor for netrin required for axon guidance. Functions in the netrin signaling pathway and promotes neurite outgrowth in response to NTN1. Mediates axon repulsion of neuronal growth cones in the developing nervous system in response to netrin. Axon repulsion in growth cones may be mediated by its association with DCC that may trigger signaling for repulsion. It also acts as a dependence receptor required for apoptosis induction when not associated with netrin ligand.	chr5:176305466 IVS12-10T>A splicing site			
KIAA146	Junctional protein associated with coronary artery disease,cell adhesion	chr10:30315856 c.3221A>G(E3)	p.1074,I>T	0.27	0.006
ODF2L	Outer dense fiber protein 2-like,	chr1:86822204 c.1441C>T(E14)	p.481,E>K	0.26	0.586
AGAP6	Arf-GAP with GTPase, ANK repeat and PH domain-containing protein 6, Putative GTPase-activating protein.	chr10:51748683-51748684 c.208(E1)c.209(E1)	p.70 R>W	0.2	0
AGAP5	Arf-GAP with GTPase, ANK repeat and PH domain-containing protein 5, Putative GTPase-activating protein.	chr10:75442543 c.406C>T(E5)	p.136,E>K	0.19	0.014
CMBL	Carboxymethylenebutenolidase homolog, Cysteine hydrolase. Can convert the prodrug olmesartan medoxomil into its pharmacologically active metabolite olmerstatan, an angiotensin receptor blocker, in liver and intestine. May also	chr5:10290894 IVS1-1C>T splicing site		_	_

Table S3 - List of 489 non-synonymous and frame-shifting variants which were predicted to be damaging (X: termination codon).

Gene	Uniprot function	Genome position (hg19)	AA position & change	SIFT	Polyphen-2
	activate beta-lactam antibiotics faropenem medoxomil and lenampicillin.				
CNOT4	CCR4-NOT transcription complex subunit 4, Has E3 ubiquitin ligase activity. Involved in activation of the JAK/STAT pathway.	chr7:135095397 c.689G>A(E7)	p.230,A>V	0.01	0.139
TNFSF15	Tumor necrosis factor ligand superfamily member 15, Receptor for TNFRSF25 and TNFRSF6B. Mediates activation of NF-kappa-B. Inhibits vascular endothelial growth and angiogenesis (in vitro). Promotes activation of caspases and apoptosis.	chr9:117568230 c.63G>C(E1)	p.21,H>Q	0.46	0.001
BTRC	F-box/WD repeat-containing protein 1A, Substrate recognition component of a SCF (SKP1-CUL1-F-box protein) E3 ubiquitin-protein ligase complex which mediates the ubiquitination and subsequent proteasomal degradation of target proteins. Recognizes and binds to phosphorylated target proteins. SCF(BTRC) mediates the ubiquitination of CTNNB1 and participates in Wnt signaling. SCF(BTRC) mediates the ubiquitination of NFKBIA, NFKBIB and NFKBIE; the degradation frees the associated NFKB1 to translocate into the nucleus and to activate transcription. Ubiquitination of NFKBIA occurs at 'Lys-21' and 'Lys-22'. SCF(BTRC) mediates the ubiquitination of CEP68; this is required for centriole separation during mitosis (PubMed:25704143, PubMed:25503564). SCF(BTRC) mediates the ubiquitination of phosphorylated NFKB1/nuclear factor NF-kappa-B p105 subunit, ATF4, CDC25A, DLG1, FBXO5, PER1, SMAD3, SMAD4, SNAI1 and probably NFKB2. Has an essential role in the control of the clock-dependent transcription via degradation of phosphorylated PER1 and PER2. May be involved in ubiquitination and subsequent proteasomal	chr10:103310574 c.1697C>A(E13)	p.566,P>H	0.02	0.139

Table S3 - List of 489 non-synonymous and frame-shifting variants which were predicted to be damaging (X: termination codon).

Gene	Uniprot function	Genome position (hg19)	AA position & change	SIFT	Polyphen-2
	degradation through a DBB1-CUL4 E3 ubiquitin-protein ligase. Required for activation of NFKB-mediated transcription by IL1B, MAP3K14, MAP3K1, IKBKB and TNF. Required for proteolytic processing of GLI3.				
STK36	Serine/threonine-protein kinase 36, Serine/threonine protein kinase which plays an important role in the sonic hedgehog (Shh) pathway by regulating the activity of GLI transcription factors. Controls the activity of the transcriptional regulators GLI1, GLI2 and GLI3 by opposing the effect of SUFU and promoting their nuclear localization. GLI2 requires an additional function of STK36 to become transcriptionally active, but the enzyme does not need to possess an active kinase catalytic site for this to occur. Required for postnatal development, possibly by regulating the homeostasis of cerebral spinal fluid or ciliary function (By similarity). Essential for construction of the central pair apparatus of motile cilia.	chr2:219549910 c.1339T>C(E11)	p.447,C>R	0.38	0.018
IL16	Pro-interleukin-16,Interleukin-16 stimulates a migratory response in CD4+ lymphocytes, monocytes, and eosinophils. Primes CD4+ T-cells for IL-2 and IL-15 responsiveness. Also induces T-lymphocyte expression of interleukin 2 receptor. Ligand for CD4. Isoform 1 may act as a scaffolding protein that anchors ion channels in the membrane. Isoform 3 is involved in cell cycle progression in T-cells. Appears to be involved in transcriptional regulation of SKP2 and is probably part of a transcriptional repression complex on the core promoter of the SKP2 gene. May act as a scaffold for GABPB1 (the DNA-binding subunit the GABP transcription factor complex) and HDAC3 thus	chr15:81595990 c.3419C>T(E16)	p.1140,T>M	0.01	1

Table S3 - List of 489 non-synonymous and frame-shifting variants which were predicted to be damaging (X: termination codon).

Gene	Uniprot function	Genome position (hg19)	AA position & change	SIFT	Polyphen-2
	maintaining transcriptional repression and blocking cell cycle progression in resting T-cells.				
MICB	MHC class I polypeptide-related sequence B, Seems to have no role in antigen presentation. Acts as a stress-induced self-antigen that is recognized by gamma delta T cells. Ligand for the KLRK1/NKG2D receptor. Binding to KLRK1 leads to cell lysis	chr6:31477681 c.1147A>G(E6)	p.383,T>A	0	0
		chr6:31473546 c.223A>G(E2)	p.75,N>D	0.44	0.003
SPATA2	Spermatogenesis-associated protein 20, May play a role in fertility regulation.	chr17:48629395 c.1763A>G(E13)	p.588,Y>C	0	0.997
ACSM4	Acyl-coenzyme A synthetase ACSM4, mitochondrial, Has medium-chain fatty acid:CoA ligase activity with broad substrate specificity (in vitro). Acts on acids from C4 to C(11) and on the corresponding 3-hydroxy- and 2,3- or 3,4-unsaturated acids (in vitro) (By similarity).	chr12:7476145 c.1297T>G(E9)	p.433,S>A	0.14	0.011
		chr12:7469737 c.625G>A(E4)	p.209,A>T	0.03	0.462
C13orf35	Putative protein ATP11AUN,	chr13:113333793 c.100A>C(E2)	p.34,K>Q	0	0.607
PHLDB3	Pleckstrin homology-like domain family B member 3, enzyme binding	chr19:44006318 c.331G>A(E3)	p.111,R>C	0	0.939
CXorf30	Cilia- and flagella-associated protein 47	chrX:36397596 c.1655T>G(E18)	p.552,M>R	0	0.999
ZNF460	Zinc finger protein 460,May be involved in transcriptional regulation.	chr19:57803539 c.1630G>A(E3)	p.544,A>T	0.26	0.093
POLDIP2	Polymerase delta-interacting protein 2, DNA binding	chr17:26684392-26684394 c.82(E1)-c.80(E1):TTT deleted CTTG inserted		_	_

Table S3 - List of 489 non-synonymous and frame-shifting variants which were predicted to be damaging (X: termination codon).

Gene	Uniprot function	Genome position (hg19)	AA position & change	SIFT	Polyphen-2
WWTR1	WW domain-containing transcription regulator protein 1, Transcriptional coactivator which acts as a downstream regulatory target in the Hippo signaling pathway that plays a pivotal role in organ size control and tumor suppression by restricting proliferation and promoting apoptosis. The core of this pathway is composed of a kinase cascade wherein STK3/MST2 and STK4/MST1, in complex with its regulatory protein SAV1, phosphorylates and activates LATS1/2 in complex with its regulatory protein MOB1, which in turn phosphorylates and inactivates YAP1 oncoprotein and WWTR1/TAZ. WWTR1 enhances PAX8 and NKX2-1/TTF1-dependent gene activation. Regulates the nuclear accumulation of SMADS and has a key role in coupling them to the transcriptional machinery such as the mediator complex. Regulates embryonic stem-cell self-renewal, promotes cell proliferation and epithelial-mesenchymal transition.	chr3:149238595-149238596 c.1200(E8)-c.119	p.400,L>Lfs2	_	_
DGKI	Diacylglycerol kinase iota, ATP binding, diacylglycerol kinase activity, GTPase inhibitor activity, metal ion binding	chr7:137294319 c.1030T>C(E9)	p.344,T>A	0.77	0.839
SPATA5	Spermatogenesis-associated protein 5-like protein 1,	chr15:45707953 c.1813A>G(E5)	p.605,R>G	0	0.209
ZNF669	Zinc finger protein 669, May be involved in transcriptional regulation.	chr1:247267248 c.254G>T(E1)	p.85,P>H	0.02	0.908
NBPF10	Neuroblastoma breakpoint family member 10, poly(A) RNA binding	chr1:145296478 c.400G>T(E3)	p.134,D>Y	0.01	0.635
OR2A12	Olfactory receptor 2A12, G-protein coupled receptor activity,sensory perception of smell	chr7:143792300 c.100T>C(E1)	p.34,Y>H	0	0.98
NBPF16	Neuroblastoma breakpoint family member 15	chr1:148754942 c.1598A>T(E14)	p.533,Q>L	0.05	0.872
		chr1:148754858 c.1514T>C(E14)	p.505,V>A	0.31	0.061
TTC12	Tetratricopeptide repeat protein 12	chr11:113210182 c.812A>T(E10)	p.271,E>V	0.01	0.905

Table S3 - List of 489 non-synonymous and frame-shifting variants which were predicted to be damaging (X: termination codon).

Gene	Uniprot function	Genome position (hg19)	AA position & change	SIFT	Polyphen-2
OR5B17	Olfactory receptor 5B17, G-protein coupled receptor activity,odorant binding,olfactory receptor activity	chr11:58125792 c.751T>C(E1)	p.251,I>V	0	0.008
PHLDA1	Pleckstrin homology-like domain family A member 1, Seems to be involved in regulation of apoptosis. May be involved in detachment-mediated programmed cell death. May mediate apoptosis during neuronal development. May be involved in regulation of anti-apoptotic effects of IGF1. May be involved in translational regulation.	chr12:76424938-76424940 c.584(E1)-c.582(E1	p.194-195, QQ>Q	_	_
ZBP1	Insulin-like growth factor 2 mRNA-binding protein 1, RNA-binding factor that recruits target transcripts to cytoplasmic protein-RNA complexes (mRNPs). This transcript 'caging' into mRNPs allows mRNA transport and transient storage. It also modulates the rate and location at which target transcripts encounter the translational apparatus and shields them from endonuclease attacks or microRNA-mediated degradation. Plays a direct role in the transport and translation of transcripts required for axonal regeneration in adult sensory neurons (By similarity). Regulates localized beta-actin/ACTB mRNA translation, a crucial process for cell polarity, cell migration and neurite outgrowth. Co-transcriptionally associates with the ACTB mRNA in the nucleus. This binding involves a conserved 54-nucleotide element in the ACTB mRNA 3'-UTR, known as the 'zipcode'. The RNP thus formed is exported to the cytoplasm, binds to a motor protein and is transported along the cytoskeleton to the cell periphery. During transport, prevents ACTB mRNA from being translated into protein. When the RNP complex reaches its destination near the plasma membrane, IGF2BP1 is phosphorylated. This releases the mRNA, allowing ribosomal 40S and 60S subunits	chr20:56190060 c.385C>T(E4)	p.129,A>T	0.03	0.998

Table S3 - List of 489 non-synonymous and frame-shifting variants which were predicted to be damaging (X: termination codon).

Gene	Uniprot function	Genome position (hg19)	AA position & change	SIFT	Polyphen-2
	to assemble and initiate ACTB protein synthesis. Monomeric ACTB then assembles into the subcortical actin cytoskeleton (By similarity). During neuronal development, key regulator of neurite outgrowth, growth cone guidance and neuronal cell migration, presumably through the spatiotemporal fine tuning of protein synthesis, such as that of ACTB (By similarity). May regulate mRNA transport to activated synapses (By similarity). Binds to and stabilizes ABCB1/MDR-1 mRNA (By similarity). During interstinal wound repair, interacts with and stabilizes PTGS2 transcript. PTGS2 mRNA stabilization may be crucial for colonic mucosal wound healing (By similarity). Binds to the 3'-UTR of IGF2 mRNA by a mechanism of cooperative and sequential dimerization and regulates IGF2 mRNA subcellular localization and translation. Binds to MYC mRNA, in the coding region instability determinant (CRD) of the open reading frame (ORF), hence prevents MYC cleavage by endonucleases and possibly microRNA targeting to MYC-CRD. Binds to the 3'-UTR of CD44 mRNA and stabilizes it, hence promotes cell adhesion and invadopodia formation in cancer cells. Binds to the oncofetal H19 transcript and to the neuron-specific TAU mRNA and regulates their localizations. Binds to and stabilizes BTRC/FBW1A mRNA. Binds to the adenine-rich autoregulatory sequence (ARS) located in PABPC1 mRNA and represses its translation. PABPC1 mRNA-binding is stimulated by PABPC1 protein. Prevents BTRC/FBW1A mRNA degradation by disrupting microRNA-dependent interaction with AGO2. Promotes the directed movement of tumor-derived cells by fine-tuning				

Table S3 - List of 489 non-synonymous and frame-shifting variants which were predicted to be damaging (X: termination codon).

Gene	Uniprot function	Genome position (hg19)	AA position & change	SIFT	Polyphen-2
	intracellular signaling networks. Binds to MAPK4 3'-UTR and inhibits its translation. Interacts with PTEN transcript open reading frame (ORF) and prevents mRNA decay. This combined action on MAPK4 (down-regulation) and PTEN (up-regulation) antagonizes HSPB1 phosphorylation, consequently it prevents G-actin sequestration by phosphorylated HSPB1, allowing F-actin polymerization. Hence enhances the velocity of cell migration and stimulates directed cell migration by PTEN-modulated polarization. Interacts with Hepatitis C virus (HCV) 5'-UTR and 3'-UTR and specifically enhances translation at the HCV IRES, but not 5'-cap-dependent translation, possibly by recruiting eIF3. Interacts with HIV-1 GAG protein and blocks the formation of infectious HIV-1 particles. Reduces HIV-1 assembly by inhibiting viral RNA packaging, as well as assembly and processing of GAG protein on cellular membranes. During cellular stress, such as oxidative stress or heat shock, stabilizes target mRNAs that are recruited to stress granules, including CD44, IGF2, MAPK4, MYC, PTEN, RAPGEF2 and RPS6KA5 transcripts.				
PHLDA3	Pleckstrin homology-like domain family A member 3,p53/TP53-regulated repressor of Akt/AKT1 signaling. Represses AKT1 by preventing AKT1-binding to membrane lipids, thereby inhibiting AKT1 translocation to the cellular membrane and activation. Contributes to p53/TP53-dependent apoptosis by repressing AKT1 activity. Its direct transcription regulation by p53/TP53 may explain how p53/TP53 can negatively regulate AKT1. May act as a tumor suppressor.	chr1:201437730 c.185T>C(E1)	p.62,E>G	0	0.702

Table S3 - List of 489 non-synonymous and frame-shifting variants which were predicted to be damaging (X: termination codon).

Gene	Uniprot function	Genome position (hg19)	AA position & change	SIFT	Polyphen-2
FBXW10	F-box/WD repeat-containing protein 10,Probable substrate-recognition component of a SCF (SKP1-CUL1-F-box protein)-type E3 ubiquitin ligase complex which mediates the ubiquitination and subsequent proteasomal degradation of target proteins. Overexpression is leading to degradation of CBX5 and CBX1.	chr17:18670067 c.1596A>C(E9)	p.532,R>S	0.02	0.041
SLC4A9	Anion exchange protein 4,Probable apical anion exchanger of the kidney cortex.	chr5:139742594 c.905G>A(E7)	p.302,R>Q	0.01	0.892
FBXW12	F-box/WD repeat-containing protein 12,Substrate-recognition component of the SCF (SKP1-CUL1-F-box protein)-type E3 ubiquitin ligase complex.	chr3:48419880 c.422T>C(E5)	p.141,L>P	0	0.997
DGKB	Diacylglycerol kinase beta, Exhibits high phosphorylation activity for long-chain diacylglycerols.	chr7:14188788 c.2383C>T(E25)	p.795,V>I	0.45	0.001
ZNF175	Zinc finger protein 175, Down-regulates the expression of several chemokine receptors. Interferes with HIV-1 replication by suppressing Tat-induced viral LTR promoter activity	chr19:52091581 c.1997C>T(E5)	p.666,T>M	0	0.798
CLEC10A	C-type lectin domain family 10 member A,Probable role in regulating adaptive and innate immune responses. Binds in a calcium-dependent manner to terminal galactose and N-acetylgalactosamine units, linked to serine or threonine. These sugar moieties are known as Tn-Ag and are expressed in a variety of carcinoma cells.	chr17:6981331 c.169C>T(E3)	p.57,V>M	0	0.997
HDGFRP	Hepatoma-derived growth factor-related protein 2, Involved in cellular growth control, through the regulation of cyclin D1 expression.	chr19:4491642 c.569C>T(E5)	p.190,S>L	0.02	0.519
ABHD1	Protein ABHD1, carboxylic ester hydrolase activity	chr2:27353431 c.1037C>A(E9)	p.346,P>H	0	0.975
PCDHB1	Protocadherin beta-12, Potential calcium-dependent cell-adhesion protein. May be involved in the establishment and maintenance of specific	chr5:140590723 c.2244C>A(E1)	p.748,S>R	0.03	0.078

Table S3 - List of 489 non-synonymous and frame-shifting variants which were predicted to be damaging (X: termination codon).

Gene	Uniprot function	Genome position (hg19)	AA position & change	SIFT	Polyphen-2
	neuronal connections in the brain.				
KIAA173	Centrosomal protein of 295 kDa,Centriole-enriched protein that mediates centriole-to-centrosome conversion at late mitosis, but is dispensable for cartwheel removal or centriole disengagement.	chr11:93440001 c.5687C>T(E18)	p.1896,P>L	0.12	0.147
POC1B-G	Polypeptide N-acetylgalactosaminyltransferase, involved in the pathway protein glycosylation, which is part of Protein modification.	chr12:89916672 c.1646C>T(E3)	p.549,R>Q	0.16	0.763
GANAB	Neutral alpha-glucosidase AB, Cleaves sequentially the 2 innermost alpha-1,3-linked glucose residues from the Glc2Man9GlcNAc2 oligosaccharide precursor of immature glycoproteins.	chr11:62398640 c.1012T>C(E10)	p.338,M>V	0.28	0.014
PHYHD1	Phytanoyl-CoA dioxygenase domain-containing protein 1, Isoform 1 has alpha-ketoglutarate-dependent dioxygenase activity. Does not show detectable activity towards fatty acid CoA thioesters. Is not expected to be active with phytanoyl CoA. Isoform 2 and isoform 3 probably lack enzyme activity.	chr9:131684610 c.8G>C(E3)	p.3,C>S	0.14	0.005
TTC40	Cilia- and flagella-associated protein 46, As part of the central apparatus of the cilium axoneme plays a role in cilium movement.	chr10:134752216 c.413T>C(E5)	p.138,Q>R	0.1	0.002
WDR46	WD repeat-containing protein 46, Scaffold component of the nucleolar structure. Required for localization of DDX21 and NCL to the granular compartment of the nucleolus.	chr6:33255406 c.551G>A(E7)	p.184,A>V	0.01	0.322
WNT9B	Protein Wnt-9b, Ligand for members of the frizzled family of seven transmembrane receptors. Probable developmental protein. May be a signaling molecule which affects the development of discrete regions of tissues. Is likely to signal over only few cell diameters (By similarity).	chr17:44953783 c.773G>A(E4)	p.258,R>H	0.25	0.002

Table S3 - List of 489 non-synonymous and frame-shifting variants which were predicted to be damaging (X: termination codon).

Gene	Uniprot function	Genome position (hg19)	AA position & change	SIFT	Polyphen-2
AS3MT	Arsenite methyltransferase, Catalyzes the transfer of a methyl group from AdoMet to trivalent arsenicals producing methylated and dimethylated arsenicals. It methylates arsenite to form methylarsonate, Me-AsO3H2, which is reduced by methylarsonate reductase to methylarsonite, Me-As(OH)2. Methylarsonite is also a substrate and it is converted into the much less toxic compound dimethylarsinate (cacodylate), Me2As(O)-OH (By similarity).	chr10:104632301 c.267C>A(E4)	p.89,S>R	0	0.997
ZNF135	Zinc finger protein 135, Plays a role in the regulation of cell morphology and cytoskeletal organization. May be involved in transcriptional regulation.	chr19:58574832 c.215C>T(E3)	p.72,P>L	0.1	0.005
CLIC3	Chloride intracellular channel protein 3, Can insert into membranes and form chloride ion channels. May participate in cellular growth control.	chr9:139889141 c.703G>A(E6)	p.235,P>S	0.01	0.554
KSR2	Kinase suppressor of Ras 2, Location-regulated scaffold connecting MEK to RAF. Has very low protein kinase activity and can phosphorylate MAP2K1 at several Ser and Thr residues with very low efficiency (in vitro). Interaction with BRAF enhances KSR2-mediated phosphorylation of MAP2K1 (in vitro). Blocks MAP3K8 kinase activity and MAP3K8-mediated signaling. Acts as a negative regulator of MAP3K3-mediated activation of ERK, JNK and NF-kappa-B pathways, inhibiting MAP3K3-mediated interleukin-8 production.	chr12:117969507 c.1606G>A(E11)	p.536,H>Y	0.77	0.005
IGFN1	Immunoglobulin-like and fibronectin type III domain-containing protein 1,	chr1:201186537 c.9718C>A(E17)	p.3240,R>S	_	0.673
		chr1:201194003 IVS21+1G>T splicing		_	_
MTMR12	Myotubularin-related protein 12, Catalytically inactive phosphatase that plays a role as an adapter for the phosphatase myotubularin to regulate myotubularin intracellular location.	chr5:32263226 c.706A>T(E7)	p.236,C>S	0.01	0.246

Table S3 - List of 489 non-synonymous and frame-shifting variants which were predicted to be damaging (X: termination codon).

Gene	Uniprot function	Genome position (hg19)	AA position & change	SIFT	Polyphen-2
PJA2	E3 ubiquitin-protein ligase Praja-2, Has E2-dependent E3 ubiquitin-protein ligase activity. Responsible for ubiquitination of cAMP-dependent protein kinase type I and type II-alpha/beta regulatory subunits and for targeting them for proteasomal degradation. Essential for PKA-mediated long-term memory processes.	chr5:108714626 c.562C>T(E4)	p.188,V>M	0.3	0.077
KIAA058	Protein TALPID3, Required for ciliogenesis and sonic hedgehog/SHH signaling. Required for the centrosomal recruitment of RAB8A and for the targeting of centriole satellite proteins to centrosomes such as of PCM1. May play a role in early ciliogenesis in the disappearance of centriolar satellites that preceeds ciliary vesicle formation (PubMed:24421332). Involved in regulation of cell intracellular organization. Involved in regulation of cell polarity (By similarity). Required for asymmetrical localization of CEP120 to daughter centrioles (By similarity).	chr14:58920178 c.1399G>A(E11)	p.467,E>K	0.03	0.562
EXOSC2	Exosome complex component RRP4, Non-catalytic component of the RNA exosome complex which has 3'->5' exoribonuclease activity and participates in a multitude of cellular RNA processing and degradation events. In the nucleus, the RNA exosome complex is involved in proper maturation of stable RNA species such as rRNA, snRNA and snoRNA, in the elimination of RNA processing by-products and non-coding 'pervasive' transcripts, such as antisense RNA species and promoter-upstream transcripts (PROMPTs), and of mRNAs with processing defects, thereby limiting or excluding their export to the cytoplasm. The RNA exosome may be involved in Ig class switch recombination (CSR) and/or Ig variable region somatic hypermutation (SHM) by targeting AICDA deamination activity to transcribed	chr9:133569197 c.19C>T(E1)	p.7,L>F		

Table S3 - List of 489 non-synonymous and frame-shifting variants which were predicted to be damaging (X: termination codon).

Gene	Uniprot function	Genome position (hg19)	AA position & change	SIFT	Polyphen-2
	dsDNA substrates. In the cytoplasm, the RNA exosome complex is involved in general mRNA turnover and specifically degrades inherently unstable mRNAs containing AU-rich elements (AREs) within their 3' untranslated regions, and in RNA surveillance pathways, preventing translation of aberrant mRNAs. It seems to be involved in degradation of histone mRNA. The catalytic inactive RNA exosome core complex of 9 subunits (Exo-9) is proposed to play a pivotal role in the binding and presentation of RNA for ribonucleolysis, and to serve as a scaffold for the association with catalytic subunits and accessory proteins or complexes. EXOSC2 as peripheral part of the Exo-9 complex stabilizes the hexameric ring of RNase PH-domain subunits through contacts with EXOSC4 and EXOSC7.				
ANP32E	Acidic leucine-rich nuclear phosphoprotein 32 family member E, Histone chaperone that specifically mediates the genome-wide removal of histone H2A.Z/H2AFZ from the nucleosome: removes H2A.Z/H2AFZ from its normal sites of deposition, especially from enhancer and insulator regions. Not involved in deposition of H2A.Z/H2AFZ in the nucleosome. May stabilize the evicted H2A.Z/H2AFZ-H2B dimer, thus shifting the equilibrium towards dissociation and the off-chromatin state (PubMed:24463511). Inhibits activity of protein phosphatase 2A (PP2A). Does not inhibit protein phosphatase 1. May play a role in cerebellar development and synaptogenesis.	chr1:150199040-150199045 c.458(E4)-c.453(E4):TCCTCT deleted		–	–
EGFL7	Epidermal growth factor-like protein 7, Regulates vascular tubulogenesis in vivo. Inhibits platelet-derived growth factor (PDGF)-BB-induced smooth muscle cell migration and promotes	chr9:139564080 c.220C>T(E6)	p.74,R>C	0.02	1

Table S3 - List of 489 non-synonymous and frame-shifting variants which were predicted to be damaging (X: termination codon).

Gene	Uniprot function	Genome position (hg19)	AA position & change	SIFT	Polyphen-2
	endothelial cell adhesion to the extracellular matrix and angiogenesis.				
NFKBIZ	NF-kappa-B inhibitor zeta, Involved in regulation of NF-kappa-B transcription factor complexes. Inhibits NF-kappa-B activity without affecting its nuclear translocation upon stimulation. Inhibits DNA-binding of RELA and NFKB1/p50, and of the NF-kappa-B p65-p50 heterodimer and the NF-kappa-B p50-p50 homodimer. Seems also to activate NF-kappa-B-mediated transcription. In vitro, upon association with NFKB1/p50 has transcriptional activation activity and, together with NFKB1/p50 and RELA, is recruited to LCN2 promoters. Promotes transcription of LCN2 and DEFB4. Is recruited to IL-6 promoters and activates IL-6 but decreases TNF-alpha production in response to LPS. Seems to be involved in the induction of inflammatory genes activated through TLR/IL-1 receptor signaling. May promote apoptosis (By similarity).	chr3:101572365 c.695C>T(E6)	p.232,S>L	0.46	0.001
CDC7	Cell division cycle 7-related protein kinase, Seems to phosphorylate critical substrates that regulate the G1/S phase transition and/or DNA replication. Can phosphorylates MCM2 and MCM3.	chr1:91985828 c.1322A>G(E11)	p.441,K>R	0.22	0.4
TMEM10	Transmembrane protein 104,	chr17:72832650 c.1315G>A(E10)	p.439,A>T	0.18	0.937
FAM154	Stabilizer of axonemal microtubules 1, May play a role in the regulation of cilium length. Stabilizes microtubules at low temperature.	chr9:18928504 c.971G>C(E4)	p.324,P>R	0	0.97
ZNF496	Zinc finger protein 496, DNA-binding transcription factor that can both act as an activator and a repressor.	chr1:247492078 c.481C>T(E4)	p.161,D>N	0.16	0.002
ZNF737	Zinc finger protein 737, May be involved in transcriptional regulation.	chr19:20727688 c.1321T>C(E4)	p.441,T>A	0.05	0.006
TMEM10	Transmembrane protein 108	chr3:133098949 c.394C>A(E4)	p.132,R>S	0.05	0.013
		chr3:133098661 c.106G>C(E4)	p.36,E>Q	0.01	0.176

Table S3 - List of 489 non-synonymous and frame-shifting variants which were predicted to be damaging (X: termination codon).

Gene	Uniprot function	Genome position (hg19)	AA position & change	SIFT	Polyphen-2
C22orf31	Uncharacterized protein C22orf31	chr22:29456776 c.59G>A(E2)	p.20,S>F	0	0.911
PLEKHG	Pleckstrin homology domain-containing family G member 4B, Rho guanyl-nucleotide exchange factor activity	chr5:140474 c.52G>A(E1)	p.18,G>R	0.37	0.001
MMP27	Matrix metalloproteinase-27, Matrix metalloproteinases degrade protein components of the extracellular matrix such as fibronectin, laminin, gelatins and/or collagens.	chr11:102565731 c.1000C>T(E7)	p.334,E>K	0	1
SPI1	Transcription factor PU.1, Binds to the PU-box, a purine-rich DNA sequence (5'-GAGGAA-3') that can act as a lymphoid-specific enhancer. This protein is a transcriptional activator that may be specifically involved in the differentiation or activation of macrophages or B-cells. Also binds RNA and may modulate pre-mRNA splicing (By similarity).	chr11:47380422 c.469C>T(E4)	p.157,G>R	0.44	0.024
ZNFX1	NFX1-type zinc finger-containing protein 1, poly(A) RNA binding, transcription factor activity, sequence-specific DNA binding, zinc ion binding	chr20:47886611 c.1738G>T(E3)	p.580,P>T	0.15	0.001
SYTL5	Synaptotagmin-like protein 5,May act as Rab effector protein and play a role in vesicle trafficking. Binds phospholipids.	chrX:37981474 IVS15+6T>A splicing site		_	_
MAP2	Microtubule-associated protein 2, The exact function of MAP2 is unknown but MAPs may stabilize the microtubules against depolymerization. They also seem to have a stiffening effect on microtubules.	chr2:210557706 c.812C>T(E7)	p.271,T>M	0.2	0.007
PKP3	Plakophilin-3, May play a role in junctional plaques.	chr11:400536 c.1568G>A(E8)	p.523,S>N	0.24	0.711
SRSF6	Serine/arginine-rich splicing factor 6, Plays a role in constitutive splicing and modulates the selection of alternative splice sites. Plays a role in the alternative splicing of MAPT/Tau exon 10. Binds to alternative exons of TNC pre-mRNA and promotes the expression of alternatively spliced	chr20:42089486 c.818G>A(E6)	p.273,R>Q	0.15	0

Table S3 - List of 489 non-synonymous and frame-shifting variants which were predicted to be damaging (X: termination codon).

Gene	Uniprot function	Genome position (hg19)	AA position & change	SIFT	Polyphen-2
	TNC. Plays a role in wound healing and in the regulation of keratinocyte differentiation and proliferation via its role in alternative splicing.				
FNBP1	Formin-binding protein 1, May act as a link between RND2 signaling and regulation of the actin cytoskeleton (By similarity). Required to coordinate membrane tubulation with reorganization of the actin cytoskeleton during the late stage of clathrin-mediated endocytosis. Binds to lipids such as phosphatidylinositol 4,5-bisphosphate and phosphatidylserine and promotes membrane invagination and the formation of tubules. Also enhances actin polymerization via the recruitment of WASL/N-WASP, which in turn activates the Arp2/3 complex. Actin polymerization may promote the fission of membrane tubules to form endocytic vesicles. May be required for the lysosomal retention of FASLG/FASL.	chr9:132662784 c.1471C>T(E14)	p.491,E>K	0.03	0.001
WDR66	WD repeat-containing protein 66,	chr12:122359397-122359398 c.186(E2)-c.187(	p.62-63 -/RRRRRX	_	_
KIAA202	Uncharacterized protein KIAA2026	chr9:5920883 c.5113C>T(E8)	p.1705,G>R	0	0.68
		chr9:5920649 c.5347G>A(E8)	p.1783,R>W	0.05	0.002
IFI27L2	Interferon alpha-inducible protein 27-like protein 2,	chr14:94594327 c.202C>A(E4)	p.68,A>S	0.04	0.918
COL16A	Collagen alpha-1(XVI) chain, Involved in mediating cell attachment and inducing integrin-mediated cellular reactions, such as cell spreading and alterations in cell morphology.	chr1:32119605 c.4397A>C(E69)	p.1466,M>R	_	0.758
EMILIN2	May be responsible for anchoring smooth muscle cells to elastic fibers, and may be involved not only in the formation of the elastic fiber, but also in the processes that regulate vessel assembly. Has cell adhesive capacity.	chr18:2890715 c.590C>T(E4)	p.197,T>M	0.09	0.116

Table S3 - List of 489 non-synonymous and frame-shifting variants which were predicted to be damaging (X: termination codon).

Gene	Uniprot function	Genome position (hg19)	AA position & change	SIFT	Polyphen-2
DUSP22	Dual specificity protein phosphatase 22, Activates the Jnk signaling pathway. Dephosphorylates and deactivates p38 and stress-activated protein kinase/c-Jun N-terminal kinase (SAPK/JNK) (By similarity).	chr6:345904 c.239G>A(E5)	p.80,R>H	0.39	0.159
PCDH12	Protocadherin-12,Cellular adhesion molecule that may play an important role in cell-cell interactions at interendothelial junctions. Promotes homotypic calcium-dependent aggregation and adhesion and clusters at intercellular junctions. Unable to bind to catenins, weakly associates with the cytoskeleton (By similarity).	chr5:141324988 c.3513C>G(E4)	p.1171,E>D	0.11	0
MYADM	Myeloid-associated differentiation marker-like protein 2	chr17:79899044 c.574C>T(E3)	p.192,V>M	0.11	0.023
CCDC80	Coiled-coil domain-containing protein 80, Promotes cell adhesion and matrix assembly.	chr3:112358455 c.298C>T(E2)	p.100,A>T	0.43	0.029
FOXRED	FAD-dependent oxidoreductase domain-containing protein 2, Probable flavoprotein which may function in endoplasmic reticulum associated degradation (ERAD). May bind non-native proteins in the endoplasmic reticulum and target them to the ubiquitination machinery for subsequent degradation.	chr22:36892103 c.1535A>C(E7)	p.512,F>C	0.16	0.571
MKI67	Antigen KI-67, Thought to be required for maintaining cell proliferation.	chr10:129904619 c.4405G>C(E12)	p.1469,L>V	0.08	0.94
RECQL5	ATP-dependent DNA helicase Q5, Isoform beta is a DNA helicase that plays an important role in DNA replication, transcription and repair. Inhibits elongation of stalled transcripts at DNA damage sites by binding to the RNA polymerase II subunit POLR2A and blocking the TCEA1 binding site. Required for mitotic chromosome separation after cross-over events and cell cycle progress. Required for efficient DNA repair, including repair of inter-strand cross-links. Stimulates DNA decatenation mediated by TOP2A. Prevents sister	chr17:73662633 c.5C>T(E2)	p.2,S>N	0.05	0.014

Table S3 - List of 489 non-synonymous and frame-shifting variants which were predicted to be damaging (X: termination codon).

Gene	Uniprot function	Genome position (hg19)	AA position & change	SIFT	Polyphen-2
	chromatid exchange and homologous recombination.				
CDC42EP	Cdc42 effector protein 5, Probably involved in the organization of the actin cytoskeleton. May act downstream of CDC42 to induce actin filament assembly leading to cell shape changes. Induces pseudopodia formation in fibroblasts. Inhibits MAPK8 independently of CDC42 binding. Controls septin organization and this effect is negatively regulated by CDC42 (By similarity).	chr19:54976403 c.329G>A(E3)	p.110,A>V	0.28	0.015
PRPF40A	Pre-mRNA-processing factor 40 homolog A, Binds to WASL/N-WASP and suppresses its translocation from the nucleus to the cytoplasm, thereby inhibiting its cytoplasmic function (By similarity). Plays a role in the regulation of cell morphology and cytoskeletal organization. Required in the control of cell shape and migration. May play a role in cytokinesis. May be involved in pre-mRNA splicing.	chr2:153549562 c.318A>T(E4)	p.106,D>E	0.4	0.417
CASZ1	Zinc finger protein castor homolog 1, Transcription factor involved in vascular assembly and morphogenesis through direct transcriptional regulation of EGFL7.	chr1:10725587 c.58C>T(E5)	p.20,A>T	0.36	0.199
RPTN	Repetin, Involved in the cornified cell envelope formation. Multifunctional epidermal matrix protein. Reversibly binds calcium.	chr1:152128610 c.965G>A(E3)	p.322,T>M	0.06	0.14
WDR52	Cilia- and flagella-associated protein 44	chr3:113015612 c.5198C>T(E33)	p.1733,R>Q	0.3	0.005
TRIML1	Probable E3 ubiquitin-protein ligase TRIML1, Probable E3 ubiquitin-protein ligase which plays an important role in blastocyst development.	chr4:189060983 c.271G>C(E1)	p.91,E>Q	0.42	0.011

Table S3 - List of 489 non-synonymous and frame-shifting variants which were predicted to be damaging (X: termination codon).

Gene	Uniprot function	Genome position (hg19)	AA position & change	SIFT	Polyphen-2
ARHGEF	Rho guanine nucleotide exchange factor 28, Functions as a RHOA-specific guanine nucleotide exchange factor regulating signaling pathways downstream of integrins and growth factor receptors. Functions in axonal branching, synapse formation and dendritic morphogenesis. Functions also in focal adhesion formation, cell motility and B-lymphocytes activation. May regulate NEFL expression and aggregation and play a role in apoptosis (By similarity).	chr5:73136566 c.1408A>G(E11)	p.470,S>G	0.43	0.007
DNAH14	Dynein heavy chain 14, axonemal, Force generating protein of respiratory cilia. Produces force towards the minus ends of microtubules. Dynein has ATPase activity; the force-producing power stroke is thought to occur on release of ADP. Involved in sperm motility; implicated in sperm flagellar assembly (By similarity).	chr1:225328430 IVS26-3T>G splicing site	_	_	_
IGFBPL1	Insulin-like growth factor-binding protein-like 1, IGF-binding proteins prolong the half-life of IGFs and have been shown to either inhibit or stimulate the growth promoting effects of the IGFs in cell culture. They alter the interaction of IGFs with their cell surface receptors (By similarity). May be a putative tumor suppressor protein.	chr9:38414152 c.509G>A(E2)	p.170,A>V	0	0.569
DNAH12	Dynein heavy chain 12, axonemal, Force generating protein of respiratory cilia. Produces force towards the minus ends of microtubules. Dynein has ATPase activity; the force-producing power stroke is thought to occur on release of ADP. Involved in sperm motility; implicated in sperm flagellar assembly (By similarity).	chr3:57494251 c.559T>C(E7)	p.187,N>D	0.26	0.133
KIF4B	Chromosome-associated kinesin KIF4B, Motor protein that translocates PRC1 to the plus ends of interdigitating spindle microtubules during the metaphase to anaphase transition, an essential step	chr5:154395689 c.2270C>T(E1)	p.757,T>I	0.14	0.049

Table S3 - List of 489 non-synonymous and frame-shifting variants which were predicted to be damaging (X: termination codon).

Gene	Uniprot function	Genome position (hg19)	AA position & change	SIFT	Polyphen-2
	for the formation of an organized central spindle midzone and midbody and for successful cytokinesis. May play a role in mitotic chromosomal positioning and bipolar spindle stabilization (By similarity).	chr5:154394077 c.658A>G(E1)	p.220,I>V	0.27	0.007
		chr5:154394985 c.1566G>A(E1)	p.522,M>I	0.02	0.354
CRTC2	CREB-regulated transcription coactivator 2, Transcriptional coactivator for CREB1 which activates transcription through both consensus and variant cAMP response element (CRE) sites. Acts as a coactivator, in the SIK/TORC signaling pathway, being active when dephosphorylated and acts independently of CREB1 'Ser-133' phosphorylation. Enhances the interaction of CREB1 with TAF4. Regulates gluconeogenesis as a component of the LKB1/AMPK/TORC2 signaling pathway. Regulates the expression of specific genes such as the steroidogenic gene, StAR. Potent coactivator of PPARGC1A and inducer of mitochondrial biogenesis in muscle cells. Also coactivator for TAX activation of the human T-cell leukemia virus type 1 (HTLV-1) long terminal repeats (LTR).	chr1:153921685 c.1580C>A(E12)	p.527,R>M	0.22	0.916
ADAMT	A disintegrin and metalloproteinase with thrombospondin motifs 1,Cleaves aggrecan, a cartilage proteoglycan, and may be involved in its turnover (By similarity). Has angiogenic inhibitor activity. Active metalloprotease, which may be associated with various inflammatory processes as well as development of cancer cachexia. May play a critical role in follicular rupture.	chr21:28210810 c.2152C>T(E8)	p.718,G>R	0.02	1

Table S3 - List of 489 non-synonymous and frame-shifting variants which were predicted to be damaging (X: termination codon).

Gene	Uniprot function	Genome position (hg19)	AA position & change	SIFT	Polyphen-2
SPAG5	Sperm-associated antigen 5, Essential component of the mitotic spindle required for normal chromosome segregation and progression into anaphase. Required for chromosome alignment, normal timing of sister chromatid segregation, and maintenance of spindle pole architecture (PubMed:17664331). In complex with SKAP, promotes stable microtubule-kinetochore attachments. May contribute to the regulation of separase activity. May regulate AURKA localization to mitotic spindle, but not to centrosomes and CCNB1 localization to both mitotic spindle and centrosomes. Involved in centriole duplication. Required for CDK5RAP2, CEP152, WDR62 and CEP63 centrosomal localization and promotes the centrosomal localization of CDK2. In non-mitotic cells, upon stress induction, inhibits mammalian target of rapamycin complex 1 (mTORC1) association and recruits the mTORC1 component RPTOR to stress granules (SGs), thereby preventing mTORC1 hyperactivation-induced apoptosis. May enhance GSK3B-mediated phosphorylation of other substrates, such as MAPT/TAU	chr17:26918889 c.1264G>A(E4)	p.422,R>W	0.19	0.024
TMEM13	Transmembrane protein 132C,	chr12:129190266 c.2753G>A(E9)	p.918,R>Q	0.03	0.187
ZNF500	Zinc finger protein 500, May be involved in transcriptional regulation.	chr16:4802874 c.946G>A(E6)	p.316,R>W	0.02	0.011
BTBD9	BTB/POZ domain-containing protein 9, ubiquitin protein ligase binding	chr6:38545467 c.1063T>C(E6)	p.355,M>V	0.42	0
KRTAP1	Keratin-associated protein 10-6, In the hair cortex, hair keratin intermediate filaments are embedded in an interfilamentous matrix, consisting of hair keratin-associated proteins (KRTAP), which are essential for the formation of a rigid and resistant hair shaft through their extensive disulfide bond cross-linking with abundant cysteine residues of	chr21:46011688 c.678C>G(E1)	p.226,Q>H	0.07	0

Table S3 - List of 489 non-synonymous and frame-shifting variants which were predicted to be damaging (X: termination codon).

Gene	Uniprot function	Genome position (hg19)	AA position & change	SIFT	Polyphen-2
	hair keratins. The matrix proteins include the high-sulfur and high-glycine-tyrosine keratins.				
MIIP	Migration and invasion-inhibitory protein, Inhibits glioma cells invasion and down-regulates adhesion- and motility-associated genes such as NFKB2 and ICAM1. Exhibits opposing effects to IGFBP2 on cell invasion.	chr1:12089845 c.739C>T(E7)	p.247,R>W	0	0.999
PTPRT	Receptor-type tyrosine-protein phosphatase T, May be involved in both signal transduction and cellular adhesion in the CNS.	chr20:41408876 c.550C>A(E4)	p.184,V>F	0	0.99
SIPA1L3	Signal-induced proliferation-associated 1-like protein 3,Plays a critical role in epithelial cell morphogenesis, polarity, adhesion and cytoskeletal organization in the lens	chr19:38572777 c.572G>A(E3)	p.191,R>Q	0.23	0.102
ZNF790	Zinc finger protein 790, May be involved in transcriptional regulation.	chr19:37310149 c.1097T>C(E5)	p.366,H>R	0	0.305
OLFML2	Olfactomedin-like protein 2B,	chr1:161970098 c.754C>T(E5)	p.252,V>M	0.08	0.571
ADAMT	A disintegrin and metalloproteinase with thrombospondin motifs 20, May play a role in tissue-remodeling process occurring in both normal and pathological conditions. May have a protease-independent function in the transport from the endoplasmic reticulum to the Golgi apparatus of secretory cargos, mediated by the GON domain.	chr12:43821184 c.4034G>A(E27)	p.1345,A>V	0.31	0.301
MYH1	Myosin-1, Muscle contraction.	chr17:10419776 c.184C>T(E3)	p.62,A>T	0.02	0.115
CLCA1	Calcium-activated chloride channel regulator 1,May be involved in mediating calcium-activated chloride conductance. May play critical roles in goblet cell metaplasia, mucus hypersecretion, cystic fibrosis and AHR. May be involved in the regulation of mucus production and/or secretion by goblet cells. Involved in the regulation of tissue inflammation in the innate immune response. May play a role as a tumor suppressor. Induces	chr1:86965595 c.2612C>T(E14)	p.871,P>L	0.07	0.011

Table S3 - List of 489 non-synonymous and frame-shifting variants which were predicted to be damaging (X: termination codon).

Gene	Uniprot function	Genome position (hg19)	AA position & change	SIFT	Polyphen-2
	MUC5AC.				
GBP7	Guanylate-binding protein 7, Hydrolyzes GTP to GMP in two consecutive cleavage reactions. Promote oxidative killing and deliver antimicrobial peptides to autophagolysosomes, providing broad host protection against different pathogen classes (By similarity).	chr1:89616096 c.788A>T(E6) p.263,M>K		0.08	0
ZNF107	Zinc finger protein 107, May be involved in transcriptional regulation.	chr7:64168205 c.1523C>A(E5)	p.508,S>Y	0.39	0.831
YEATS2	YEATS domain-containing protein 2, Component of the ATAC complex, a complex with histone acetyltransferase activity on histones H3 and H4.	chr3:183525827 c.4021A>G(E29)	p.1341,T>A	0.26	0.003
C20orf19	Uncharacterized protein C20orf194, May act as an effector for ARL3.	chr20:3278689 c.1916G>A(E22)	p.639,A>V	0.24	0.304
		chr20:3278765 c.1840G>A(E22)	p.614,H>Y	0.2	0.007
CUL9	Cullin-9, Core component of a Cul9-RING ubiquitin-protein ligase complex, a complex that mediates ubiquitination and subsequent degradation of BIRC5 and is required to maintain microtubule dynamics and genome integrity. Acts downstream of the 3M complex, which inhibits CUL9 activity, leading to prevent ubiquitination of BIRC5. Cytoplasmic anchor protein in p53/TP53-associated protein complex. Regulates the subcellular localization of p53/TP53 and subsequent function	chr6:43166412-43166414 c.2869(E12)-c.2871(	p.957 G>-	_	_
ADTRP	Androgen-dependent TFPI-regulating protein, Regulates the expression and the cell-associated anticoagulant activity of the inhibitor TFPI in endothelial cells (in vitro).	chr6:11766527 c.424C>T(E4)	p.142,V>M	0.2	0.038
ZNF778	Zinc finger protein 778, May be involved in transcriptional regulation.	chr16:89294081 c.1385C>T(E7)	p.462,S>L	0.03	0.928
ZNF251	Zinc finger protein 251, May be involved in transcriptional regulation.	chr8:145948467 c.578T>C(E5)	p.193,D>G	0.31	0
ANKLE1	Ankyrin repeat and LEM domain-containing protein 1,	chr19:17393770 c.419G>C(E4)	p.140,R>P	0.08	0.008

Table S3 - List of 489 non-synonymous and frame-shifting variants which were predicted to be damaging (X: termination codon).

Gene	Uniprot function	Genome position (hg19)	AA position & change	SIFT	Polyphen-2
RPL22L1	60S ribosomal protein L22-like 1, structural constituent of ribosome	chr3:170584249 c.289C>A(E4)	p.97,V>F	0	0.443
ZNF638	Zinc finger protein 638, Early regulator of adipogenesis that works as a transcription cofactor of CEBPs, controlling the expression of PPARG and probably of other proadipogenic genes, such as SREBF1 (By similarity). Binds to cytidine clusters in double-stranded DNA. May also regulate alternative splicing of target genes during adipogenesis (By similarity).	chr2:71592714 c.1873G>A(E6)	p.625,A>T	0.14	0.001
C14orf37	Uncharacterized protein C14orf37	chr14:58605991 c.86G>A(E2)	p.29,P>L	0	0.851
LRRD1	Leucine-rich repeat and death domain-containing protein 1, signal transduction	chr7:91780003 c.2123T>G(E3)	p.708,N>T	0.08	0.935
VPS54	Vacuolar protein sorting-associated protein 54, Acts as component of the GARP complex that is involved in retrograde transport from early and late endosomes to the trans-Golgi network (TGN). The GARP complex is required for the maintenance of the cycling of mannose 6-phosphate receptors between the TGN and endosomes, this cycling is necessary for proper lysosomal sorting of acid hydrolases such as CTSD. Within the GARP complex, required to tether the complex to the TGN. Not involved in endocytic recycling	chr2:64199317 c.404G>A(E4)	p.135,T>I	0.18	0.009
AURKAI	Aurora kinase A-interacting protein, May act as a negative regulator of Aurora-A kinase, by down-regulation through proteasome-dependent degradation.	chr1:1309567 c.311G>A(E3)	p.104,P>L	0.05	0.039
