# Supplementary figures and images for: Novel compound heterozygous mutations of ALDH1A3 contribute to anophthalmia in a non-consanguineous Chinese family
Source: Genet Mol Biol. 2017 Jun 5;40(2):430–5. doi: 10.1590/1678-4685-GMB-2016-0120 (PMC5488456; doi:10.1590/1678-4685-GMB-2016-0120)

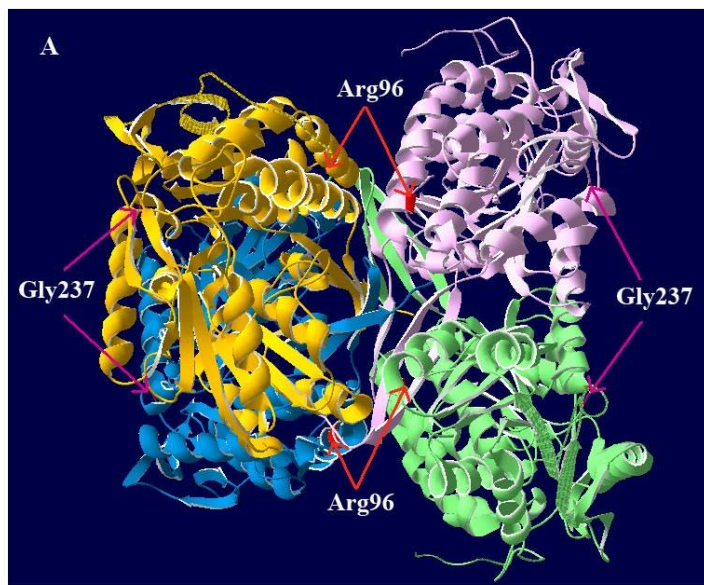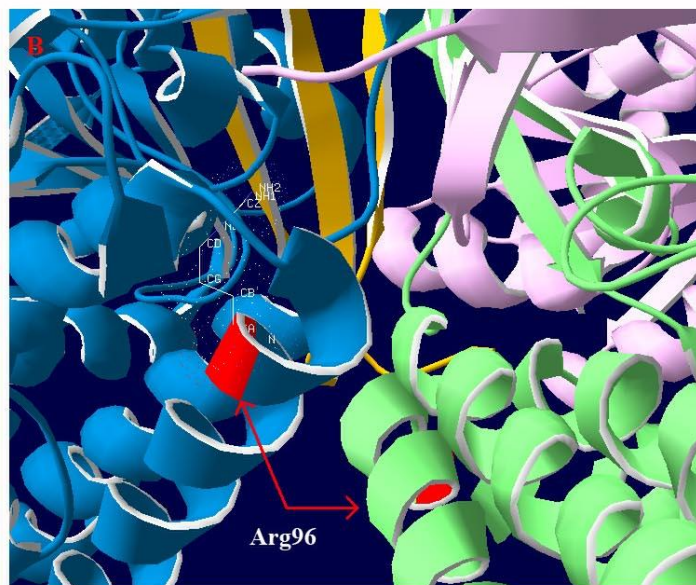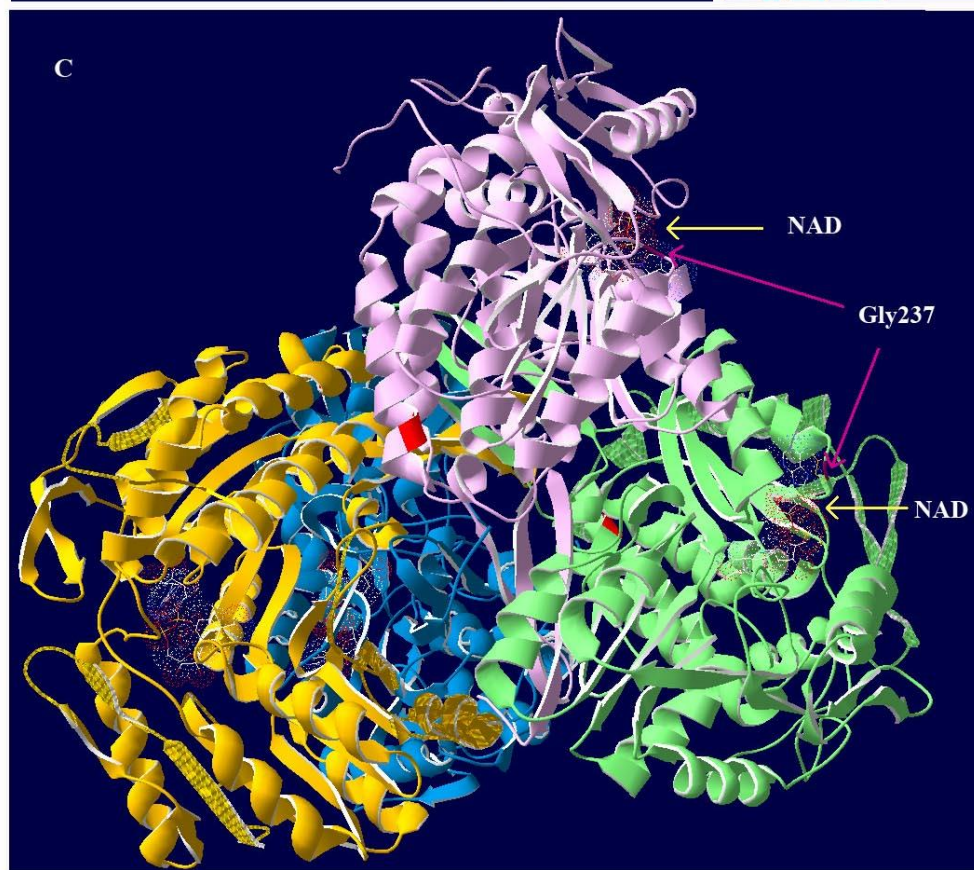

Supplement: Supplementary file 4 [file 1415-4757-gmb-1678-4685-GMB-2016-0120-Suppl04.pdf]
