# Supplementary material for: Novel compound heterozygous mutations of ALDH1A3 contribute to anophthalmia in a non-consanguineous Chinese family
Source: Genet Mol Biol. 2017 Jun 5;40(2):430–5. doi: 10.1590/1678-4685-GMB-2016-0120 (PMC5488456; doi:10.1590/1678-4685-GMB-2016-0120)
Supplement: Supplementary file 5 [file 1415-4757-gmb-1678-4685-GMB-2016-0120-Suppl05.pdf]

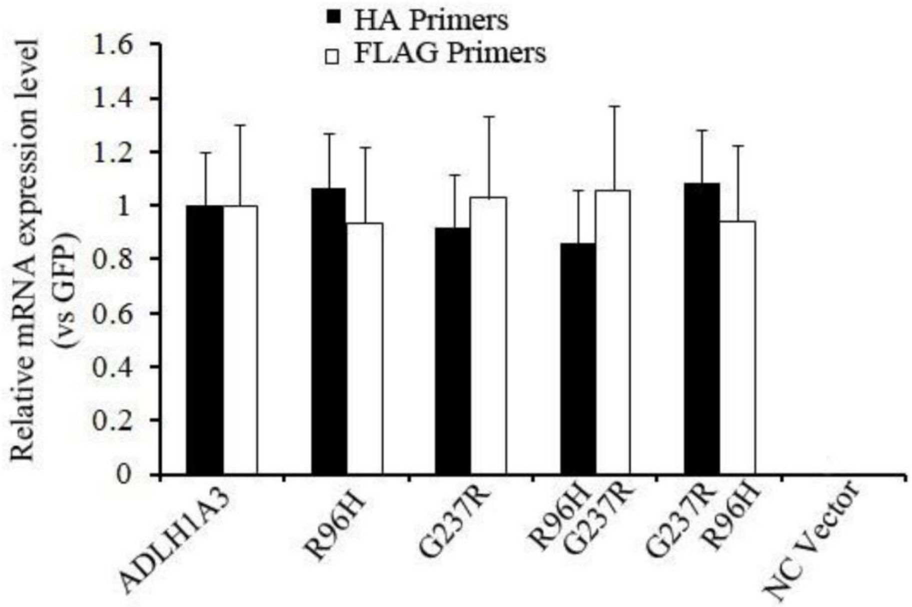

Figure S2 - The relative mRNA expression levels of WT *ALDH1A3* and mutant *R96H* and *G237R* in 293T cell was evaluated using quantitative reverse transcription PCR analysis. The expression levels were normalized using ΔCt (Ct_target – Ct_GFP). The quantitative data were expressed as the means ± standard deviation (S.D.) obtained from three independent experiments. The sequences of HA and FLAG primers are the following. 5′-AAATGTCAGGAAATGGCAGAGAAC-3′ (HA-f), 5′-GTAGCTAGCCATAACCACTTTGTA -3′ (HA-r), 5′-TTGGTGGCTTTAAAATGTCAGGAA-3′ (FLAG-f) and 5′-TTGTCGTCATCGTCTTTGTAGTCG-3′ (FLAG-r). The GFP primers: 5′-CCCGCGCCGAGGTGAAGT-3′ (Forward) and 5′-GACGTTGTGGCTGTTGTAGTTGTA-3′ (Reverse).
